# The use and operationalization of “structural stigma” in health-related research: A scoping review

**DOI:** 10.1186/s12889-024-21171-8

**Published:** 2024-12-30

**Authors:** Evan L. Eschliman, Edwina P. Kisanga, Long Jie Huang, Ohemaa B. Poku, Becky L. Genberg, Danielle German, Sarah M. Murray, Lawrence H. Yang, Michelle R. Kaufman

**Affiliations:** 1 Department of Epidemiology, Columbia University Mailman School of Public Health, New York, NY USA; 2 Department of Health, Behavior and Society, Johns Hopkins Bloomberg School of Public Health, Baltimore, MD USA; 3 Department of Educational and Counseling Psychology, University at Albany, State University of New York, Albany, NY USA; 4 Department of Psychiatry, Columbia University and New York State Psychiatric Institute, New York, NY USA; 5 Department of Epidemiology, Johns Hopkins Bloomberg School of Public Health, Baltimore, MD USA; 6 Department of Mental Health, Johns Hopkins Bloomberg School of Public Health, Baltimore, MD USA; 7 Department of Social and Behavioral Sciences, New York University School of Global Public Health, New York, NY USA; 8 Department of International Health, Johns Hopkins Bloomberg School of Public Health, Baltimore, MD USA

**Keywords:** Stigma, Discrimination, Structural factors, Scoping review, Quantitative, Qualitative

## Abstract

**Background:**

Research that investigates the negative health effects of stigma beyond the individual and interpersonal levels is increasingly using the concept of “structural stigma.” This scoping review investigates how the concept of “structural stigma” has been used and operationalized in health-related literature to date in order to characterize its usage and inform future operationalizations.

**Methods:**

A systematic search and screening process identified peer-reviewed, English-language research articles that used the term “structural stigma” available prior to January 1, 2024 in five databases (i.e., PubMed, PsycINFO, Embase, Web of Science, CINAHL).

**Results:**

Of the 298 articles identified, over half (53%) were published from 2021 onward. Articles most commonly were set in the United States (*n* = 163, 55%), investigated stigma toward sexual minority people (*n* = 163, 55%), and cited the introduction of a special issue of *Social Science & Medicine* as their source of the concept (*n* = 84, 28%). Most articles (64%) used at least one additional conceptual framework, most commonly minority stress theory (*n* = 107, 36%). Quantitative operationalizations (*n* = 102) engaged most in the conceptual domain of laws and government-level policies, while qualitative operationalizations (*n* = 68) engaged most with institutional (i.e., non-government-level) policies, practices, and procedures.

**Conclusions:**

As the use of “structural stigma” is increasing, operationalizations can better leverage the concept’s breadth and account for individuals’ intersectional lived experiences. This will necessitate bridging across methodologies and bodies of research on related negative social processes.

**Supplementary Information:**

The online version contains supplementary material available at 10.1186/s12889-024-21171-8.

## Introduction

People who experience the negative health effects of structures have long known the importance of and advocated for structural transformation. “Structure” as a term in social science and health-related research can refer to a variety of entities and concepts including culture, social norms, and public attitudes, as well as the policies, practices, and procedures of governments, organizations, and institutions. Since the turn of the 20th century, Du Bois [1] and many other scholars working decades later have developed a variety of terms, concepts, and theoretical frameworks to investigate how structures generate and perpetuate health inequities. Many multi-level frameworks and models (e.g., those based on the social ecological model [2] and in ecosocial approaches [3]) highlight the interrelations between macro-level or distal factors and more proximal (e.g., meso-level, micro-level) factors in shaping health [4]. Many other theoretical perspectives engaged in health-related research, such as fundamental cause theory [5, 6], syndemic theory [7], structural violence [8, 9], structural vulnerability [10, 11], and intersectionality [12–15] underscore the pervasiveness, embeddedness, and complexity of structural factors.

Despite this scholarship, it is relatively recently that the structural realm has been formally brought into academic models of stigma [16]. Stigma is a negative social process that worsens the health and well-being of people with a wide variety of othered identities, attributes, and health conditions [17]. Although “stigma” has definitional variations and inconsistencies [18, 19], many conceptualizations consider it to be multilevel in nature with some differentiation between intrapersonal, interpersonal, and structural levels that exist along a power gradient [18, 20] and are at interplay with the other levels [21]. Growing scholarly attention is also being given to how this multilevel “stigma complex” [22], “stigma machine” [23], or “stigma system” [24] is often toward more than one identity, attribute, or health condition, meaning stigma is frequently intersectional [25, 26]. At the most macro level lies the concept of “structural stigma,” which helps account for the reality that stigma is also produced and reproduced through forces and factors beyond interpersonal interactions [27].

Like the broader concept of stigma, a fair amount of variation exists in the definition and operationalization of “structural stigma,” which can create ambiguity in what conceptual domains of factors or processes it encompasses. One prominent early, if not the earliest, mention of structural stigma was by Link & Phelan (2001), who alluded to “structural” as a catch-all level for stigma processes occurring beyond the intrapersonal and interpersonal. In the definition provided in the 2014 special issue of *Social Science & Medicine* on structural stigma, three conceptual domains are specified: “societal-level conditions, cultural norms, and institutional practices that constrain the opportunities, resources, and wellbeing for stigmatized populations” [28]. Some definitions are narrower, however, such as the law- and policy-focused definition of Pescosolido & Martin (2015): “prejudice and discrimination by policies, laws, and constitutional practice.” Researchers have also used a variety of complementary conceptual frameworks to enrich the concept and provide more step-by-step delineations from the macro to the micro. For example, research focused on structural stigma and the health and well-being of sexual minority—and sometimes also gender minority—individuals has often engaged minority stress theory [29, 30] and its extensions [31–33].

Past narrative reviews or overviews of structural stigma have primarily focused on one population, health outcome, and/or methodology [27, 34–36]. In order to broadly characterize the use of the structural stigma concept and provide a resource for informing future operationalizations, this scoping review seeks to answer the following research question: How has the term “structural stigma” been used and operationalized in health-related peer-reviewed empirical research, regardless of population, outcome, or method? A synthesis of the ways structural stigma has been operationalized across health-related research can provide public health and related fields with an improved ability to further examine and address structural stigma’s negative effects on health and well-being.

## Methods

### Search strategy

The search syntax “structural stigma*” was used to identify peer-reviewed research articles containing the term “structural stigma” in five databases (i.e., PubMed, PsycINFO, Embase, Web of Science, and CINAHL). The search was conducted on 1 September 2022 without date restriction and was updated on 1 January 2024; both the original and updated search were conducted by the first author (ELE).

### Title and abstract screening

Using the Covidence online review management software [37], two reviewers screened all titles and abstracts for eligibility for the original (ELE and EPK) and updated (ELE and LJH) search. Records were excluded if both reviewers agreed that the record was: not an empirical study (e.g., a commentary, review), not a peer-reviewed article (e.g., an abstract, book chapter, dissertation), not in English, or not referring to structural stigma as a social process. If either reviewer believed a decision could not be confidently ascertained, the record was advanced to full-text review.

### Full-text review and data charting

The same reviewer pairs reviewed the full text of each of the retained articles. Articles were eligible for inclusion if they were peer-reviewed research articles written in English that included the term “structural stigma” anywhere in the main text (i.e., not the abstract or citations). One reviewer (ELE) then charted data from all included articles. As a quality check, another reviewer (EPK or LJH) reviewed the data charting for 20% of the included articles. Data charted for each article were: (1) author(s) and year published; (2) setting (i.e., country or countries in which the research was sited); (3) sample; (4) stigmatized status(es) of interest; (5) definition of structural stigma used, if any; (6) cited source of the “structural stigma” concept, if any; (7) additional theories or conceptual frameworks used for analysis or contextualization of results; (8) objective; and (9) notes about the article’s operationalization of structural stigma, if operationalized.

### Analysis

First, articles were divided into three categories based on methodology: (1) quantitative operationalizations (i.e., if any construct used in its quantitative analyses was either named structural stigma or was explicitly explained to be representing or reflecting structural stigma); (2) qualitative operationalizations (i.e., if structural stigma was a qualitative theme or subtheme or named as part of the analysis’s conceptual framework); and (3) non-operationalizations. Multi- and mixed-method articles could be categorized under quantitative and/or qualitative operationalizations.

Second, articles that operationalized structural stigma were categorized based on the macro-level conceptual domain(s) with which the operationalization engaged. Three main conceptual domains emerged during data charting: (1) laws and government-level policies; (2) sociocultural attitudes and norms; and (3) institutional (i.e., non-government-level) policies, practices, and procedures. A fourth category of “additional structural factors” was used for engagement in macro-level factors that did not neatly fit into one of these three domains (e.g., media, election results).

### Protocol and registration

This scoping review report includes all required elements of the Preferred Reporting Items for Systematic reviews and Meta-Analyses extension for Scoping Reviews (PRISMA-ScR) checklist [38]. A completed PRISMA-ScR checklist is provided in Additional File 1. A registered protocol can be accessed at https://osf.io/jv3e6.

## Results

### Search, title and abstract screening, and full-text review

**Fig. 1:**
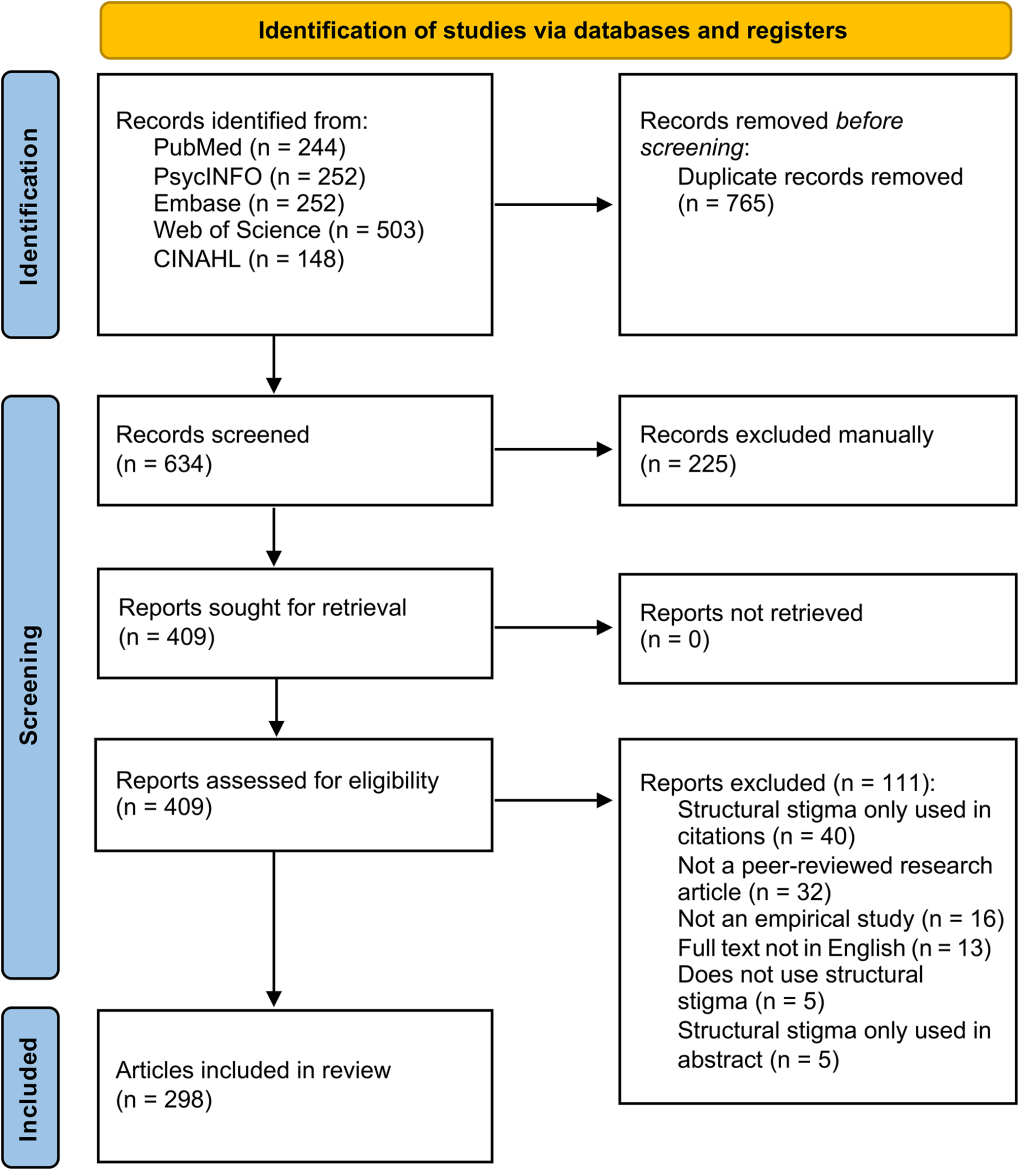
PRISMA flow diagram

Results of the searches, title and abstract screening, and full-text review are summarized in the PRISMA flow diagram (Fig. 1). Of the 634 unique records, 225 were excluded after title and abstract screening, and 111 were excluded during full-text review (i.e., 40 because “structural stigma” was only in the citations; 32 were not peer-reviewed research; 16 were not empirical studies; 13 were not in English; five did not use the term at all; and five used the term only in the abstract).

### Characteristics of included articles

A total of 298 articles were included. Just over one third of the articles quantitatively operationalized structural stigma (*n* = 102, 34%), while almost one-fourth were qualitative operationalizations (*n* = 68, 23%). Three articles (1%) both quantitatively and qualitatively operationalized structural stigma. Non-operationalizations were most common, but made up less than half of the articles (*n* = 131, 44%).

**Fig. 2:**
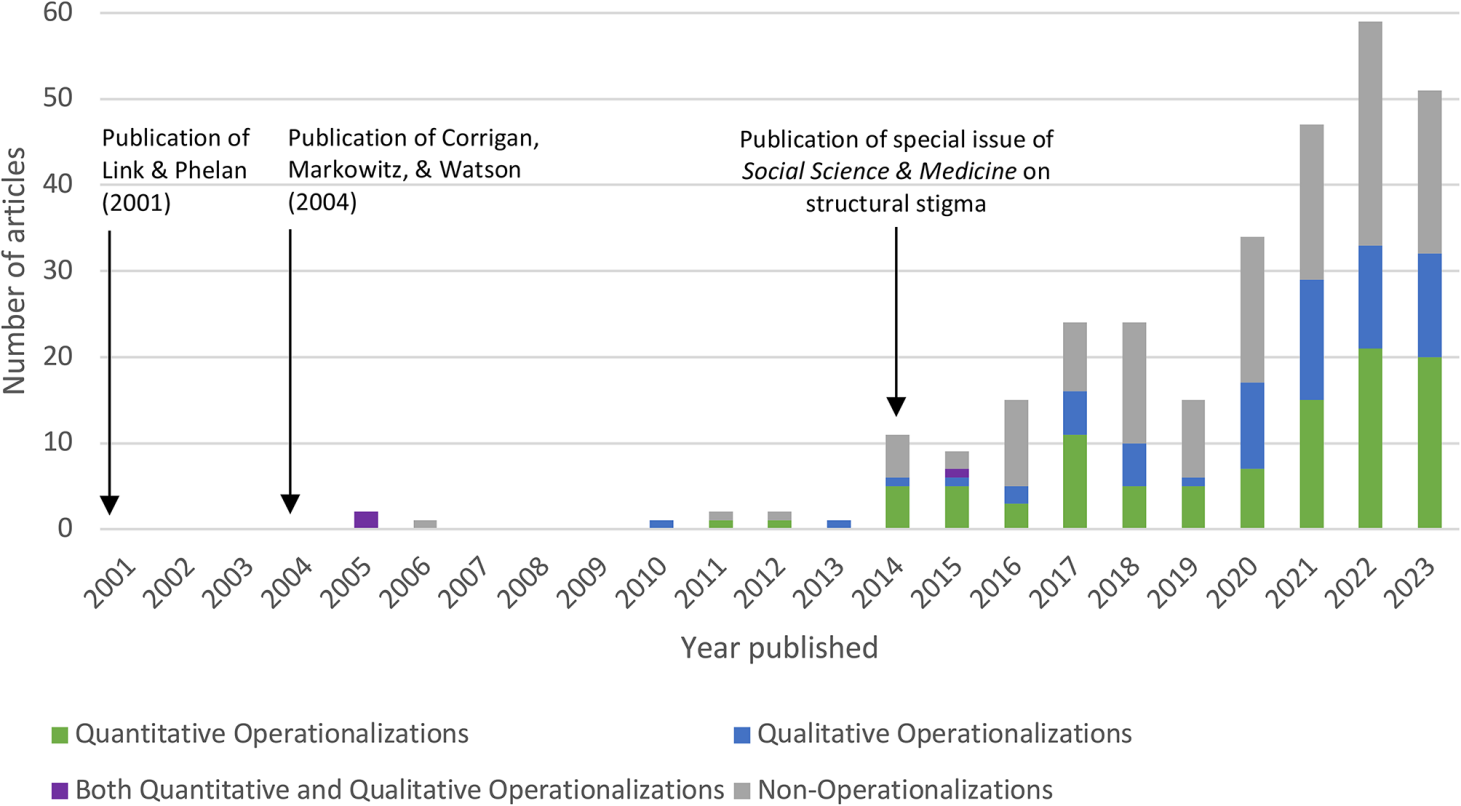
Number of articles that contain “structural stigma” by year published and by operationalization type

Figure 2 presents the number of articles published by year and by operationalization type, noting the timing of three formative publications related to structural stigma. Only nine (3%) articles were published prior to 2014 (i.e., when the special issue of *Social Science & Medicine* was published) and over half (*n* = 157, 53%) were published from 2021 to 2023.

Table 1 presents characteristics of all articles and separately by operationalization type. Of all articles identified, the majority (*n* = 163, 55%) reported on research conducted in the United States (U.S.). Over one-third (*n* = 107, 36%) of articles focused solely on stigma related to sexual minority status, and over half focused on sexual minority status, gender minority status, or both (*n* = 159, 53%). A relatively sizeable representation of articles looked at mental health either alone (*n* = 32, 11%) or with additional statuses (*n* = 3, 1%), as well as HIV either alone (*n* = 13, 4%) or with additional statuses (*n* = 11, 4%). Race/ethnicity as a stigmatized status was more often investigated in combination with other statuses (*n* = 13, 4%) than alone (*n* = 3, 1%).

**Table 1:** Characteristics of included articles (*N* = 298)

Characteristic	Number of articles (*n*, %)			
	Out of total (*N* = 298)	Out of quantitative operationalizations (*N* = 102)	Out of qualitative operationalizations (*N* = 68)	Out of non-operationalizations (*N* = 131)
Setting				
United States	163, 55	57, 56	29, 43	80, 61
Multi-country, Europe	20, 7	17, 17	1, 1	2, 2
Canada	17, 6	0, 0	12, 18	5, 4
Australia	15, 5	6, 6	2, 3	7, 5
Multi-continent	12, 4	4, 4	5, 7	3, 2
Israel	5, 2	2, 2	3, 4	0, 0
United Kingdom	5, 1	1, 1	1, 1	3, 2
Italy	4, 1	2, 2	0, 0	2, 2
Singapore	4, 1	0, 0	3, 4	1, 1
Sweden	4, 1	3, 3	0, 0	1, 1
Taiwan	4, 1	1, 1	0, 0	3, 2
India	3, 1	0, 0	0, 0	3, 2
Brazil	2, 1	1, 1	0, 0	1, 1
China	2, 1	2, 2	0, 0	0, 0
Germany	2, 1	0, 0	0, 0	2, 2
Hong Kong	2, 1	0, 0	0, 0	2, 2
Lebanon	2, 1	0, 0	1, 1	1, 1
Romania	2, 1	1, 1	0, 0	1, 1
South Africa	2, 1	0, 0	0, 0	2, 0
Uganda	2, 1	1, 1	1, 1	0, 0
Zambia	2, 1	0, 0	2, 3	0, 0
Other single country^a^	18, 6	3, 3	6, 9	9, 7
Other multi-country^b^	3, 1	1, 1	0, 0	2, 2
N/A^c^	3, 1	0, 0	2, 3	1, 1
Stigmatized status(es) of interest				
Single status (*n* = 227)				
Sexual minority status	107, 36	52, 51	4, 6	51, 39
Mental health	32, 11	10, 10	14, 21	10, 8
Gender minority status	19, 6	9, 9	5, 7	5, 4
HIV	13, 4	2, 2	5, 7	6, 5
Substance use	8,3	1, 1	7, 10	0, 0
Dementia	7, 3	4, 4	2, 3	1, 1
Immigrant status or displacement	5, 2	2, 2	1, 1	2, 2
Welfare receipt	4, 2	1, 1	1, 1	2, 2
Abortion	3, 1	0, 0	2, 3	1, 1
Race/ethnicity	3, 1	1, 1	0, 0	2, 1
Sexual violence	3, 1	2, 2	1, 1	0, 0
Age	2, 1	2, 2	0, 0	0, 0
Disability	2, 1	0, 0	0, 0	2, 2
Gender	2, 1	0, 0	1, 1	1, 1
Polyamory	2, 1	0, 0	1, 1	1, 1
Sex work	2, 1	0, 0	2, 3	0, 0
Space	2, 1	0, 0	0, 0	2, 2
Other single status^d^	11, 4	0, 0	7, 10	4, 3
Multiple statuses (*n* = 71)				
Sexual minority status, gender minority status	33, 11	7, 7	7, 10	19, 15
HIV, race/ethnicity, sexual minority status	3, 1	0, 0	0, 0	3, 2
Immigrant status or displacement, sexual minority status	3, 1	1, 1	0, 0	2, 2
Race/ethnicity, sexual minority status	3, 1	1, 1	0, 0	2, 2
Gender, sexual minority status	2, 1	0, 0	0, 0	2, 2
Hepatitis C, substance use	2, 1	0, 0	2, 3	0, 0
HIV, sex work	2, 1	0, 0	1, 1	1, 1
HIV, sexual minority status,	2, 1	0, 0	1, 1	1, 1
Race/ethnicity, sexual minority status, gender minority status	2, 1	1, 1	1, 1	0, 0
Other multiple statuses^e^	19, 6	6, 6	3, 4	11, 8
Definition given				
Yes	159, 53	74, 73	45, 66	42, 32
Only examples	27, 9	8, 8	3, 4	16, 12
No	112, 38	20, 20	20, 29	73, 56
Cited source of the structural stigma concept^e^				
Hatzenbuehler & Link 2014	84, 28	32, 31	20, 29	32, 24
Hatzenbuehler 2016	40, 13	21, 21	4, 6	15, 11
Hatzenbuehler 2014	16, 5	4, 4	2, 3	10, 8
Link & Phelan 2001	16, 5	7, 7	3, 4	6, 5
Hatzenbuehler et al. 2014 (retracted)	10, 3	2, 2	0, 0	8, 6
Corrigan, Markowitz, & Watson 2004	7, 2	2, 2	4, 6	1, 1
White Hughto, Reisner, & Pachankis 2015	7,2	2, 2	5, 7	0, 0
Hatzenbuehler 2017 (Handbook)	6, 2	3, 3	1, 1	2, 2
Hatzenbuehler, Phelan, & Link 2013	6, 2	4, 4	1, 1	1, 1
Corrigan, Watson, Heyrman et al. 2005	5, 2	2, 2	1, 1	2, 21
Hatzenbuehler, Jun, Corliss & Austin 2014	5, 2	1, 1	0, 0	4, 3
Pachankis et al. 2021	4, 1	0, 0	0, 0	4, 3
Corrigan, Watson, Gracia et al. 2005	3, 1	0, 0	2, 3	1, 1
Hatzenbuehler, Keyes, & Hasin 2009	3, 1	0, 0	1, 1	2, 2
Pachankis & Bränström 2018	3, 1	0, 0	0, 0	3, 2
Pachankis et al. 2015	3, 1	1, 1	0, 0	2, 2
Pachankis, Hatzenbuehler & Starks 2014	3, 1	0, 0	0, 0	3, 2
Bos et al. 2013	2, 1	0, 0	1, 1	1, 1
Corrigan & Kleinlein 2005	2, 1	1, 1	1, 1	0, 0
Corrigan, Kerr, & Knudsen 2005	2, 1	0, 0	2, 3	0, 0
Hannem & Bruckert 2012	2, 1	0, 0	2, 3	0, 0
Hatzenbuehler 2009	2, 1	0, 0	0, 0	2, 2
Hatzenbuehler 2017 (JCCAP)	2, 1	1, 1	0, 0	1, 1
Hatzenbuehler, McLaughlin, Keyes, & Hasin 2010	2, 1	0, 0	1, 1	1, 1
Link & Phelan 2014	2, 1	1, 1	1, 1	0, 0
Major, Dovidio, & Link 2018	2, 1	0, 0	1, 1	1, 1
Pescosolido & Martin 2015	2, 1	0, 0	2, 3	0, 0
Pincus 1996	2, 1	0, 0	1, 1	1, 1
Schulze & Angermeyer 2003	2, 1	1, 1	1, 1	0, 0
Yang et al. 2007	2, 1	1, 1	2, 3	0, 0
Other	74, 25	16, 16	29, 43	31, 24
No source cited	66, 22	17, 17	13, 19	37, 28
Additional theories or conceptual frameworks used^f^				
Minority stress	75, 25	28, 27	5, 7	42, 32
Minority stress, other	17, 6	7, 7	1, 1	9, 7
Intersectionality, minority stress	9, 3	2, 2	1, 1	6, 5
Intersectionality	6, 2	1, 1	3, 4	2, 2
Intersectionality, other	4, 1	0, 0	2, 3	2, 2
Social ecological model	4, 1	1, 1	2, 3	1, 1
Foucauldian thought	3, 1	0, 0	3, 4	0, 0
Intersectionality, intersectional stigma	3, 1	0, 0	3, 4	0, 0
Fundamental cause theory	2, 1	1, 1	0, 0	1, 1
Goffman (1963)	2, 1	0, 0	2, 3	0, 0
Intersectionality, minority stress, other	2, 1	1, 1	0, 0	1, 1
Minority stress, sexual stigma conceptual framework	2, 1	1, 1	1, 1	0, 0
Minority stress, social ecological model	2, 1	0, 0	0, 0	2, 2
Sexual stigma conceptual framework	2, 1	2, 2	0, 0	0, 0
Territorial stigmatization	2, 1	0, 0	0, 0	2, 2
White Hughto et al.’s (2015) multilevel model of stigma toward trans people	2, 1	0, 0	2, 3	0, 0
Other single conceptual framework	36, 12	9, 9	10, 15	17, 13
Other multiple conceptual frameworks	20, 7	3, 3	9, 13	9, 7
N/A	105, 35	46, 45	24, 35	37, 28

Handbook = The Oxford Handbook of Stigma, Discrimination, and Health; JCCAP = Journal of Clinical Child and Adolescent Psychology

^a^ Includes: Afghanistan, Bangladesh, Barbados, Colombia, Czechia, Democratic Republic of the Congo, France, Indonesia, Ireland, Malaysia, Nigeria, Poland, Rwanda, Senegal, South Korea, Tanzania, and Thailand

^b^ Includes: Multi-country (Africa), Multi-country (Asia-Pacific), and Multi-country (North America)

^c^ Includes: Analysis of Reddit data, Analysis of documentary films, and Analysis of text of scientific research articles

^d^ Includes: Albinism, Autism, Cancer, Chronic pain, COVID-19, Fatness, Incarceration, Non-dominant language use, Occupation (coaching), Parkinson’s disease, and Use of local foods and medicines

^e^ Includes: Age and disability; Age and sexual minority status; Gender minority status and abortion; Gender minority status and veteran status; Gender, immigrant status, sexual minority status, and socioeconomic status; Gender and race/ethnicity; General structural stigma; HIV and gender (motherhood); HIV and incarceration; HIV and PrEP use; HIV, sexual minority status, and substance use; Immigrant status and mental health; Mental health and race/ethnicity; Mental health and substance use; Race/ethnicity, sexual minority status, and disability; Race/ethnicity, sexual minority status, and gender; Race/ethnicity, sexual minority status, smoking, and socioeconomic status; Race/ethnicity, sexual minority status, and socioeconomic status; and Sexual minority status, gender minority status, and age

^e^ “Other” sources cited are provided in Additional File 2

^f^ “Other” theories or conceptual frameworks used are provided in Additional File 2

Nearly half of the articles (*n* = 139, 47%) did not provide a definition of structural stigma, instead giving examples, providing a citation, and/or assuming a shared understanding. Over three-quarters of the articles cited at least one source for the concept of structural stigma (*n* = 232, 78%), with Hatzenbuehler & Link’s (2014) introduction to the special issue of *Social Science & Medicine* on structural stigma being the most cited source (*n* = 84, 28%). Most articles (*n* = 194, 65%) used at least one additional conceptual framework in their analysis. Minority stress theory was most common, used either alone or with another framework (*n* = 107, 34%). Intersectionality was the second most common theory or conceptual framework, used either alone or in combination (*n* = 24, 8%).

### Conceptual domains engaged in included articles

**Fig. 3:**
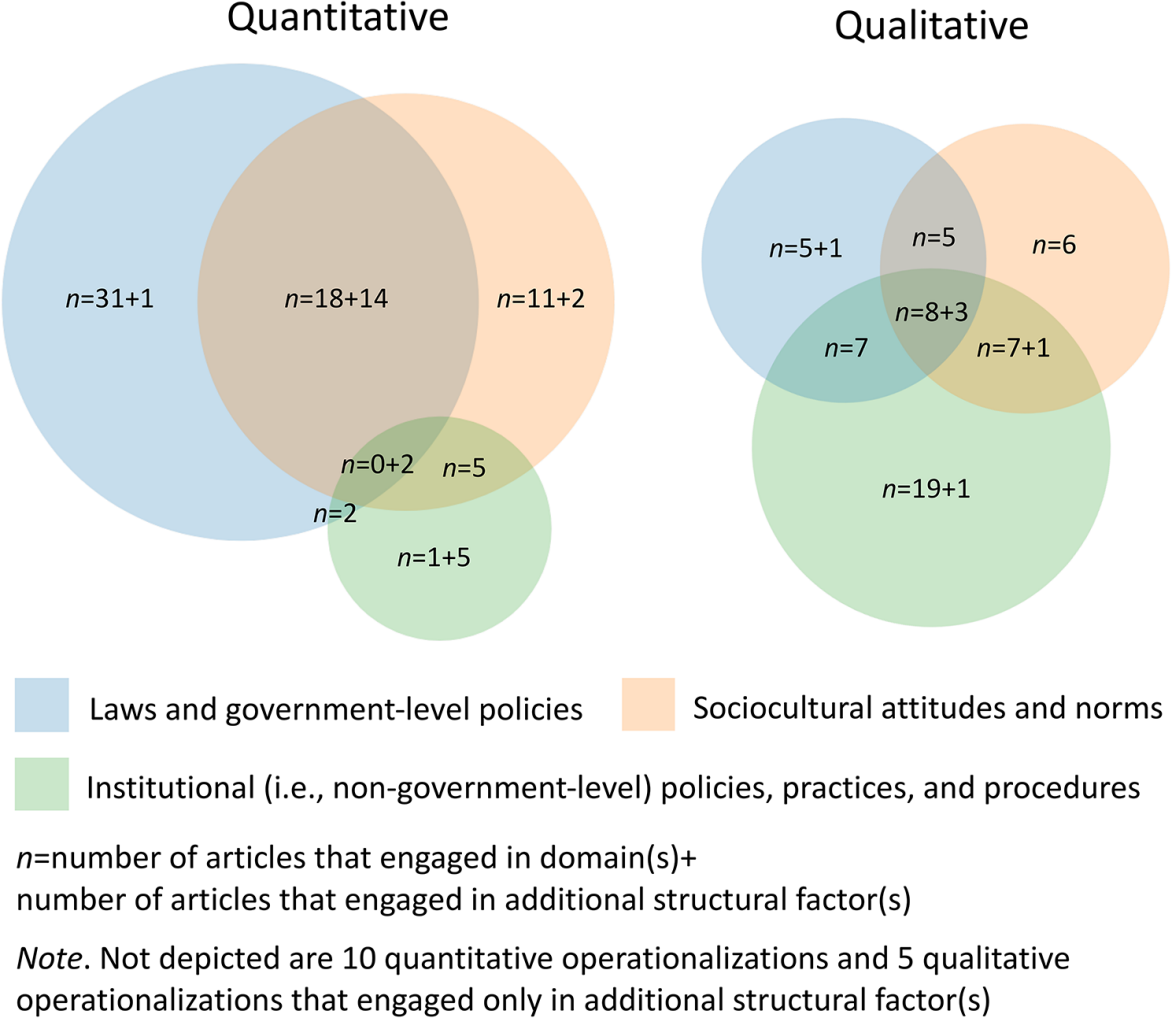
Operationalizations of structural stigma in identified articles by conceptual structural domain(s) engaged

Figure 3 shows engagement with the three main conceptual domains and their overlaps. Of the 167 operationalizations, the majority (*n* = 96, 57%) engaged with laws and government-level policies; nearly half (*n* = 82, 49%) engaged with sociocultural attitudes and norms; and just over one-third (*n* = 60, 36%) engaged with institutional (i.e., non-government-level) policies, practices, and procedures. Just over one-fifth of these articles (*n* = 36, 22%) engaged with one or more additional structural factors outside of the three main domains.

### Quantitative operationalizations

Details about the 102 articles that quantitatively operationalized structural stigma sorted by conceptual structural domain(s) engaged are given in Additional File 3, Additional Table 1. Compared to the total set of articles, multi-country European articles were proportionately more represented in quantitative operationalizations (*n* = 17, 17%), as were investigations of sexual minority health (*n* = 64, 63%). A substantial subset of quantitative operationalizations (*n* = 39, 38%) used minority stress theory and its extensions.

The most engaged domain among quantitative operationalizations was laws and government-level policies (*n* = 68, 67%). Quantitative operationalizations that engaged laws and government-level policies as well as sociocultural attitudes and norms (including those engaged in additional structural factors, *n* = 14) were the most common type of multi-domain operationalization (*n* = 32, 19% of all operationalizations). Engagement with laws and government-level policies most often involved creating or using scales or index measures of multiple laws and government-level policies related to sexual minority and/or gender minority populations (e.g., from the International Lesbian, Gay, Bisexual, Trans and Intersex Association, the Movement Advancement Project’s Equality Index). Some articles also engaged in this domain by operationalizing structural stigma as the presence or absence—or the time since the presence or absence—of single laws or policies (e.g., same-sex marriage or relationship recognition laws, healthcare denial policies), or individuals’ awareness or perception of laws or ongoing legislation (e.g., awareness of legislation banning transgender youth from participation in sports). Three articles employed structural stigma to conduct quantitative content analyses of legislation text.

Sociocultural norms and attitudes were most often measured as “social attitudes” using existing multinational, national, or other large-scale surveys (e.g., the American National Election Survey, the Eurobarometer, Project Implicit). These measures were often aggregated to the spatial unit of analysis, then used in the construction of standardized composite index scores alongside measures of laws and policies. This aggregation of existing data also occurred in the quantitative operationalizations that engaged solely with sociocultural attitudes and norms (*n* = 11, 11%), though a handful of these articles used study-specific participant response scales rather than existing large-scale surveys.

Almost all quantitative engagement with institutional policies, practices, and procedures was related to healthcare (e.g., private insurance protections for trans people), while also engaging another conceptual structural domain and/or additional structural factor(s) (e.g., occupational stress and help-seeking among healthcare professionals). Outside of healthcare, other institutional policies, practices, and procedures included stated beliefs of religious institutions, perceived acceptance of sexual minority individuals in civic institutions, and perceptions of institutional support provided to women and girls following sexual exploitation or abuse.

Additional structural factors engaged among quantitative operationalizations of structural stigma included factors related to sexual and gender minority people (e.g., the density of same-sex partner households, the proportion of gay-straight [or gender-sexuality] alliances among public high schools); additional factors in other indices (e.g., the Racial Equity Index, the Gender Equality Index); the use of structural stigma for quantitative content analyses of news media articles; and the use of election results, hypothetical budgeting, and social network measures as proxies for structural stigma.

### Qualitative operationalizations

Details about the 68 articles that qualitatively operationalized structural stigma are provided in Additional File 3, Additional Table 2. Relative to articles that quantitatively operationalized structural stigma, qualitative operationalizations occurred across more varied settings, explored more stigmatized statuses of interest, referenced a greater variety of sources for the concept of structural stigma, and employed a larger number of additional theories and conceptual frameworks. Unlike quantitative operationalizations and the articles overall, which focused most on sexual minority status, mental health was the most common stigmatized status of interest (*n* = 15, 22%).

Qualitative engagement with laws and government-level policies and sociocultural attitudes and norms were roughly equally common (*n* = 29, 43% and *n* = 30, 44%, respectively). Over two-thirds of qualitative operationalizations engaged with institutional policies, practices, and procedures (*n* = 46, 68%), as opposed to 16% of quantitative operationalizations. Eleven qualitative operationalizations (16%) engaged with additional structural factors.

Almost all qualitative operationalizations engaging laws and governmental policies (*n =* 24 out of *n* = 29, 83%) also engaged with another conceptual structural domain and/or additional factor(s). Approaches to engaging this domain included considering topic-specific laws and government-level policies as an element of an overall stigmatizing structural context; investigating or documenting individuals’ frustrations with and/or navigations of specific laws or policy initiatives; and investigating anticipated structural stigma related to U.S. presidential administration changes.

Few qualitative operationalizations that engaged with sociocultural attitudes and norms engaged solely with this domain (*n =* 6 out of *n* = 30, 20%), most often naming or investigating broad public or community-level attitudes or norms as a part of structural stigma. About half of this subset of articles engaged at least in part with structural stigma toward sexual minority people using varying terms (e.g., homophobia, heterosexism). It was fairly common in this subset of articles for a specific system of oppression (e.g., racism, ableism, cisheterosexism) to be named in the explanation of the analytic framework, results, or discussion. Some articles also narrowed specifically to religious and cultural norms and their interrelated nature, or included a focus on specific stereotypes, forms of rhetoric, or misconceptions (e.g., sex work as “risky”; rape myths).

Like the quantitative studies, most of the qualitative engagement with institutional policies, practices, and procedures focused at least in part on healthcare (*n* = 30 out of *n* = 46, 65%). Many qualitative operationalizations engaging this domain included investigations of people’s experiences of discrimination from professionals within institutions (e.g., healthcare professionals [39–44], police officers [40, 42, 45–47], employers [48–50]), while a handful examined these structural actors’ perceptions and experiences of institutional policies, practices, and procedures.

Additional structural factors engaged in qualitative operationalizations included media (e.g., newspaper articles, documentaries), elections, research funding, and religion.

### Non-operationalizations

Articles that mentioned, but did not operationalize, structural stigma were the most common type of articles identified. These articles are detailed in Additional File 3, Additional Table 3. These articles had more variation in stigmatized statuses of interest compared to the other two operationalization types, with 35 unique groupings of stigmatized statuses of interest. This set also had the greatest count of studies that explored race/ethnicity as a stigmatized status of interest (*n* = 11). Most commonly, these articles would mention structural stigma in either the introduction or discussion as a relevant concept or area of research related to the study rationale or interpretation of study findings. Some mentioned structural stigma while more often using another term to discuss structural stigma or another related process (e.g., structural discrimination) without ever explicitly mentioning how the concepts relate.

## Discussion

Overall, use of “structural stigma” is rapidly increasing in health-related research. The concept has been quantitatively and qualitatively operationalized and mentioned in articles investigating structural factors’ effects on the health of people with a range of stigmatized statuses. The three main conceptual domains identified in this review (i.e., laws and government-level policies; sociocultural attitudes and norms; and institutional policies, practices, and procedures) resemble those delineated in prior high-level summaries of research using the structural stigma concept [34] and in the most commonly cited definition of structural stigma [28]. At the same time, few articles individually assessed all three of the main conceptual domains, and some articles engaged factors outside of these three domains. A definition inclusive of all uses of structural stigma in this review could describe structural stigma as the most macro-level of an entrenched and wide-reaching system of intersectional stratification and marginalization that negatively influences how one navigates and establishes relationships with structures, others, and self across the lifecourse and the contexts in which one lives.

Although the most common type of operationalization of structural stigma was quantitative engagement in both laws and government-level policies and sociocultural attitudes and norms, the qualitative operationalizations in this review demonstrate the value in examining and incorporating individuals’ agency and lived experience in studies of structural stigma. Taken together, these operationalizations underscore how structural stigma spans the more overt to the more covert [34, 51], and that some key manifestations of structural stigma may only be evident through attending to the perspective of individuals who experience it [52–56]. For example, while almost no quantitative operationalizations assessed specific experiences of discrimination (with notable exceptions [40, 57]), participants in the qualitative studies identified in this review frequently spoke about discrimination from and specific interactions with structural actors such as healthcare professionals [39–44], police officers [40, 42, 45–47], and employers [48–50]. While some stigma-related research—including one study identified in this review [58]—may position these interactions solely at the interpersonal level, the use of “structural stigma” to date suggests that structural stigma can be felt and experienced through interactions with these structural actors. This also highlights that, in addition to the imperative of changing and transforming stigmatizing structures themselves [59], interventions that support and equip structural actors with training and resources may help to mitigate the felt effects of structural stigma [60].

It was uncommon for articles to engage only in sociocultural attitudes and norms, perhaps because factors can be positioned at the interpersonal level, can be referred to via terms such as “public stigma” or “social stigma,” and are sometimes viewed as closely related consequences or indicators of structural stigma rather than an explicit form of it. The three quantitative operationalizations specifically looking at “culture” stand out as exemplars of how sociocultural attitudes and norms can be more explicitly linked to structural processes; two articles [61, 62] measured Confucian cultural values as a cross-cutting set of social values that shapes both culture and structure (e.g., laws and policies) and another used a culture-focused understanding of stigma (i.e., “What Matters Most” [63]) to help articulate how cultural factors were seen to both reflect and reciprocally reinforce and be reinforced by, structure [40]. Notably, there were related additional structural factors engaged (e.g., media, religion, and politics) that can shape—and be shaped by—sociocultural attitudes and norms; this encourages additional work to understand and assess structural stigma emanating from these sources.

Some relevant settings, populations, and structural conceptual frameworks were largely absent from this set of articles. The vast majority of studies being sited in U.S., Canada, and Australia highlights how the structural stigma concept has rarely if ever been used in research that focuses specifically on the health and well-being of Indigenous peoples in these contexts [64, 65]. There are also only a small group of English-language articles that use structural stigma to investigate health effects of systems of oppression operating in much of the Global South (e.g., gender, coloniality, resource extraction) [66–68]; this could be due to the English language limitation of this review, or could be suggestive of the concept’s limits when investigating these forces. Further, although examining structural stigma toward sexual and gender minority individuals was most common, articles focusing on gender minority people’s health—especially nonbinary and gender expansive people’s—were still rare, and no articles focused on intersex people’s health. There are also opportunities to contextualize structural stigma in the decades of sociological scholarship on the relationship between structure and agency [69, 70] and critically—especially for the nearly one-fourth of these articles that focused on people with more than one stigmatized status—engage more with intersectionality and intersectional stigma [25].

However, putting structural stigma in genuine conversation with related structural processes and conceptual frameworks will require ongoing interrogation of the commonalities and distinctions in these concepts and intentional bridging across bodies of research. For example, early articulations of “structural stigma” gave examples related to structural racism and racial discrimination (e.g., Jim Crow laws) and pointed out similarities between structural stigma and structural racism [18, 71, 72]; however, few articles operationalizing “structural stigma” to date appeared to investigate stigma related to race/ethnicity or include operationalizations of structural racism. There are myriad potential reasons for this apparent separation, ranging from the histories of stigma and discrimination research [73, 74] to the real benefits of using more specific terminology to call out and interrogate specific systems of oppression [13]. Regardless, there is likely much to be gained from intentional efforts to ensure structural stigma research stays attuned to the robust existing scholarship that operationalizes structural racism [75–78] and the many other related concepts (e.g., structural sexism [79, 80]), especially when investigating intersectional stigma [81]. There are advantages to using a relatively broad concept like structural stigma to provide common language and search terms and facilitating cross-pollination of methods and findings, while also naming and recognizing the related, more specific—or in some cases, broader (e.g., coloniality)—processes at hand and their often-crucial distinctions. As the diffusion, operationalization, delineation, and reification of the structural stigma concept continues, the ways this concept is or is not suited to be an umbrella concept—and why—will need to be a priority for the research discourse.

### Limitations

This scoping review only sought to characterize the use and operationalization of “structural stigma,” and thus it included, but did not search for, articles using related terms; this review can be read alongside others that focus on these highly relevant similar negative social forces [65, 76] while the field navigates how or to what extent these many related concepts and terms can be used alongside each other. Further, although five databases were searched, the set of included articles may not be an exhaustive list of all health-related empirical research articles that have used the concept of structural stigma. The search strategy and the review’s inclusion criteria also restricted analysis to articles in English; this means any non-English language scholarship using this concept was not represented, in turn potentially limiting the range and counts of settings represented in the identified articles. Moreover, because this review focused on broad characterization of the usage and operationalization of the structural stigma concept, a variety of research questions were left unanswered. These include questions related to these studies’ specific analytical strategies (e.g., how relatively few studies engaged spatial and longitudinal methods to account for differential exposures to structural stigma across the lifecourse [82] as individuals move between structural contexts [83], or had analyses testing for biophysical consequences [3, 84]), results (e.g., the strengths of observed quantitative associations), and chosen approaches to the measurement of structural stigma. In-depth critiques of results and measurement may be more appropriate for more specific reviews, such as on LGBTQ + health [36]. Future research on the concept could also take on other critically important features of this body of research observed in the process of this review, such as how infrequently structural stigma was placed in historical context and how rarely overarching economic systems were implicated in the production of structural stigma.

## Conclusion

The concept of “structural stigma” is increasingly being used in health-related research investigating varied systems of oppression on the bases of varied stigmatized statuses, and its operationalizations tend to engage three main conceptual domains of structural factors. As its use and operationalization continues, it remains critical to attend to the agency and lived experiences of people who experience structural stigma in their interactions with structures and structural actors, to interrogate how sociocultural attitudes and norms and related structural factors are or are not considered at the structural level, to consider who and what settings are under-included, and to build bridges across concepts related to structural forms of stigma and discrimination. Taking these steps can push structural stigma research into fulfilling more of its promise to make negative health effects of structures plainly visible to those in power, and ultimately, to urge structural transformation toward an imagined world of health and well-being for all.

## Electronic supplementary material

Below is the link to the electronic supplementary material.

Supplementary Material 1

Supplementary Material 2

Supplementary Material 3

## Data Availability

The datasets generated and/or analyzed during the current study are not publicly available but are available from the corresponding author on reasonable request.

## References

[1] Du Bois WEB. The Philadelphia Negro: a social study. Reprint edition. Philadelphia: University of Pennsylvania; 1996.

[2] White Hughto JM, Reisner SL, Pachankis JE. Transgender stigma and health: a critical review of stigma determinants, mechanisms, and interventions. Soc Sci Med. 2015;147:222–31. 26599625 10.1016/j.socscimed.2015.11.010 PMC4689648

[3] Krieger N. Methods for the Scientific Study of Discrimination and Health: an Ecosocial Approach. Am J Public Health. 2012;102:936–44. 22420803 10.2105/AJPH.2011.300544 PMC3484783

[4] Diez Roux AV. Ecological variables, ecological studies, and multilevel studies in public health research. In: Detels R, Gulliford M, Abdool Karim Q, Tan CC, editors. Oxford textbook of global public health. Sixth edition. Oxford: Oxford University Press; 2017.

[5] Clouston SAP, Link BG. A Retrospective on Fundamental cause theory: state of the literature and goals for the future. Annu Rev Sociol. 2021;47:131–56. 34949900 10.1146/annurev-soc-090320-094912 PMC8691558

[6] Link BG, Phelan J. Social conditions as Fundamental causes of Disease. J Health Soc Behav. 1995;:80–94. 7560851

[7] Singer M, Bulled N, Ostrach B, Mendenhall E. Syndemics and the biosocial conception of health. Lancet. 2017;389:941–50. 28271845 10.1016/S0140-6736(17)30003-X

[8] Farmer P. An Anthropology of Structural Violence. Curr Anthropol. 2004;45:305–25.

[9] Galtung J. Violence, peace, and Peace Research. J Peace Res. 1969;6:167–91.

[10] Bourgois P, Holmes SM, Sue K, Quesada J. Structural vulnerability: operationalizing the Concept to address Health disparities in Clinical Care. Acad Med. 2017;92:299–307. 27415443 10.1097/ACM.0000000000001294 PMC5233668

[11] Quesada J, Hart LK, Bourgois P. Structural vulnerability and health: latino migrant laborers in the United States. Med Anthropol. 2011;30:339–62. 21777121 10.1080/01459740.2011.576725 PMC3146033

[12] Bowleg L. Evolving Intersectionality within Public Health: from analysis to action. Am J Public Health. 2021;111:88–90. 33326269 10.2105/AJPH.2020.306031 PMC7750585

[13] Collins PH. Intersectionality’s definitional dilemmas. Annu Rev Sociol. 2015;41:1–20.

[14] Crenshaw K. Mapping the margins: Intersectionality, Identity Politics, and violence against women of Color. Stan L Rev. 1990;43:1241.

[15] Crenshaw K. Demarginalizing the Intersection of Race and Sex: A Black Feminist Critique of Antidiscrimination Doctrine, Feminist Theory and Antiracist Politics. U, Chi Legal F. 1989;1989:139.

[16] Link BG. Commentary on: sexual and Gender Minority Health Disparities as a social issue: how Stigma and Intergroup relations can explain and reduce Health disparities: sexual and gender Minority Health disparities. J Soc Issues. 2017;73:658–66.

[17] Hatzenbuehler ML, Phelan JC, Link BG. Stigma as a Fundamental cause of Population Health inequalities. Am J Public Health. 2013;103:813–21. 23488505 10.2105/AJPH.2012.301069 PMC3682466

[18] Link BG, Phelan JC. Conceptualizing Stigma. Annu Rev Sociol. 2001;27:363–85.

[19] Yang L, Kleinman A, Morita J, Principles. Stigma. In: International Encyclopedia of Public Health. 2nd edition. Elsevier; 2017. pp. 40–50.

[20] Link BG, Phelan J. Stigma power. Soc Sci Med. 2014;103:24–32. 24507908 10.1016/j.socscimed.2013.07.035 PMC4451051

[21] Cook JE, Purdie-Vaughns V, Meyer IH, Busch JTA. Intervening within and across levels: a multilevel approach to stigma and public health. Soc Sci Med. 2014;103:101–9. 24513229 10.1016/j.socscimed.2013.09.023

[22] Pescosolido BA, Martin JK. The stigma complex. Ann Rev Sociol. 2015;41:87–116. 26855471 10.1146/annurev-soc-071312-145702 PMC4737963

[23] Tyler I. Stigma: the machinery of inequality. London: Zed books; 2020.

[24] Friedman SR, Williams LD, Guarino H, Mateu-Gelabert P, Krawczyk N, Hamilton L, et al. The stigma system: how sociopolitical domination, scapegoating, and stigma shape public health. J Community Psychol. 2021. jcop.22581. 10.1002/jcop.22581 PMC8664901 34115390

[25] Berger MT. Workable sisterhood: the political journey of Stigmatized Women with HIV/AIDS. Princeton: Princeton University Press; 2004.

[26] Dale SK, Ayala G, Logie CH, Bowleg L. Addressing HIV-Related intersectional stigma and discrimination to Improve Public Health outcomes: an AJPH supplement. Am J Public Health. 2022;112:S335–7. 35763724 10.2105/AJPH.2022.306738 PMC9241474

[27] Hatzenbuehler ML. Structural stigma: Research evidence and implications for psychological science. Am Psychol. 2016;71:742–51. 27977256 10.1037/amp0000068 PMC5172391

[28] Hatzenbuehler ML, Link BG. Introduction to the special issue on structural stigma and health. Soc Sci Med. 2014;103:1–6. 24445152 10.1016/j.socscimed.2013.12.017

[29] Brooks VR. Minority stress and lesbian women. Lexington, Mass: Lexington Books; 1981.

[30] Meyer IH. Prejudice, social stress, and mental health in lesbian, gay, and bisexual populations: conceptual issues and research evidence. Psychol Bull. 2003;129:674–97. 12956539 10.1037/0033-2909.129.5.674 PMC2072932

[31] Testa RJ, Habarth J, Peta J, Balsam K, Bockting W. Development of the gender minority stress and resilience measure. Psychol Sex Orientat Gend Divers. 2015;2:65–77.

[32] Tan KKH, Treharne GJ, Ellis SJ, Schmidt JM, Veale JF. Gender minority stress: a critical review. J Homosex. 2020;67:1471–89. 30912709 10.1080/00918369.2019.1591789

[33] Frost DM. The Benefits and Challenges of Health Disparities and Social Stress Frameworks for Research on sexual and gender Minority Health: benefits and challenges. J Soc Issues. 2017;73:462–76.

[34] Hatzenbuehler ML. Structural Stigma and Health. In: Major B, Dovidio JF, Link BG, editors. The Oxford Handbook of Stigma, Discrimination, and Health. 1st edition. Oxford University Press; 2017. pp. 105–22.

[35] Hatzenbuehler ML, Lattanner MR, McKetta S, Pachankis JE. Structural stigma and LGBTQ + health: a narrative review of quantitative studies. Lancet Public Health. 2024;9:e109–27. 38307678 10.1016/S2468-2667(23)00312-2

[36] Lattanner MR, McKetta S, Pachankis JE, Hatzenbuehler ML. State of the Science of Structural Stigma and LGBTQ + health: Meta-Analytic evidence, Research Gaps, and future directions. Annu Rev Public Health. 2024. 10.1146/annurev-publhealth-071723-013336. 39531387 10.1146/annurev-publhealth-071723-013336 PMC11980029

[37] Veritas Health Innovation. Covidence systematic review software.

[38] Tricco AC, Lillie E, Zarin W, O’Brien KK, Colquhoun H, Levac D, et al. PRISMA Extension for scoping reviews (PRISMA-ScR): Checklist and Explanation. Ann Intern Med. 2018;169:467–73. 30178033 10.7326/M18-0850

[39] Antoniou T, Pritlove C, Shearer D, Tadrous M, Shah H, Gomes T. Accessing hepatitis C direct acting antivirals among people living with hepatitis C: a qualitative study. Int J Equity Health. 2023;22:112. 37280588 10.1186/s12939-023-01924-4 PMC10243011

[40] Cheng ZH, Tu M-C, Li VA, Chang RW, Yang LH. Experiences of Social and Structural forms of Stigma among Chinese immigrant consumers with psychosis. J Immigr Minor Health. 2015;17:1723–31. 25672991 10.1007/s10903-015-0167-3

[41] Earnshaw VA, Cox J, Wong PL, Saifi R, Walters S, Azwa I, et al. I want the doctors to know that I am as bright as a candle:: experiences with and hopes for doctor interactions among Malaysian key populations and people living with HIV. AIDS Behav. 2023;27:2103–12. 36472685 10.1007/s10461-022-03942-9 PMC9734400

[42] Ramos-Pibernus AG, Rivera-Segarra ER, Rodríguez-Madera SL, Varas-Díaz N, Padilla M. Stigmatizing experiences of Trans men in Puerto Rico: implications for Health. Transgender Health. 2020;5:234–40. 33381650 10.1089/trgh.2020.0021 PMC7759285

[43] Ritterbusch AE, Correa Salazar C, Correa A. Stigma-related access barriers and violence against trans women in the Colombian healthcare system. Glob Public Health. 2018;13:1831–45. 29583079 10.1080/17441692.2018.1455887

[44] Syvertsen JL, Toneff H, Howard H, Spadola C, Madden D, Clapp J. Conceptualizing stigma in contexts of pregnancy and opioid misuse: a qualitative study with women and healthcare providers in Ohio. Drug Alcohol Depend. 2021;222:108677. 33775446 10.1016/j.drugalcdep.2021.108677

[45] Childs E, Biello KB, Valente PK, Salhaney P, Biancarelli DL, Olson J, et al. Implementing harm reduction in non-urban communities affected by opioids and polysubstance use: a qualitative study exploring challenges and mitigating strategies. Int J Drug Policy. 2021;90:103080. 33340947 10.1016/j.drugpo.2020.103080 PMC8046716

[46] Davis S, Wallace B, Van Roode T, Hore D. Substance use stigma and community drug checking: a qualitative study examining barriers and possible responses. IJERPH. 2022;19:15978. 36498052 10.3390/ijerph192315978 PMC9740784

[47] Ugwu UT, Dumbili EW. Inhaling thick smoke: cannabis subculture, community forming and socio-structural challenges in Nigeria. Drugs: Educ Prev Policy. 2022;29:345–54.

[48] Mujugira A, Kasiita V, Bagaya M, Nakyanzi A, Bambia F, Nampewo O et al. You are not a man: a multi-method study of trans stigma and risk of HIV and sexually transmitted infections among trans men in Uganda. J Int AIDS Soc. 2021;24. 10.1002/jia2.25860 PMC8716065 34965322

[49] Stevens ME, Parsons JA, Read SE, Bond V, Solomon P, Nixon SA. The relationship between stigma and a rehabilitation framework [international classification of functioning, disability and health (ICF)]: three case studies of women living with HIV in Lusaka, Zambia. Disabil Rehabil. 2021;43:2149–56. 31766899 10.1080/09638288.2019.1693640

[50] Woodgate RL, Comaskey B, Tennent P, Wener P, Altman G. The wicked problem of Stigma for Youth living with anxiety. Qual Health Res. 2020;30:1491–502. 32484387 10.1177/1049732320916460

[51] Bonilla-Silva E. Racism without racists: Color-Blind Racism and the persistence of racial inequality in the United States. Rowman & Littlefield; 2006.

[52] Eschliman EL, Choe K, DeLucia A, Addison E, Jackson VW, Murray SM, et al. First-hand accounts of structural stigma toward people who use opioids on Reddit. Soc Sci Med. 2024;347:116772. 38502980 10.1016/j.socscimed.2024.116772 PMC11031276

[53] Ford JV, Dodge B, Clark KA, Lattanner MR, Shah A, Hatzenbuehler ML. (Re)conceptualizing structural stigma: insights from a qualitative study of sexual minority men in a longitudinal, population-based cohort. Stigma Health. 2024. 10.1037/sah0000571. 10.1037/sah0000571 PMC12407154 40910089

[54] Lane J. Working through Stigma: a Constructivist Grounded Theory of Delivering Health Services to diverse 2SLGBTQ populations. Qual Health Res. 2023;33:624–37. 37070574 10.1177/10497323231167828 PMC10259085

[55] Poteat T, German D, Kerrigan D. Managing uncertainty: a grounded theory of stigma in transgender health care encounters. Soc Sci Med. 2013;84:22–9. 23517700 10.1016/j.socscimed.2013.02.019

[56] Prowse G, Weaver VM, Meares TL. The state from below: distorted responsiveness in Policed communities. Urban Affairs Rev. 2020;56:1423–71.

[57] Wainberg ML, Cournos F, Wall MM, Norcini Pala A, Mann CG, Pinto D, et al. Mental illness sexual stigma: implications for health and recovery. Psychiatr Rehabil J. 2016;39:90–6. 27030909 10.1037/prj0000168 PMC4900913

[58] White Hughto JM, Clark KA, Altice FL, Reisner SL, Kershaw TS, Pachankis JE. Creating, reinforcing, and resisting the gender binary: a qualitative study of transgender women’s healthcare experiences in sex-segregated jails and prisons. Int J Prison Health. 2018;14:69–88. 29869582 10.1108/IJPH-02-2017-0011 PMC5992494

[59] Blake VK, Hatzenbuehler ML. Legal remedies to address stigma-based Health inequalities in the United States: challenges and opportunities. Milbank Q. 2019;97:480–504. 31087411 10.1111/1468-0009.12391 PMC6554507

[60] Nyblade L, Stockton MA, Giger K, Bond V, Ekstrand ML, Lean RM, et al. Stigma in health facilities: why it matters and how we can change it. BMC Med. 2019;17:25. 30764806 10.1186/s12916-019-1256-2 PMC6376713

[61] Pu Y, Xu W. Parenting Desire among sexual minority women in China: from the Stigma Perspective. Arch Sex Behav. 2023. 10.1007/s10508-023-02682-8. 37620669 10.1007/s10508-023-02682-8

[62] Xu W, Huang Y, Tang W, Kaufman MR. Heterosexual marital intention: the influences of Confucianism and Stigma among Chinese sexual minority women and men. Arch Sex Behav. 2022;51:3529–40. 35900678 10.1007/s10508-021-02229-9

[63] Yang LH, Kleinman A, Link BG, Phelan JC, Lee S, Good B. Culture and stigma: adding moral experience to stigma theory. Soc Sci Med. 2007;64:1524–35. 17188411 10.1016/j.socscimed.2006.11.013

[64] Bryant J, Bolt R, Botfield JR, Martin K, Doyle M, Murphy D, et al. Beyond deficit: ‘strengths-based approaches’ in indigenous health research. Sociol Health Illn. 2021;43:1405–21. 34145599 10.1111/1467-9566.13311

[65] Poirier B, Sethi S, Haag D, Hedges J, Jamieson L. The impact of neoliberal generative mechanisms on indigenous health: a critical realist scoping review. Global Health. 2022;18:61. 35705995 10.1186/s12992-022-00852-2 PMC9199313

[66] Connell R. Gender, health and theory: conceptualizing the issue, in local and world perspective. Soc Sci Med. 2012;74:1675–83. 21764489 10.1016/j.socscimed.2011.06.006

[67] Mignolo W. Local histories/global designs: coloniality, subaltern knowledges, and border thinking. Princeton University Press; 2012.

[68] Malin SA, Ryder S, Lyra MG. Environmental justice and natural resource extraction: intersections of power, equity and access. Environ Sociol. 2019;5:109–16.

[69] Guise A. Stigma power in practice: exploring the contribution of Bourdieu’s theory to stigma, discrimination and health research. Soc Sci Med. 2024;:116774. 10.1016/j.socscimed.2024.116774 38537331

[70] Williams GH. The determinants of health: structure, context and agency. Sociol Health Illn. 2003;25:131–54. 14498934

[71] Corrigan PW, Markowitz FE, Watson AC. Structural levels of Mental Illness Stigma and discrimination. Schizophr Bull. 2004;30:481–91. 15631241 10.1093/oxfordjournals.schbul.a007096

[72] Hatzenbuehler ML. Structural stigma and the Health of Lesbian, Gay, and bisexual populations. Curr Dir Psychol Sci. 2014;23:127–32.

[73] Phelan JC, Link BG, Dovidio JF. Stigma and prejudice: one animal or two? Soc Sci Med. 2008;67:358–67. 18524444 10.1016/j.socscimed.2008.03.022 PMC4007574

[74] Scambler G. Health-related stigma. Sociol Health Illn. 2009;31:441–55. 19366430 10.1111/j.1467-9566.2009.01161.x

[75] Adkins-Jackson PB, Chantarat T, Bailey ZD, Ponce NA. Measuring structural racism: a Guide for epidemiologists and Other Health Researchers. Am J Epidemiol. 2022;191:539–47. 34564723 10.1093/aje/kwab239 PMC9077112

[76] Castle B, Wendel M, Kerr J, Brooms D, Rollins A. Public Health’s Approach to systemic racism: a systematic literature review. J Racial Ethnic Health Disparities. 2019;6:27–36. 10.1007/s40615-018-0494-x 29729001

[77] Bailey ZD, Krieger N, Agénor M, Graves J, Linos N, Bassett MT. Structural racism and health inequities in the USA: evidence and interventions. Lancet. 2017;389:1453–63. 28402827 10.1016/S0140-6736(17)30569-X

[78] Neblett EW. Racism measurement and influences, variations on scientific racism, and a vision. Soc Sci Med. 2023;316:115247. 36180279 10.1016/j.socscimed.2022.115247

[79] Homan P. Structural Sexism and Health in the United States: a New Perspective on Health Inequality and the gender system. Am Sociol Rev. 2019;84:486–516.

[80] Homan P, Brown TH, King B. Structural intersectionality as a New Direction for Health Disparities Research. J Health Soc Behav. 2021;62:350–70. 34355603 10.1177/00221465211032947 PMC8628816

[81] Kapadia D. Stigma, mental illness & ethnicity: time to centre racism and structural stigma. Sociol Health Illn. 2023;45:855–71. 36738120 10.1111/1467-9566.13615 PMC10946858

[82] Earnshaw VA, Watson RJ, Eaton LA, Brousseau NM, Laurenceau J-P, Fox AB. Integrating time into stigma and health research. Nat Rev Psychol. 2022;1:236–47. 35541283 10.1038/s44159-022-00034-2 PMC8900470

[83] Pachankis JE, Hatzenbuehler ML, Berg RC, Fernández-Dávila P, Mirandola M, Marcus U, et al. Anti-LGBT and anti-immigrant structural stigma: an Intersectional Analysis of Sexual Minority Men’s HIV Risk when migrating to or within Europe. JAIDS J Acquir Immune Defic Syndr. 2017;76:356–66. 28787329 10.1097/QAI.0000000000001519 PMC5659919

[84] Krieger N. Ecosocial Theory, Embodied Truths, and the People’s Health. 1st edition. Oxford University Press; 2021.

