# Supplementary material for: The use and operationalization of “structural stigma” in health-related research: A scoping review
Source: BMC Public Health. 2024 Dec 30;24:3614. doi: 10.1186/s12889-024-21171-8 (PMC11684274; doi:10.1186/s12889-024-21171-8)
Supplement: Supplementary file 2 — Supplementary Material 2 [file 12889_2024_21171_MOESM2_ESM.docx]

**Additional File 2 for *The use and operationalization of “structural stigma” in health-related research: A scoping review*.**

**“Other” sources of the structural stigma concept cited**

“Other” includes: Almeida et al. 2009; Altman et al. 2012; Beyond Blue 2015; Bourdieu 1977; Castro & Farmer 2005; Clough, Ireland, & March 2019; Corrigan & Watson 2002; Corrigan 2004; Corrigan 2005; Corrigan et al. 2005; Corrigan et al. 2010; Entire special issue of *Social Science & Medicine* on structural stigma; Feagin & Feagin 1986; Fox 2012; Frost 2011; Hannem 2012; Hansen et al. 2014; Hatzenbuehler & Keyes 2013; Hatzenbuehler, Nolen-Hoeksema, & Dovidio 2009; Hatzenbuehler & Pachankis 2016; Hatzenbuehler & Pachankis 2021; Hatzenbuehler, Slopen, & McLaughlin 2014; Heary, Hennessy, & Swords 2014; Herek 2007; Herek 2011; Herek 2016; Hill 1988; Hinshaw & Cicchetti 2000; Huggett et al. 2018; Klein et al. 2021; Lai et al. 2013; Lattanner et al. 2021; Lea, de Wit, & Reynolds 2014; Lee & Butts 2020; Leis & Rosenbloom 2009; Link & Hatzenbuehler 2016; Link & Phelan 2006; Link 2014; Link, Castille, & Stuber 2008; Livingston 2013; McCradden et al. 2019; McDowell et al. 2020; Merton 1948; Meyer 2003; Mitchell et al. 2021; Mora-Ríos & Bautista 2014; National Academies of Science Engineering Medicine 2020; Overton & Medina 2008; Pachankis et al. 2014; Pachankis et al. 2017; Parker & Aggleton 2003; Pharr et al. 2022; Phelan, Link, & Dovidio 2008; Philbin et al. 2021; Pincus 1999; Pryor & Reeder 2011; Pryor & Reeder 2015; Raghavan et al. 2008; Rüsch et al. 2005; Scambler 2006; Sheehan, Nieweglowski, & Corrigan 2017; Stevens et al. 2019; Tsai et al. 2019; Turan et al. 2017; Tyler & Slater 2018; Tyler 2018; Tyler 2020; van der Star, Bränström, & Pachankis 2021; Wilson 1990; Woo et al. 2017; and Yang et al. 2014

**“Other” theories or conceptual frameworks used**

*Other additional single frameworks*

“Other” can include: Adaptive Leadership Framework for Chronic Illness, Attribution theory, Avoidance coping, Cascade of care, Chronic stress response, Cognitive-affective-behavioral model of concealment, Community opportunity structure, Confucianism, Critical theory, (De)medicalization, Developing HIV Literacy framework, Dignity (attacks on dignity), Dissociation, Earnshaw & Chaudoir’s HIV stigma framework, Extra-legal policing, Filial piety, Fundamental cause theory, Gender disparities, Gender Minority Stress and Resilience Measure, Goffman’s social stigma, Healthism, Health-related deservingness, Herek's sexual stigma framework, Human rights, Illness metaphors, Interaction theory of emotion work, Intersectional stigma, Lifecourse, Microaggressions, Mitchell et al.'s (2021) "long arm of oppression," Modified labeling theory, Moral and structural distress, Motivational model of alcohol use, Need to belong, Network analysis, Performance theory, Political ecology of health and disease, Psychological mediation framework, Rejection sensitivity, Relational cultural theory, Reserve capacity model, Resilience, Risk containment, Rusbult’s (1980) investment model, Social capital, Social ecological model, Social genomic perspective, Social identity threat, Social model of disability, Social norms, Special interest groups, Stangl et al.’s (2019) Health Stigma and Discrimination Framework, Stereotype embodiment theory, Stigma complex, Stigma power, Stigma sensitivity, Structural ableism, Structural competency, Structural sexism, Suicidality theories, Symbolic interactionism, Syndemic theory, Taboo, White Hughto et al.’s (2015) multilevel model of stigma toward trans people

*Other multiple frameworks*

“Other multiple frameworks” can include: Abortion aversion and stigma complex; “A variety of thinkers who have contributed to theoretical approaches of political economy and moral economy”; Bourdieusian thought and ecosocial theory; Bourdieusian thought and Parker & Aggleton’s (2003) conceptualization of stigma; Confucianism and psychological mediation framework; Contact hypothesis and value self-confrontation theory; Ecosocial model, structural racism, stigma power, and Bourdieu's misrecognition; Goffman, Link & Phelan (2001), and structural vulnerability; Herek’s sexual stigma framework, symbolic violence, and social representations theory; Identity and agency in sociocultural groups and identity production in figured worlds; International Classification of Functioning, Disability and Health and Stevens et al.’s (2019) structural stigma model; Link & Phelan's discrimination model and the Maslach model of burnout; Parker & Aggleton’s (2003) conceptualization of stigma, structural vulnerability/violence, and everyday and symbolic violence; Post-structural philosophy and epistemic power; Relational turbulence theory and stigma management theory; Rhodes’s risk environment framework and complex adaptive systems theory; Stangl et al.’s (2019) Health Stigma and Discrimination Framework and human rights; Stigma management and "dangertalk” conceptual model; Stigma power and stigma machine; Structural vulnerability and What Matters Most
