# Supplementary material for: The use and operationalization of “structural stigma” in health-related research: A scoping review
Source: BMC Public Health. 2024 Dec 30;24:3614. doi: 10.1186/s12889-024-21171-8 (PMC11684274; doi:10.1186/s12889-024-21171-8)
Supplement: Supplementary file 3 — Supplementary Material 3 [file 12889_2024_21171_MOESM3_ESM.docx]

**Additional File 3 for *The use and operationalization of “structural stigma” in health-related research: A scoping review.***

**Additional Table 1.** Details of articles that quantitatively operationalize structural stigma (*N* = 102)

| **Author Date** | **Setting** | **Sample** | **Stigmatized status(es) of interest** | **Definition of structural stigma given** | **Source(s) of structural stigma concept** | **Additional theories/ conceptual frameworks used** | **Objective of study** | **Notes on how structural stigma was operationalized** |
| --- | --- | --- | --- | --- | --- | --- | --- | --- |
| Engaged with only laws and government-level policies (*n* = 31) | | | | | | | | |
| Azagba et al. 2022 | United States | Data on treatment service utilization were drawn from the Treatment Episode Data Set from 1992 to 2017 | Substance use (Alcohol use) | No | N/A | N/A | To examine the effects of repealing alcohol exclusion laws on treatment admissions for alcohol use disorder | Alcohol exclusion laws are framed as a mechanism of structural stigmatization; the repeal of alcohol exclusion laws at the state level were operationalized as the main independent variable |
| Babbs et al. 2023 | United States | 8,322 sexual and gender minority undergraduate and graduate students | Sexual minority status  Gender minority status | No | N/A | N/A | To demonstrate how health care denial policies contribute to the effects of structural stigma by increasing mental distress among sexual and gender minority university students | Health care denial policies (i.e., policies that allow health care providers to deny any health care based on a patient’s actual or perceived sexual orientation or gender identity, such as under the guise of religious freedom) explicitly stated to be a form of structural stigma; participants were shown the description of a health care denial policy and were asked to respond to three items related to mental distress and anticipated stigma |
| Brooks et al. 2022 | United States | 253 sexual minority adults recruited from a larger study examining the psychological risk and protective factors of sexual and gender minority health | Sexual minority status | No | N/A | Minority stress  Other (Psychological mediation framework) | To examine the association between anticipated stigma in response to the 2016 United States presidential election and symptoms of anxiety and depression, and the potential mediating role of self-compassion, hopelessness, and social support | Anticipated structural stigma score from *de novo* 6-item participant response scale |
| Campbell & Mena 2021 | United States | 212 college and university websites | Sexual minority status  Gender minority status | Yes | Hatzenbuehler & Link 2014 | Minority stress | To examine whether the friendliness of college counseling center websites to LGBTQ+ students varied by state-level structural policies on hate crimes, employment nondiscrimination, and religious exemption | Framing for the construction of three state-level structural policies variables around state hate-crime protection, employment nondiscrimination, and religious exemption laws |
| Cascalheira et al. 2022 | United States | All facilities that participated in the National Survey of Substance [Use] Treatment Services and the National Mental Health Services Survey from 2010 to 2020 in the 50 states in the United States | Sexual minority status  Gender minority status | Yes | Hatzenbuehler & Link 2014 | N/A | To examine structural stigma and government funding as two structural determinants affecting the availability of programming tailored for sexual and gender minority people across behavioral health facilities in the United States | Assessed via the Movement Advancement Project’s state-level index for sexual and gender minority civil rights |
| Conley & Baum 2022 | United States | 200 bills | Mental health | Yes | Hatzenbuehler & Link 2014 | N/A | To identify forms of structural stigma present in state mental health legislation as well as any associations with legislative factors | Used as a concept in quantitative content analysis wherein bills were coded as containing language indicating structural stigma and/or as having potential stigmatizing effects |
| Conley 2021 | United States | 200 bills | Mental health | Yes | Hatzenbuehler & Link 2014 | N/A | To examine forms of structural stigma in recent state mental health bills and any factors that influence bill outcomes | Used as a concept in quantitative content analysis wherein bills were coded as containing language indicating structural stigma and/or as having potential stigmatizing effects |
| Corrigan, Watson, Heyrman et al. 2005 | United States | 968 bills | Mental health | No | N/A | N/A | To examine trends in structural stigma in state legislation related to people with mental illness | Application of codebook of themes related to structural stigma; however, almost exclusively referred to as "structural discrimination" throughout the article |
| Doyle & Molix 2015 | United States | Two studies; one with a sample of 214 sexual minority people of any gender and the other with a sample of 94 gay men and lesbian women | Sexual minority status | Yes | Other (Feagin & Feagin 1986) | Minority stress | To examine the effects of both perceived discrimination and structural stigma on social relationship functioning | Used a state-level public policy index using data from the Human Rights Campaign, including public policy relevant to sexual minority rights such as hospital visitation rights, housing nondiscrimination, second parent adoption rights, joint adoption rights, employment nondiscrimination, marital equality, marital prohibition, hate crime laws, school antibullying policy, and school nondiscrimination |
| Drabble et al. 2021 | United States | 14,395 cisgender women (413 sexual minority and 13,982 heterosexual) and 11,115 cisgender men (421 sexual minority and 10,694 heterosexual) | Sexual minority status | Yes | Hatzenbuehler & Link 2014 | Minority stress | To explore whether structural stigma moderates the relationship between sexual minority identity and heavy drinking, alcohol problems, and marijuana use among cisgender men and women | Operationalized as "state policy environment" using an index of ten policies relevant to sexual minorities, which were adapted from the Movement Advancement Project: (1) legalized marriage for same-sex couples; (2) nondiscrimination protections in adoption or foster parenting for same-sex couples; (3) prohibition against discrimination by employers (both private and public/government); (4) housing nondiscrimination laws; (5) laws prohibiting discrimination in public accommodations; and (6) hate crimes laws that cover sexual minorities; (7) bans that explicitly prohibit same-sex marriage, (8) policies that allow denial of adoption and/or foster care by same-sex couples, (9) state bans on cities/counties passing non-discrimination laws, and (10) religious exemption laws permitting discrimination based on religious or moral grounds |
| Everett et al. 2022 | United States | 7913 total singleton births (274 to bisexual women, 53 to lesbian women) | Sexual minority status | Yes | Hatzenbuehler 2016 | N/A | To examine the association between social policies that confer legal protections for sexual minority people and birth outcomes among sexual minority women | Used "lesbian, gay, and bisexual policy protections," which authors indicate as reflecting efforts to reduce the level of structural stigma |
| Flentje et al. 2021 | United States | 5,299 sexual and gender minority people, including 1,902 gender minority individuals | Sexual minority status  Gender minority status | Yes | Hatzenbuehler 2016 | Minority stress | To examine which components of minority stress and structural stigma have the strongest relationships with physical health among sexual and gender minority people | Used state-level index scores from the Movement Advancement Project that reflect the number of sexual and gender minority-supportive and -harmful laws and policies within the following domains: marriage and relationship recognition, adoption and parenting, protections from discrimination, safe schools, health and safety, and the option for transgender people to obtain identity documents that accurately reflect their gender |
| Górska, Bilewicz, and Winiewski 2017 | Multi-country (Europe) | 1365 adults that were all either lesbian women, gay men, bisexual women, or bisexual men | Sexual minority status | No | Hatzenbuehler 2014 | Sexual stigma conceptual framework | To investigate the effects that heterosexist legal regulations exert on collective action of lesbian, gay, and bisexual people | Explicitly linked to "institutional sexual stigma,” which is operationalized as: 1) Rainbow Europe Index 2015—an assessment provided by the European division of International Lesbian, Gay, Bisexual, Trans and Intersex Association and combines various areas of LGBTQI individuals’ legal recognition, such as protection from discrimination and hate crime, relational rights, or freedom of expression; and 2) the time (in years) elapsed since the institutionalization of same-sex civil partnerships was introduced in a given country |
| Górska, van Zomeren, and Bilewicz 2017 | Multi-country (Europe) | 27,409 people who did not identify themselves as lesbian, gay, bisexual, or transgender | Sexual minority status | No | Hatzenbuehler 2014 | Sexual stigma conceptual framework | To test whether the prevalence of intergroup contact with lesbian, gay, and bisexual individuals explains the relationship between more progressive lesbian, gay, and bisexual rights and sexual prejudice | Used as a concept under the umbrella term of "institutional structural stigma," which was then operationalized as a variable called "LGB-related legislation"; two measures were used: 1) the Rainbow Europe Index published by the International Lesbian, Gay, Bisexual, Trans and Intersex Association and covers individuals’ legal recognition (see row above for details; and 2) time in years since same-sex couples legal recognition – either in the form of marriage or civil union |
| Greene et al. 2021 | United States | 435,921 women and 339,660 men (assumed cisgender) | Sexual minority status | Yes | Hatzenbuehler, Phelan & Link 2013 | Minority stress | To explore how state-level alcohol policy environments interact with state-level nondiscrimination statutes that include protections on the basis of sexual orientation (i.e., inclusive nondiscrimination statutes) to contribute to disparities in binge drinking between lesbian, gay, and bisexual and heterosexual adults | States' inclusive nondiscrimination statues are explicitly designated as representing a reduction in structural stigma; operationalized as a binary indicator of whether a state’s nondiscrimination laws across employment, housing, and public accommodations explicitly include sexual orientation |
| Hollinsaid, Price, & Hatzenbuehler 2022 | United States | 50 United States states and Washington, DC | Gender minority status | Yes | Hatzenbuehler 2016  Hatzenbuehler 2017 (JCCAP) | N/A | To examine whether transgender-specific adolescent mental health provider availability varied by states’ transgender-specific policy climate | Explicitly linked to “state policy climate,” which was assessed via data from the Movement Advancement Project on the presence or absence of 33 transgender-specific state laws/policies in six domains (i.e., parental/relationship recognition, nondiscrimination, education, healthcare, criminal justice, and identity documentation) |
| Kcomt et al. 2023 | United States | 33,197 individuals 14 years of age or older, including 49 who reported being transgender at each survey wave and 377 who reported being transgender at one or more wave | Gender minority status | Yes | Hatzenbuehler 2016  White Hughto et al. 2015 | Social ecological model | To examine individual-, interpersonal-, community-, and policy-level associations with nicotine/tobacco use among gender-varying and gender-stable individuals | Explicitly linked to “gender minority policy protections,” which were assessed via data from the Movement Advancement Project; these data were then used to created a measure that reflected the change in state-level gender minority policy protection from 2015 to 2017 |
| Layland et al. 2023 | Multi-country (Europe) | 111,498 bisexual, gay, and lesbian people at least 15 years of age | Sexual minority status | Yes | Hatzenbuehler 2016 | Minority stress  Other (The closet) | To examine associations between structural stigma and identity-related developmental milestones (i.e., age of awareness as a sexual minority person, age of coming out, and duration of the closet) | Used an index of lesbian, gay, and bisexual equal rights to reflect country-level structural stigma, created using data form the International Lesiba, Gay, Bisexual, Trans, and Intersex Association Europe |
| Mann, O'Leary, & Blackaby 2022 | Multi-country (Europe) | 137,075 individuals, including 1742 sexual minority individuals | Sexual minority status | No | N/A | Other (Special interest groups) | To analyze the impact of same-sex relationship recognition policies on the political trust of sexual minorities | Used the presence or absence of same-sex relationship recognition policies, which are explicitly linked to being a reduction of structural stigma |
| Nelson, Wardecker, & Ross 2023 | United States | 11,104 adults aged 50 and older, including 10,032 sexual minority older adults and 1,072 transgender older adults | Sexual minority status  Gender minority status  Age | Yes | Hatzenbuehler & Link 2014  Hatzenbuehler 2014 | Minority stress | To examine the associations between state-level policies and the health of lesbian, gay, bisexual, and transgender older adults | Rationale and framing for quantitative analysis; operationalized as a State-Level LGBT-Related Policy Tally using data from the Movement Advancement Project |
| Norcini Pala et al. 2017 | Italy | 451 gay and bisexual men | Sexual minority status | Yes | Other (Herek 2011) | Minority stress | To explore the dimensionality, internal reliability, and convergent validity of the Minority Stress Scale, a comprehensive instrument designed to assess the manifestations of sexual orientation stigma | Part of conceptual framework for a scale, and pre-defined as a factor in scale development |
| Pachankis & Bränström 2019 | Multi-country (Europe) | Modeling study that used data from 85,582 people who identified as lesbian, gay, or bisexual | Sexual minority status | Yes | Hatzenbuehler 2016 | N/A | To extrapolate the proportion of the global sexual minority population who conceals its sexual orientation | Operationalized as "country-level structural stigma" using an index summing across each of 197 countries’ distinct forms of legal and policy discrimination and protections spanning six domains (i.e., unequal age of consent for same-sex sexual activity, asylum provisions for sexual minorities, protections against bias-motivated violence, legal protections against discrimination, same-sex partnership and parenting recognitions, freedom of assembly) using data from the International Lesbian, Gay, Bisexual, Trans and Intersex Association |
| Passell et al. 2021 | Multi-continent | 5929 individuals | Sexual minority status | Yes | Hatzenbuehler 2016 | N/A | To examine the relationship between sexual minority status, structural stigma, and generalized anxiety disorder symptoms | Operationalized as "structural stigma" in analysis (specifically marriage inequality and criminalization of same-sex sexual acts) using data from the International Lesbian, Gay, Bisexual, Trans and Intersex Association |
| Pharr et al. 2022a | United States | 1,033 people who identified as a sexual and gender minority | Gender minority status | Only examples | Hatzenbuehler & Link 2014  Hatzenbuehler 2017 (Handbook) | Minority stress | To: 1) determine if familiarity with state-level transgender sports bans was associated with suicidality among sexual and gender minority adults, and 2) determine if interpersonal stigma and/or individual stigma mediated this association | Operationalized as participants’ awareness of US state-level transgender sports bans |
| Pharr et al. 2022b | United States | 1,033 people who identified as a sexual and gender minority | Gender minority status | Only examples | Hatzenbuehler 2017 (Handbook)  van der Star, Bränström, & Pachankis 2021 | Other (Resilience) | To understand if the relationship between familiarity with transgender sports bans and suicidality was moderated by individual or community resilience | Operationalized as participants’ awareness of US state-level transgender sports bans |
| Raifman et al. 2023 | United States | 2012−2019 AIDSVu data on PrEP prescriptions in each United States state and year | Sexual minority status | Yes | Hatzenbuehler 2014 | Fundamental cause theory | To evaluate whether lower structural stigma, reflected by earlier year of state same-sex marriage legalization, was associated with increased male PrEP prescriptions and male PrEP-to-need ratio | Used relative timing of state same-sex marriage legalization as a reflection of lower to higher structural stigma |
| Rucco et al. 2023 | Italy | 299 Italian bisexual+ people, including 39 men and 260 women | Sexual minority status | Yes | Link & Phelan 2001 | Minority stress | To explore whether there was a worsening in mental health among bisexual+ people before and after the rejection of the Zan Bill (formally titled "Measures to prevent and combat discrimination and violence on grounds of sex, gender, sexual orientation, gender identity, and disability") | The rejection of the Zan Bill (which would have provided additional formal legal protections for LGBTQ+ people) was seen as a form of structural stigma |
| Skinner et al. 2023 | United States | 3,296 gay, bisexual, and other men who have sex with men | Sexual minority status | Yes | Hatzenbuehler, Phelan & Link 2013 | N/A | To assess the relationships between same-sex marriage laws, provider-patient communication about sex, and awareness and use of PrEP among gay, bisexual, and other men who have sex with men | Structural stigma at the state level was assessed via the presence or absence of same-sex marriage laws in participants’ state of residence as of January 1, 2013 |
| Stojanovski et al. 2022 | Multi-country (Europe) | 98,600 men who have sex with men | Sexual minority status | No | N/A | N/A | To examine how stigmatizing policies interact with downstream anxiety/depression and sexual behaviors to structurally pattern HIV disparities among European Men who have sex with men | Operationalized as country-level stigmatizing policies via the Rainbow Index developed by the International Lesbian, Gay, Bisexual, Transgender, and Intersex Association of Europe |
| Tran et al. 2022 | United States | 4335 transgender individuals, including 1881 transgender women, 1464 transgender men, and 990 gender nonconforming people | Gender minority status | Yes | Hatzenbuehler, Phelan, & Link 2013 | N/A | To assess 1) the association between structural stigma and four measures of individual health care access among transgender people in the United States, and 2) the extent to which structural stigma explains state-level variability | Operationalized as an exposure variable in analysis using the HRC State Equality Index; specifically, used the State Equality Index as a proxy for anti-transgender stigma as it is a global measure of discrimination against sexual and gender minority populations; the index covers the six domains of: parental rights (e.g. second parent adoption), hate crimes and criminal justice (e.g. sodomy laws, HIV criminalization), nondiscrimination protections (e.g. nondiscrimination in employment, housing, public accommodations, and education), religious exemptions (e.g. state religious freedom restoration acts), protections for sexual and gender minority youth (e.g. prohibition of conversion therapy), and health care protections (e.g. bans on insurance exclusion for gender-affirming health care) |
| Zelin et al. 2020 | Multi-continent | 19 OutLists at medical institutions | Sexual minority status  Gender minority status | No | N/A | N/A | To characterize OutLists (i.e., online, opt-in lists of sexual and gender minority individuals) at medical institutions | Measured using the Human Rights Campaign State Equality Index values of the home states of institutions |
| *Engaged with governmental laws and policies and additional structural factor* (*n* = 1) | | | | | | | | |
| Askew et al. 2023 | United States | 942 cisgender sexual minority individuals ages 18-30 | Race/ethnicity  Sexual minority status | Yes | Hatzenbuehler & Link 2014 | Intersectionality  Minority stress | To determine how relevant forms of structural stigma were related to eating pathology, misuse of appearance and/or performance-enhancing drug(s), and dysmorphic concern among a racially diverse sample of sexual minority men and women | Assessed two forms of structural stigma: 1) structural racism, which was assessed through the use of the Racial Equality Index developed by the National Equity Atlas and has two components of a) an inclusion score that evaluates the magnitude of disparities for a series of nine equity indicators that assess economic vitality (wages, unemployment, and poverty), readiness (educational attainment, disconnected youth, and school poverty), and connectedness (air pollution exposure, commute time, housing burden), and b) a prosperity score that indicates how well that geographic region is performing based on the same nine equity indicators; and 2) structural sexual minority stigma, which was assessed through the use of the sexual orientation state policy tally scores from the Movement Advancement Project |
| Engaged with laws and government-level policies as well as sociocultural attitudes and norms (*n* = 18) | | | | | | | | |
| Bränström & Pachankis 2021 | Multi-country (Europe) | 6771 transgender adults from the European Union Lesbian, Gay, Bisexual, and Transgender Survey in 2012 | Gender minority status | Yes | Hatzenbuehler 2016 | Minority stress | To examine the association between structural stigma and transgender people’s life satisfaction | Used a measure of country-level structural stigma related to transgender identity composed of 1) an index score reflecting discriminatory and protective laws and policies concerning transgender people, and 2) a measure of social attitudes toward transgender people (i.e., to what degree respondents would feel comfortable with a transgender person in the highest political position in their country) |
| Bränström & Pachankis 2023 | Multi-country (Europe) | Two surveys of 82,668 and 96,576 sexual and gender minority individuals ages 18 years and older with no history of migration | Sexual minority status | Yes | Hatzenbuehler 2016 | Minority stress  Other (Fundamental cause theory) | To: 1) investigate the association between changes in country-level structural stigma related to sexual minority status during the past decade with sexual minority individuals’ life satisfaction across 28 European countries, and 2) explore potential subgroup differences in this association | Measured using  an index of laws and policies concerning sexual minority individuals collected by the International Lesbian, Gay, Bisexual, Trans and Intersex Association in Europe and a measure of social attitudes across Europe from the European Social Survey, which asked respondents in each country whether “gays and lesbians should be free to live their own life as they wish” |
| Bränström, Fellman & Pachankis 2022 | Multi-country (Europe) | 86,308 sexual minority adults from the European Union Lesbian, Gay, Bisexual, and Transgender Survey in 2012 | Sexual minority status | Yes | Hatzenbuehler 2016 | Intersectionality  Minority stress  Other (Fundamental cause theory) | To examine the relationship between country-level structural stigma and victimization, how that relationship may differ by gender conformity and socioeconomic status, and intersectionally, how gender moderates these associations | Used a country-level index score that aggregated national laws, policies, and population attitudes negatively affecting sexual minority individuals; laws and policies were from the International Lesbian, Gay, Bisexual, Trans and Intersex Association in Europe, and population attitudes were from the European Social Survey, which asked respondents in each country whether “gays and lesbians should be free to live their own life as they wish” |
| Chang et al. 2021 | Multi-continent | The 56 countries in the analysis represent 63.1% of the global older population aged 60 years and older, representing all six WHO regions; part of structural ageism variable uses data from 82,249 from the World Values Survey | Age | Only examples | Hatzenbuehler, Phelan & Link 2013  Hatzenbuehler 2016  Pachankis et al. 2015 | N/A | To determine the association between country-level structural ageism and prevalence of violence against older persons | Operationalized as a continuous structural ageism variable composed of two parts: (1) discriminatory national policies related to older persons' economic, social, civil and political rights, based on the four core components of human rights protection in Madrid International Plan of Action on Aging and (2) prejudicial social norms against older persons, measured by negative attitudes toward older persons in 56 national polls in the World Values Survey aggregated to country-level |
| Falck & Bränström 2023 | Multi-country (Europe) | 6,771 transgender individuals | Gender minority status | Yes | Hatzenbuehler & Link 2014 | Minority stress | To explore the association between structural stigma and access to gender affirming care, gender identity disclosure in health care, and experiences of discrimination in health care among transgender people | Used an index combining laws and policies data from the International Lesbian, Gay, Bisexual, Trans and Intersex Association in Europe and population attitudes toward transgender people from the Eurobarometer, which asked how comfortable respondents would feel about “having a transgender or transsexual person in the highest elected political position in their country” |
| Hatzenbuehler et al. 2022 | United States | 11,534 youth from 21 study sites | Gender  Race/ethnicity | Yes | Hatzenbuehler & Link 2014 | N/A | To determine whether being raised in a stigmatizing context influences neurodevelopment (i.e., hippocampal volume and amygdala reactivity to threat) in children | Used publicly available data sources to create proxy measures of social climate for three groups of interest (i.e., women, Black people, and Latinx people); structural stigma related to gender was measured via 18 items on aggregated implicit and explicit attitudes as well as previous state-level composite indicators of women’s social status including: economic (e.g., ratio of men’s to women’s earnings), political (e.g., women’s representation in elected office), social and economic autonomy (e.g., women’s business ownership); and reproductive (e.g., percentage of women who live in a county without an abortion provider) factors; structural stigma related to race (i.e., Black race) was measured via 31 items on aggregated attitudes related to race and racial prejudice including general attitudes towards Black individuals, the impact of discrimination on the lives of Black people, the existence of racial prejudice, and endorsement of racial stereotypes; and structural stigma related to Latinx ethnicity was measured via three indicators: a feelings thermometer of explicit attitudes of immigrants, a composite index of state-level policies related to immigration, and a feelings thermometer of explicit attitudes of Hispanics |
| Hatzenbuehler, Bränström, & Pachankis 2018 | Sweden | 23,248 individuals, including 565 who identify as lesbian, gay, or bisexual | Sexual minority status | Yes | Hatzenbuehler & Link 2014 | Minority stress | To 1) explore associations between reductions in structural stigma during the past decade in Sweden and sexual orientation-based mental health disparities, and 2) identify potential mechanisms that might explain this relationship (i.e., perceived discrimination, victimization/threats of violence, and expectations of victimization) | Created a standardized index score that combined data on Swedish laws and policies toward sexual minorities (e.g., same-sex marriage legislation, acceptance of fertility treatment for lesbian couples) and social attitudes (“gays and lesbians should be free to live their own life as they wish”) |
| Miedema et al. 2019 | Multi-country (Asia-Pacific) | 526 sexual minority men | Sexual minority status | Yes | Hatzenbuehler 2017 (Handbook) | Minority stress | To conduct a multilevel empirical test of individual- and cluster-level indicators of sexual stigma on depressive symptoms among sexual minority men in four Asia-Pacific countries | Developed a measure of socio-structural stigma, which was measured via four indicators: 1 and 2) cluster-level injunctive beliefs around homosexuality which included a) the proportion of all men in the cluster who believed it was shameful to have a homosexual son, and b) the proportion of all men in the cluster who believed that there should not be laws or policies to protect homosexual people; 3) a single measure of empirical norms around homosexuality which was the proportion of men who reported perpetration of male-on-male rape or sexual assault, and 4) a dichotomous measure that indicated the presence of any anti-homosexuality laws or policies in that country based on the International Lesbian, Gay, Bisexual, Trans and Intersex Association’s 2017 report of global laws around homosexuality |
| Miller et al. 2018 | United States | 1793 youth | HIV | Yes | Hatzenbuehler & Link 2014 | Other (Community opportunity structure) | To examine the influence of concentrated disadvantage, HIV stigma, and sexual and gender minority stigma on engagement in HIV risky sexual partnerships and whether youth’s participation in opportunity structures, anticipation of HIV stigma, and perceptions of their community as youth-supportive settings mediated structural effects | Operationalized as structural stigma of sexual and gender minorities, which was a composite measure made by: 1) counting the absence of state laws in six categories pertinent to the protection of people on the basis of their sexual orientation and/or identification as transgender: (a) hate crime legislation; (b) adoption; (c) employment discrimination; (d) marriage discrimination; (e) prohibitions on including normalizing discussions of same-sex attractions and behaviors in youth sexuality education; and (f) requirements that sexuality education promote heterosexual marriage; and 2) structural HIV stigma, which was a social attitudes scale used at individual, ZIP code, and state levels (e.g., “In my community, people with HIV/AIDS are treated like outcasts”) |
| Pachankis & Bränström 2018 | Multi-country (Europe) | 85,582 sexual minority adults | Sexual minority status | Yes | Hatzenbuehler 2016 | Minority stress | To examine: 1) the association between structural stigma and sexual minority individuals' life satisfaction across 28 countries, 2) identity concealment as a potential mechanism of this association, and 3) in high-stigma countries, the potential for concealment to protect sexual minorities from discrimination and victimization, and therefore even poorer life satisfaction than they would otherwise experience in those countries | Used a measure of structural stigma related to sexual orientation combining 1) an index of laws and policies toward sexual minorities collected by the International Lesbian, Gay, Bisexual, Trans and Intersex Association in Europe across six domains of discrimination and protections (i.e., unequal age of consent for same-sex sexual acts, asylum provisions for sexual minorities, protections against bias-motivated violence, legal protections against discrimination, same-sex partnership and parenting recognitions, freedom of assembly); and 2) a measure of social attitudes from the European Social Survey, which asked respondents in each country whether “gays and lesbians should be free to live their own life as they wish” |
| Pachankis et al. 2015 | Multi-country (Europe) | 174,209 men who have sex with men | Sexual minority status | Only examples | Hatzenbuehler & Link 2014 | N/A | To examine the impact and underlying mechanisms of country-level stigma on odds of diagnosed HIV, sexual opportunities, and experience of HIV-prevention services, needs and behaviors | Operationalized as "country-level stigma," which was assessed using a combination of: 1) national legislation as compiled by the International Lesbian, Gay, Bisexual, Trans, and Intersex Association Europe Rainbow Index covering 10 supportive legislative policies (e.g. same-sex marriage, employment nondiscrimination legislation) and four discriminatory practices and legislative policies (e.g. violation of freedom of assembly); and 2) country-level attitudes toward sexual minorities using data from the European Values Survey by calculating the proportion of respondents in each country who thought homosexuality could be justified; agreed that same-sex couples should be able to adopt children; and did not indicate not wanting to have homosexual neighbors |
| Pachankis et al. 2021 | Multi-continent | 123,428 sexual minority men | Sexual minority status | Yes | Hatzenbuehler & Link 2014 | Minority stress | To examine 1) whether changes in structural stigma, such as when a stigmatized person moves to a lower stigma context, affects mental health, and 2) the mechanisms underlying this association | Operationalized as country-level structural stigma, which was measured via: 1) 15 laws and policies related to sexual orientation (in the three domains of legal discrimination and criminalization; recognition; and protection) compiled from the International Lesbian, Gay, Bisexual, Trans and Intersex Association, and 2) country-level attitudes toward sexual minority people from the Global Acceptance Index, which provides a country-aggregated score based on responses to 67 questions about acceptance of LGBT people contained in 11 international surveys (e.g., Gallup World Poll, World Values Survey) |
| Pachankis, Hatzenbuehler, & Starks 2014 | United States | 119 young sexual minority men | Sexual minority status | Yes | Link & Phelan 2001 | Other (Rejection sensitivity) | To examine whether stigma at the individual level, namely gay-related rejection sensitivity, interacts with structural stigma to predict substance use among young sexual minority men | Accounted for both current and past exposures to structural stigma; operationalized as both policies and aggregate social attitudes: 1) the presence of five state policies affecting sexual minority individuals in the state of residence from the Human Rights Campaign; and 2) eight questions regarding attitudes toward policies affecting gays and lesbians in that state from the Roper Center's iPol archive |
| Pachankis, Hatzenbuehler, Berg, et al. 2017 | Multi-country (Europe) | 23,371 men who have sex with men who migrated to Europe | Immigrant status  Sexual minority status | Yes | N/A | Intersectionality | To investigate structural determinants of HIV risk in a large, geographically-diverse sample of men who have sex with men who have migrated to Europe | Accounted for both sending and receiving countries’ structural stigma; used 1) anti-gay structural stigma as assessed by national legislation toward sexual minorities (derived from the International Lesbian, Gay, Bisexual, Trans, and Intersex Association’s Rainbow Map and 2) anti-immigrant structural stigma as assessed by five immigrant-related questions on the European Values Survey concerning immigrants and crime, jobs, financial strain, national threat, and cultural erosion |
| Pachankis, Hatzenbuehler, Mirandola et al. 2017 | Multi-country (Europe) | 174,209 men who have sex with men | Sexual minority status | Yes | Hatzenbuehler & Link 2014 | N/A | To examine country-level structural stigma as a key correlate of the geographic variation in men who have sex with men's sexual attraction, behavior, and identity, and concordance across these factors | Assessed country-level stigma using a combination of national legislation (from the International Lesbian, Gay, Bisexual, Trans, and Intersexual Association-Europe Rainbow Index) and general population attitudes toward sexual minorities from the European Values Survey (proportion of respondents in each country who: a) thought homosexuality could be justified; b) agreed that same-sex couples should be able to adopt children; and (3) did not indicate not wanting to have homosexual neighbors |
| Ünsal, Demetrovics, & Reinhardt 2022 | Multi-country (Europe) | 117,760 sexual and gender minority adults, including 62,939 sexual minority men, 38,976 sexual minority women, and 15,845 gender minority adults | Sexual minority status  Gender minority status | Yes | Hatzenbuehler & Link 2014 | Intersectionality  Minority stress | To examine the mediator roles of identity disclosure and victimization in the association of community participation with depression among sexual and gender minority individuals, as well as the moderator role of structural stigma in these associations | Used the International Lesbian, Gay, Bisexual, Trans, and Intersexual Association’s Rainbow Index for laws and policies as well as Eurobarometer for social attitudes (respondents rated how comfortable they would feel about having “a gay, lesbian or bisexual person” and “a transgender person” in the highest elected political position in their country) |
| van der Star, Bränström, & Pachankis 2021 | Sweden | 247 sexual minority men who had migrated to Sweden | Sexual minority status | Yes | N/A | Minority stress | To explore the association between structural stigma exposure, minority stress reactions and mental health, as a function of length of exposure to structural stigma among self-identified sexual minority men who have changed structural stigma environments | Structural stigma in participants’ country of origin was based on a country-level index of population attitudes (using data from the Global Acceptance Index) and the presence of discriminatory laws and policies (using data from the International Lesbian, Gay, Bisexual, Trans and Intersex Association; means of this composite score were centered around the reference score for Sweden |
| van der Star, Pachankis, & Bränström 2021 | Multi-country (Europe) | 55,263 sexual minority individuals | Sexual minority status | Yes | Hatzenbuehler 2016 | Other (Lifecourse) | To explore whether and how structural stigma might be associated with sexual minorities’ school-based and adulthood experiences of victimization and adulthood life satisfaction | Operationalized using a measure regarding population attitudes toward homosexuality (i.e., from the European Values Survey) and an index of discriminatory legislation and policies (i.e., the Europe Rainbow Index created by the International Lesbian, Gay, Bisexual, Trans and Intersex Association Europe) |
| *Engaged with laws and government-level policies; sociocultural attitudes and norms; and additional structural factor(s) (n = 14)* | | | | | | | | |
| Almazan et al. 2021 | United States | 447 federally qualified health centers | Sexual minority status  Gender minority status | Yes | Hatzenbuehler & Link 2014 | N/A | To examine the relationship between city-level structural stigma pertaining to sexual orientation and gender identity (SOGI) and completeness of patient SOGI data collection at US federally qualified health centers (FQHCs) | Used city-level structural stigma index scores from Human Rights Campaign’s Municipal Equality Index |
| Blosnich et al. 2016 | United States | 1640 people who were Veterans Administration patients, were diagnosed with gender identity disorder, had at least 1 visit in 2013, and lived in a zip code with a Municipality Equality Index Score | Gender minority status  Veteran status | Yes | Hatzenbuehler & Link 2014 | N/A | To examine whether indicators of community- and state-level lesbian, gay, bisexual, and transgender equality are associated with transgender veterans’ mental health | Used 1) city-level and state-level index scores from the Human Rights Campaign's Municipality Equality Index and ii) two dichotomous variables for employment nondiscrimination and hate crimes laws that include transgender status or gender identity |
| Charlton et al. 2019 | United States | 6,581 cisgender women from the Growing Up Today Study | Sexual minority status | Yes | Hatzenbuehler & Link 2014 | N/A | To examine whether exposure to structural stigma is associated with sexually transmitted infections and teen pregnancy in sexual minority female adolescents | Used a previously-validated structural stigma scale composed of four state-level items (i.e., density of same-sex partner households, proportion of high schools with gay-straight alliances, a composite variable of five state-level protective polices related to sexual orientation such as employment non-discrimination policies, and public opinion toward sexual minorities) with one item added relevant to reproductive health (i.e., percent of women without an abortion provider in their county) |
| Gordon et al. 2023 | United States | 11,622 youth ages 9-13 | Sexual minority status | Yes | Hatzenbuehler & Link 2014 | Minority stress | To characterize mental health disparities of sexual minority youth in a cohort study, determine the contribution of interpersonal sexual minority discrimination experiences and of structural sexual minority stigma to youth mental health, and test components of the minority stress theory by evaluating the extent to which interpersonal sexual minority discrimination may explain mental health disparities among sexual minority youth | Used the following 8 indicators of structural stigma: 1) the presence or absence of 33 protective and discriminatory state laws and policies related to sexual orientation; 2) explicit attitudes toward acceptance of homosexuality and legality of same-sex marriage; 3) explicit policy-specific support of rights for sexual minority people and same-sex couples based on public opinion polls; 4) implicit attitudes toward gay men and lesbian women; 5) proportion of openly sexual minority elected government officials; 6) proportion of public high schools with gay-straight alliances (or gender-sexuality alliances); 7) estimated percentage of sexual minority adults in each state; and 8) estimated density of same-sex couples in each state |
| Hatzenbuehler & McLaughlin 2014 | United States | 74 lesbian, gay, and bisexual young adults from 24 states | Sexual minority status | Yes | Link & Phelan 2001  Corrigan, Watson, Heyrman et al. 2005 | Minority stress | To examine whether growing up in highly stigmatizing environments similarly shapes stigmatized individuals’ physiological responses to identity-related stress | Used a previously-validated state-level index score of structural stigma comprised of four items: 1) density of same-sex partner households, 2) proportion of gay-straight alliances per public high school, 3) composite score of five state-level policies related to sexual orientation (i.e., absence of constitutional amendments banning same-sex marriage, employment nondiscrimination policies, hate crime policies that include sexual orientation as a protected class, nondiscrimination policy that extended to sexual minority students and/or a statute banning bullying based explicitly on sexual orientation, and statutes that do not explicitly restrict gay and lesbian couples from adoption), and 4) public opinion toward sexual minority people in each state; participants were assigned the score of their state of longest residence during the ages of 10-18 |
| Hatzenbuehler et al. 2014 | United States | 16,882 youth | Sexual minority status | No | Link & Phelan 2001 | Minority stress | To examine whether exposure to structural stigma predicts cigarette smoking in sexual minority youth | Developed a state-level index score of the social environment surrounding sexual minority youth comprised of four items: 1) density of same-sex partner households, 2) proportion of gay-straight alliances per public high school, 3) composite score of five state-level policies related to sexual orientation (i.e., absence of constitutional amendments banning same-sex marriage, employment nondiscrimination policies, hate crime policies that include sexual orientation as a protected class, nondiscrimination policy that extended to sexual minority students and/or a statute banning bullying based explicitly on sexual orientation, and statutes that do not explicitly restrict gay and lesbian couples from adoption), and 4) public opinion toward sexual minority people in each state |
| Hatzenbuehler et al. 2015 | United States | 12,723 youth, including 2,789 sexual minority youth | Sexual minority status | Yes | Hatzenbuehler & Link 2014 | N/A | To determine whether sexual orientation disparities in illicit drug use are potentiated in states that are characterized by high levels of stigma surrounding sexual minority people | Used a state-level index score structural stigma comprised of four items: 1) density of same-sex partner households, 2) proportion of gay-straight alliances per public high school, 3) composite score of five state-level policies related to sexual orientation (i.e., absence of constitutional amendments banning same-sex marriage, employment nondiscrimination policies, hate crime policies that include sexual orientation as a protected class, nondiscrimination policy that extended to sexual minority students and/or a statute banning bullying based explicitly on sexual orientation, and statutes that do not explicitly restrict gay and lesbian couples from adoption), and 4) public opinion toward sexual minority people in each state |
| Jackson et al. 2023 | United States | 2,561 Black and Latinx LGBTQ+ adolescents ages 13-17 | Race/ethnicity  Sexual minority status  Gender minority status | Only examples | Link & Phelan 2001 | N/A | To examine associations among adolescents’ depressive symptoms and several adolescent-focused manifestations of stigma | Nine state-level indicators: laws and policies (i.e., state bans on conversion therapy, legislation focused on antibullying measures for LGBTQ+ youth, protections for LGBTQ+ youth in foster care, and nondiscrimination legislation); support for policies and laws that protect sexual minority Individuals; implicit attitudes toward sexual minority people from Project Implicit; density of gender-sexuality alliances; and proportion of LGBTQ+ public officials |
| Lattanner & Hatzenbuehler 2023 | United States | 315 gay men | Sexual minority status | Yes | Hatzenbuehler & Link 2014 | Minority stress  Other (Need to belong) | To examine whether individual, interpersonal, and structural levels of stigma prospectively affect loneliness and social support by thwarting fundamental belonging needs | Quantified structural stigma by combining indicators of institutional policies (e.g., state laws), cultural norms (e.g., attitudes towards sexual minorities), and societal-level conditions (e.g., density of same-sex couples) at the state and county levels; at the state level, this was eight indicators of structural stigma: 1) presence of 33 state laws and policies related to sexual orientation; 2) explicit attitudes held towards acceptance of homosexuality and legality of same-sex marriage; 3) explicit policy-specific attitudes held toward rights for lesbian, gay, and bisexual people and same-sex couples; 4) implicit attitudes held towards gay men and lesbian women; 5) proportion of openly lesbian, gay, bisexual, and transgender elected government officials; 6) proportion of schools with gay-straight alliances (also known as Gender-Sexuality Alliances); 7) density of LGBT adults; and (8) density of same-sex couples; the county-level structural stigma index consisted of four indicators of structural stigma reflective of cultural norms and societal-level conditions: 1) explicit attitudes held towards gay men; 2) explicit attitudes held towards lesbian women; 3) implicit attitudes held toward sexual minority people; and 4) density of same-sex couples |
| Martino et al. 2023 | United States | 10,414 youth, including 704 lesbian, gay, and bisexual youth | Sexual minority status  Gender  Race/ethnicity | Yes | Hatzenbuehler & Link 2014 | N/A | To examine associations of structural stigma with internalizing and externalizing symptoms among female; lesbian, gay, and bisexual; Black; and Latinx youth | Used four measures of structural stigma aggregated to the state level: 1) the measure of structural stigma specific to sex encompassed 18 items, 12 of which reflected individual implicit and explicit attitudes toward sex and sex-related stereotypes obtained from Project Implicit and the General Social Survey, and the six remaining items were taken from prior state-level composite indices of women’s social status (i.e., factors relating to pay, political representation, and social and economic autonomy) acquired from sources such as the Bureau of Labor Statistics, the Current Population Survey, and the Center for American Women in Politics; 2) the measure of structural stigma specific to sexual orientation comprised eight indicators used in prior work, and were a) an index of state laws and policies related to sexual orientation (e.g., employment non-discrimination laws), b) explicit attitudes toward acceptance of homosexuality and legality of same-sex marriage, c) explicit policy-specific attitudes toward rights for lesbian, gay, and bisexual people and same-sex couples, d) implicit attitudes toward gay men and lesbian women, e) a weighted proportion of openly LGBTQ elected government officials, f) the proportion of public high schools with gay-straight (or gender-sexuality) alliances, g) estimated percentage of LGBT adults living in each state, and h) the estimated density of same-sex couples living in each state; 3) the measure of structural stigma specific to race comprised 31 items reflecting anti-Black racism assessing explicit attitudes toward race or racial prejudice, as aggregated from individual responses to Project Implicit, the General Social Survey, and the American National Election Survey (e.g., related to attitudes toward Black people, endorsement of racial stereotypes, and perceptions of both the existence of racial prejudice and the impact of racial discrimination); and 4) the measure of structural stigma specific to Latinx ethnicity consisted of 3 indicators, including two separate feelings thermometers of explicit attitudes toward Latinx people and immigrants acquired from the American National Election Survey, as well as a composite index of state-level immigration policies |
| Oldenburg et al. 2015 | United States | 4908 men who have sex with men | Sexual minority status | Yes | Hatzenbuehler & Link 2014 | N/A | To investigate the relationship between structural stigma and HIV prevention among men who have sex with men | Used as "state-level structural stigma,” constructed via composite variable consisting of four components: density of same-sex couples, proportion of public high schools with a Gay-Straight Alliance, state policies and laws related to sexual orientation discrimination, and public opinion toward homosexuality |
| Perez-Brumer et al. 2015 | United States | 1229 transgender adults | Gender minority status | Yes | Hatzenbuehler & Link 2014  Hatzenbuehler et al. 2014 (retracted) | N/A | To assess individual (i.e., internalized transphobia) and structural forms of stigma as risk factors for suicide attempts among transgender adults | State-level structural stigma was operationalized as a composite index, including: density of same-sex couples; proportion of Gay-Straight Alliances per public high school; 5 policies related to sexual orientation discrimination; and aggregated public opinion towards homosexuality |
| Solazzo et al. 2020 | United States | 7476 people | Sexual minority status | No | Hatzenbuehler, Jun, Corliss, & Austin 2014 | Minority stress | To investigate associations between maternal comfort with lesbian, gay, and bisexual people during the participant’s adolescence and their health indicators in adulthood | Operationalized as a covariate using a measure of state-level structural stigma that includes policy, cultural acceptance, density of same-sex couples, and Gay-Straight Alliances in public schools |
| Titus et al. 2021 | United States | 108,977 adults, including 3,174 sexual minority adults | Sexual minority status | Yes | Hatzenbuehler 2016 | Minority stress | To examine the relationship between structural stigma and smoking prevalence among sexual minority and heterosexual adults | Used “state-level structural stigma" as assessed by an index of three primary components: state-level policies relating to sexual minorities, the state level density of same-sex couple households, and public opinion toward same-sex marriage; state-level policies included laws regarding same-sex marriage, laws regarding the inclusion of sexual orientation as a protected category under hate crime laws, and laws prohibiting discrimination on the basis of sexual orientation in employment, housing, and public |
| Engaged with laws and government-level policies as well as institutional policies, practices, and procedures (*n* = 2) | | | | | | | | |
| Hughes et al. 2022 | United States | 11,994 trans adults using hormones | Gender minority status | Only examples | White Hughto, Resiner, & Pachankis 2015 | N/A | To examine whether healthcare policy stigma is associated with using non-prescribed hormone and test possible mediational pathways | Explicitly linked to the concept of "healthcare policy stigma," which was measured via the presence of absence of: a) private insurance protections for trans people, b) whether or not Medicaid covers trans-specific healthcare, c) state-wide nondiscrimination protections, and d) religious exemption laws |
| Perrin et al. 2019 | United States | 732 men who identified as gay and fathers | Sexual minority status | Yes | Hatzenbuehler 2016 | N/A | To discover whether gay men continue to encounter barriers in becoming fathers and stigma in various contexts and to examine associations between these experiences and legal and social structures that surround these families | Operationalized as states’ laws and policies and the stated beliefs of religious institutions |
| *Engaged with laws and government-level policies; institutional policies, practices, and procedures; and additional structural factor(s) (n = 0)* | | | | | | | | |
| Engaged with laws and government-level policies; sociocultural attitudes and norms; and institutional policies, practices, and procedures (*n* = 0) | | | | | | | | |
| *Engaged with laws and government-level policies; sociocultural attitudes and norms; institutional policies, practices, and procedures; and additional structural factor(s) (n = 2)* | | | | | | | | |
| Lattanner et al. 2021 | United States | 502 gay and bisexual men | Sexual minority status | Yes | Hatzenbuehler & Link 2014 | Minority stress  Other (Cognitive-affective-behavioral model of concealment) | To examine the relationship between structural stigma and concealment motivation | Used eight participant response items to measure perceived structural stigma, as well as many indicators across three levels of “objective” structural stigma: 1) state-level structural stigma was measured using eight indicators of a) presence of 33 protective and discriminatory state laws and policies related to sexual orientation, b) explicit attitudes toward acceptance of homosexuality and legality of same-sex marriage, c) explicit policy-specific attitudes toward rights for lesbian, gay, and bisexual people and same-sex couples, d) implicit attitudes toward gay men and lesbian women, e) proportion of openly LGBT elected government officials, f) proportion of schools with gay–straight alliances (also known as gender–sexuality alliances), g) density of LGBT adults, and h) density of same-sex couples; 2) county-level structural stigma was measured using four indicators: a) explicit attitudes toward gay men, b) explicit attitudes toward lesbian women, c) implicit attitudes toward sexual minorities, and d) density of same-sex couples; and 3) city-level structural stigma was measured using the five indicators of a) presence of nondiscrimination laws and policies related to sexual orientation, b) presence of equality in benefits and protections provided to LGBT persons employed by the city and their partners, c) availability of city services and programs for LGBT residents, d) quality of relationship between law enforcement and the LGBT community, and e) explicit commitment by city leadership to inclusive practices and equality |
| Lee et al. 2022 | Uganda | 57 people over 18 assigned to the intervention cohort | Mental health | Yes | Hatzenbuehler 2016 | N/A | To evaluate the impact of a community‑led, theater‑based destigmatization campaign for mental illness | Structural stigma only used in the quantitative analysis in this multi-methods study; operationalized as responses to several scales including the Broad Acceptance Scale, the Fear and Behavioral Intentions towards the mentally ill questionnaire, selected items from the Community Attitudes to Mental Illness scale, and a modified version of a questionnaire developed for the World Psychiatric Association: Program to Reduce Stigma and Discrimination |
| Engaged with only sociocultural attitudes and norms (*n* = 11) | | | | | | | | |
| Hatzenbuehler et al. 2014 | United States | 21,045 people, including 914 sexual minority people | Sexual minority status | No | Link & Phelan 2001  Corrigan, Watson, Heyrman et al. 2005 | Minority stress | To investigate whether structural stigma increases risk of premature mortality for sexual minority people | Operationalized as community-level prejudice related to homosexuality using four items assessing respondents’ attitudes aggregated at the community level: 1) “If some people in your community suggested that a book in favor of homosexuality should be taken out of your public library, would you favor removing this book, or not?” 2) “Should a man who admits that he is a homosexual be allowed to teach in a college or university, or not?” 3) “Suppose a man who admits that he is a homosexual wanted to make a speech in your community. Should he be allowed to speak, or not?” 4) “Do you think that sexual relations between two adults of the same sex is always wrong, almost always wrong, wrong only sometimes, or not wrong at all?” |
| Hatzenbuehler et al. 2020 | United States | 21,791 adults, including 629 who reported any same-sex sexual partners in the past year | Sexual minority status | Yes | Hatzenbuehler & Link 2014 | Other (Social identity threat) | To examine whether the relationship between structural stigma and all-cause mortality is present among gay men/lesbian women as identified by individuals who reported same-sex sexual partners in the past year | Operationalized as community-level prejudice related to homosexuality using four items assessing respondents’ attitudes aggregated at the community level: 1) “If some people in your community suggested that a book in favor of homosexuality should be taken out of your public library, would you favor removing this book, or not?” 2) “Should a man who admits that he is a homosexual be allowed to teach in a college or university, or not?” 3) “Suppose a man who admits that he is a homosexual wanted to make a speech in your community. Should he be allowed to speak, or not?” 4) “Do you think that sexual relations between two adults of the same sex is always wrong, almost always wrong, wrong only sometimes, or not wrong at all?” |
| Hatzenbuehler, Flores, & Gates 2017 | United States | 279,053 individuals, including 9.584 sexual and gender minority individuals | Sexual minority status | Yes | Hatzenbuehler & Link 2014 | N/A | To examine sexual and gender minority adults' health consequences related to low versus high levels of community support for same-sex marriage | Used a measure of county-level attitudes toward same-sex marriage ("Do you favor or oppose allowing gays and lesbians to marry?") that was dichotomized to indicate support or opposition to same-sex marriage |
| Huang et al. 2020 | Taiwan | 1527 lesbian, gay, and bisexual adults, including 715 women and 812 men | Sexual minority status | Yes | Hatzenbuehler & Link 2014 | Minority stress  Sexual stigma conceptual framework | To investigate 1) how perceived attitudes toward lesbian, gay, and bisexual issues from different social realms (i.e., society, heterosexual friends, and family members) were associated with lesbian, gay, and bisexual people’s mental health, and 2) whether self-acceptance mediated the effects of perceived attitudes | Positioned perceived attitudes toward lesbian, gay, and bisexual issues (i.e., acceptance of homosexuality and support of same-sex marriage) as perception of structural stigma |
| Kellogg et al. 2023 | United States | Census data from all United States counties | Age | Yes | Hatzenbuehler & Link 2014 | Other (Stereotype embodiment theory) | To examine whether county-level attitudes toward older adults predicted older adults’ mortality rates | Operationalized as county-level structural stigma scores using poststratification of Project Implicit data on explicit and implicit a) age bias, b) bias toward sexual minorities, and c) bias toward Black people |
| Mellen & Hatzenbuehler 2023 | Sweden | 453 young adults who reported a history of either sexual assault or intimate partner sexual violence | Sexual violence | Yes | Hatzenbuehler & Link 2014 | N/A | To adapt a scale to measure individuals’ multi-level experiences with sexual violence exposure and examine associations between sexual violence stigma and anxiety, depression, posttraumatic stress disorder, alcohol use, shame, and perceived treatment need | A participant-response measure of HIV-related stigma was adapted for sexual violence stigma reflecting three levels of stigma, including structural-level stigma |
| Morey et al. 2018 | United States | 13,242 individuals | Immigrant status | Yes | Hatzenbuehler & Link 2014 | N/A | To assesses whether anti-immigrant prejudice at the community level is prospectively associated with mortality | Operationalized as "anti-immigrant prejudice (at the community level)" in analysis using social attitudes items |
| Pachankis et al. 2023 | United States | 120 LGBTQ youth (ages 16–25) reporting depression and/or anxiety symptoms | Sexual minority status | Only examples | Hatzenbuehler & Pachankis 2021 | Minority stress | To examine the feasibility, acceptability, preliminary efficacy, and multi-level stigma moderators of LGBTQ-affirmative internet-based cognitive-behavioral therapy | Multi-method study, but only used in quantitative analysis part; a county-level index of aggregated explicit and implicit attitudes toward LGBTQ people among residents of their county derived from Project Implicit |
| Pu & Xu 2023 | China | 779 sexual minority women, including 265 lesbian women and 193 bisexual women | Sexual minority status | Yes | Hatzenbuehler & Link 2014 | Minority stress  Other (Confucianism) | To explore how multiple levels of sexual minority stigma are related to parenting desire among Chinese sexual minority women | Part of multilevel stigma framework used to frame quantitative, with Confucianism mentioned as a form of structural stigma and measured using the participant-response Confucian Value Scale |
| Regnerus 2017 | United States | 21,045 people, including 914 sexual minority people | Sexual minority status | No | Hatzenbuehler et al. 2014 (retracted) | Minority stress | To replicated and then to assess alternative explanations for the findings in a prior study of structural stigma and all-cause mortality in sexual minority populations | Conceptual framework used in original study being re-analyzed in this one; followed the approach of constructing a structural stigma variable from four General Social Survey items |
| Xu et al. 2022 | China | 747 sexual minority adults | Sexual minority status | Yes | Hatzenbuehler & Link 2014 | Minority stress  Other (Confucianism and psychological mediation framework) | To explore how Confucianism and stigma were associated with the intention to pursue a heterosexual marriage among Chinese sexual minority individuals as well as the moderating mechanisms of gender and age | Confucianism mentioned as a form of structural stigma and measured using the participant-response Confucian Value Scale |
| *Engaged with sociocultural attitudes and norms and additional structural factor(s) (n = 2)* | | | | | | | | |
| Hollinsaid et al. 2023 | United States | 502 gay and bisexual men | Sexual minority status | Only examples | Hatzenbuehler 2016 | N/A | To examine whether sexual minority people employ particular emotion regulation processes in counties characterized by higher (vs. lower) structural stigma | Measured county-level structural stigma by combining four indicators: explicit attitudes toward gay men; explicit attitudes toward lesbian women; implicit attitudes toward sexual minorities; and density of same-sex couples—attitudes were from Project Implicit |
| Reid et al. 2014 | United States | 70 independent studies, including 99 separate interventions | Race/ethnicity | No | N/A | N/A | To examine the extent to which efficacy of interventions to improve African Americans’ condom use practices was moderated by two indicators of structural stigma (i.e., Whites’ attitudes toward African Americans and residential segregation in the communities where interventions occurred) | Operationalized as: 1) White people's community-level attitudes toward African Americans (using data from the American National Election Studies’s feelings thermometer), and 1) residential segregation approximated using dissimilarity scores, which reflect the proportion of African American residents who would need to move across census tracts in order to obtain an even distribution of African Americans across an entire metropolitan area |
| Engaged with sociocultural attitudes and norms as well as institutional policies, practices, and procedures (*n* = 5) | | | | | | | | |
| Clough et al. 2020 | Australia | 221 mental health professionals | Mental health | Yes | Corrigan, Markowitz, & Watson 2004  Other (Corrigan et al. 2010) | N/A | To develop and validate a scale (the Mental Health Professional Stigma Scale) for stigma around mental health professionals' seeking help for occupational stress and burnout | Used as a name of a factor that emerged from factor analysis (i.e., Perceived Structural Stigma) that was then used in psychometric analyses |
| Clough, Ireland, & March 2019 | Australia | 200 (38 male, 162 female) doctors | Mental health | Yes | Other (Beyond Blue 2015) | N/A | To develop and investigate the psychometric properties of the Stigma of Occupational Stress Scale for Doctors | Concept used in scale development as a category of items developed; factor named as perceived structural stigma following exploratory factor analysis |
| Favre et al. 2023 | Switzerland | 308 physicians | Mental health (burnout) | Yes | Other (Clough, Ireland, & March 2019) | Other multiple frameworks (Link & Phelan's discrimination model and the Maslach model of burnout) | To investigate the potential relationship between stigma and burnout symptomatology among medical doctors working in a university hospital | Perceived structural stigma was a subscale of the scale used (i.e., the Stigma of Occupational Stress Scale for Doctors [SOSS-D]) |
| Pachankis, Eldahan, & Golub 2016 | United States | 273 gay and sexual men ages 18-29 | Sexual minority status | No | N/A | Minority stress | To investigate migration-related motivations, experiences, health risks, and their associations among young gay and bisexual men in New York City | Operationalized as "hometown structural stigma" using 10 items adapted from the Growing Up LGBT in America study, in which participants rated 10 institutions in their hometowns (e.g., workplace and high school) in terms of acceptance |
| Wainberg et al. 2016 | Brazil | 641 sexually active adults attending public outpatient psychiatric clinics | Mental health | No | N/A | N/A | To examine the relationship between gender, severe mental illness diagnosis, and stigma experiences related to sexuality among people in psychiatric outpatient care | Part of conceptual framework; operationalized interchangeably with structural discrimination as subscale in measurement |
| *Engaged with sociocultural attitudes and norms; institutional policies, practices, and procedures; and additional structural factor(s) (n = 0)* | | | | | | | | |
| Engaged with only institutional policies, practices, and procedures (*n* = 1) | | | | | | | | |
| Gray et al. 2021 | Democratic Republic of the Congo | 1882 narratives from men and women over the age of 13 | Sexual violence (exploitation and abuse) | Yes | Hatzenbuehler 2016  Other (Corrigan & Watson 2002) | N/A | To examine how the degree of exposure to sexual exploitation and abuse affects community perceptions of a woman or girl’s (1) social status (public stigma) and (2) institutional support in her community (structural stigma) | Explicitly linked to the construct of inadequate institutional support, which was defined as a perceived absence of or lack of support to the affected woman or girl by individuals in power |
| *Engaged institutional policies, practices, and procedures and additional structural factor(s) (n = 5)* | | | | | | | | |
| Cheng et al. 2015 | United States | 50 people with psychosis who are Chinese and have immigrated to the United States | Immigrant status  Mental health | Yes | Yang et al. 2007  Other (Yang et al. 2014) | Other multiple frameworks (Structural vulnerability and What Matters Most) | To explore social and structural forms of stigma experienced by Chinese people with psychosis who have immigrated to the United States | Presented descriptive statistics of responses to structural stigma items from an adapted version of the Consumer Experience of Stigma Questionnaire |
| Pianchob, Muangpaisan, & Siritipakorn 2017 | Thailand | 193 family caregivers of people with Alzheimer's disease | Dementia | No | N/A | N/A | To evaluate the validity and reliability of the Family Stigma in Alzheimer’s Disease Scale (FS-ADS) (Thai version) in family caregivers of persons with Alzheimer’s disease | Name of a subscale of a scale evaluated in this psychometrics analysis |
| Tudose et al. 2017 | Romania | 76 people who are relatives of people with mental disorders | Dementia | Yes | Other (Corrigan 2005) | N/A | To 1) assess the perceptions and experiences of stigma as well as the burden of care among the relatives of persons with mental disorders who were admitted in a psychiatric hospital, and 2) compare these perceptions and experiences between relatives of patients with dementia versus other mental health disorders | Operationalized via the structural stigma component of the Family Stigma in Alzheimer’s Disease Scale (FS-ADS) |
| Werner et al. 2012 | Israel | 185 adults who were children of and caregivers for people with Alzheimer's disease | Dementia | No | N/A | Other (Attribution theory) | To examine whether family stigma is a predictor of caregiver burden in the case of Alzheimer’s disease | Name of a subscale in the Family Stigma in Alzheimer’s Disease Scale (FS-ADS) |
| Werner, Goldstein, & Heinik 2011 | Israel | 185 adult children of people with Alzheimer's disease | Dementia | Yes | Corrigan & Kleinlein 2005  Schulze & Angermeyer 2003 | N/A | To develop and examine the validity of the Family Stigma in Alzheimer’s Disease Scale | Domain of items for the measure being developed in the study based on prior qualitative research |
| Only engaged with additional structural factor(s) (*n* = 10) | | | | | | | | |
| Corrigan, Watson, Gracia et al. 2005 | United States | 3353 newspaper stories | Mental health | Yes | Other (Hill 1988; Merton 1948; Pincus 1999; Wilson 1990) | N/A | To examine trends in news media reporting on topics related to mental illness | Used as the overarching category of themes developed for content analysis |
| Croff et al. 2017 | United States | 210 people who had visited a gay bar across two separate studies | Sexual minority status | No | N/A | N/A | Article presents two studies with objectives to: (1) determine how safe and comfortable individuals felt while at an LGBT-friendly bar, and (2) to compare levels of safety and comfort as well as behavior change of LGBT individuals and allies who frequent LGBT-friendly bars since the Pulse Nightclub Massacre in Orlando, Florida | Used as the framing and sampling frame for quantitative study; not formally measured but residence in specific cities used as proxy indicators of high versus low structurally stigmatizing environments |
| DeLuca, Clement, & Yanos 2017 | United States | 951 people (national convenience sample) | Mental health | Yes | Corrigan, Markowitz, & Watson 2004 | N/A | To examine the influence of individual and social-level characteristics, including the endorsement of explicit individual stigma, on mental health funding decisions | Operationalized as responses to a Resource Allocation Test, in which participants assign money from a constrained budget to different types of programs |
| Frost 2020 | United Kingdom | 311 adults who migrated and now live in the United Kingdom | Immigrant status | Yes | Hatzenbuehler 2016 | Minority stress | To examine the extent to which the outcome of the Brexit referendum was associated with the mental health of migrants in the United Kingdom as a result of increased discrimination | Stated that the percentage of "leave" votes in the Brexit referendum (i.e., referendum asking if voters think the United Kingdom should stay or leave in the European Union) in an individual's electorate of residence can be considered an indicator of structural stigma toward migrants because negative attitudes toward migrants and restriction of migration were key factors in the leave vote |
| Lee, Butts, & Schneider 2023 | United States | 618 young Black men who have sex with men, who reported data about their social network | HIV | Yes | Other (Lee & Butts 2020) | Other (Network analysis) | To propose a novel approach to defining and estimating HIV stigmatization through the structure of sexual relations, as opposed to attitudes | Used social network characteristics as a proxy for structural stigma, specifically: 1) a reduced propensity towards HIV serodiscordant partnerships (exclusion); and 2) a reduced propensity towards partnerships with seroconcordant individuals who themselves have serodiscordant partnerships (ostracism) |
| Martin, Schofield, & Butterworth 2022 | Australia | 8290 newspaper articles | Welfare receipt | No | Link & Phelan 2014  Other (Tyler 2018, 2020) | Other multiple frameworks (Stigma power and stigma machine) | To examine the role of the media in perpetuating negative characterizations of people receiving welfare | Used as the type of stigma under investigation in a quantitative content analysis |
| Perales & Todd 2018 | Australia | 16,081 individuals | Sexual minority status | Yes | Link & Phelan 2001 | Minority stress | To examine 1) the influence of structural stigma on the life satisfaction, mental health, and overall health of sexual minority people, and 2) the mediating role of perceived social support | Operationalized as yes/no votes on national plebiscite on same-sex marriage (specifically, the no votes out of total eligible voters rather then no votes out of total actual votes) |
| Saxby, Chan, & Bavinton 2022 | Australia | 43,811 responses from gay, bisexual, and other men who have sex with men* | Sexual minority status | Yes | Hatzenbuehler 2016 | N/A | To explore whether structural stigma is associated with sexual health outcomes among Australian gay, bisexual, and other men who have sex with men | Operationalized as regional percentage of votes against legalizing same-sex marriage |
| Saxby, de New, & Petrie 2020 | Australia | 9,336,811 adults in relationships | Sexual minority status | Yes | Hatzenbuehler 2016 | Minority stress | To explore the extent to which structural stigma is associated with sexual orientation disparities in healthcare service and prescription medicine use | Operationalized as regional percentage of votes against legalizing same-sex marriage |
| Scheer, Pachankis, & Bränström 2022 | Multi-country (Europe) | 42,000 women, including 724 sexual minority women | Gender  Immigrant status  Sexual minority status  Socioeconomic status | Yes | N/A | N/A | To 1) determine whether gender-based structural stigma is associated with intimate partner violence and related circumstances among European women; 2) examine minority-majority intimate partner violence disparities; and 3) assess whether structural stigma is associated with intimate partner violence disparities | Operationalized only via the Gender Equality Index (not operationalized as variable for other stigmatized statuses in study); the Gender Equality Index is an index of the gender gap (i.e., differences between women and men) in six aggregated political and public policy domains: work, money, knowledge, time, power, and health |

**Additional Table 2.** Details of articles that qualitatively operationalize structural stigma (*N* = 68)

| **Author Date** | **Setting** | **Sample** | **Stigmatized status(es) of interest** | **Definition of structural stigma given** | **Source(s) of structural stigma concept** | | **Additional theories/ conceptual frameworks used** | **Objective of study** | **Notes on how structural stigma was operationalized** |
| --- | --- | --- | --- | --- | --- | --- | --- | --- | --- |
| Engaged with only laws and government-level policies (*n* = 5) | | | | | | | | | |
| Azagba, Ebling, & Hall 2023 | United States | Laws from all 50 states in the United States | Substance use (alcohol use) | Yes | Other (Link & Hatzenbuehler 2016) | | N/A | To examine the current status and trends of alcohol exclusion laws across states in the United States | Used to conduct a systematic legal analysis in which alcohol exclusion laws were considered a form of structural stigma |
| Corrigan, Watson, Heyrman et al. 2005 | United States | 968 bills | Mental health | No | N/A | | N/A | To examine trends in structural stigma in state legislation related to people with mental illness | Used in the application of codebook of themes related to structural stigma developed via focus group discussion and reviewed by mental health attorney experts; however, almost exclusively referred to as "structural discrimination" throughout the article |
| Fredrick et al. 2021 | United States | 187 sexual and gender minority adults | Sexual minority status  Gender minority status | Yes | Hatzenbuehler & Link 2014 | | Minority stress | To examine the anticipated structural stigma that sexual and gender minority individuals reported following the 2016 U.S. presidential election | Used as an overarching thematic domain of inductively identified subthemes |
| Frey et al. 2021 | United States | 16 adults who identify as lesbian, gay, bisexual, or pansexual | Sexual minority status | Yes | Other (Herek 2007) | | Minority stress  Sexual stigma conceptual framework | To explore experiences related to sexual identity stigma among adults who identify as lesbian, gay, bisexual, and pansexual in the United States South | Used as an inductively identified theme that arose during content analysis |
| Huebner, Kras, & Pleggenkuhle 2018 | United States | 62 men and women on parole for sexual offenses | Incarceration | Yes | Link & Phelan 2001 | | Goffman (1963) | To provide insight into how individuals experience and navigate reentry to communities and contexts that may impede or influence success | Used as part of conceptual frameworks used as sensitizing concepts for inductive and deductive analysis |
| *Engaged with laws and government-level policies and additional structural factor(s)* (*n* = 1) | | | | | | | | | |
| Kim & Dawson 2023 | South Korea | 29 people, including 23 high-performance coaches with experience as elite athletes and 6 people who were involved in the school environment and interacted directly with high-performance coaches | Occupation (coaching) | Yes | Other (Hannem 2012) | | Goffman (1963) | To explore how sports coaches’ identity and social relations are shaped within the context of new policy initiatives in sport | The research explored participant experience in the context of structural stigma from Integrity Initiatives from the Office of Education as well as mass media |
| Engaged with laws and government-level policies as well as sociocultural attitudes and norms (*n* = 5) | | | | | | | | | |
| Conway et al. 2023 | Australia | 40 people receiving and 29 people providing opioid agonist treatment | Substance use (opioid use) | No | Other (McCradden et al. 2019) | | Other multiple frameworks (Rhodes’s risk environment framework and complex adaptive systems theory) | To understand how adaptations in the complex system of opioid agonist treatment provision impacted and responded to risk environments of people receiving opioid agonist treatment during the COVID-19 pandemic | Used as a subtheme of one of four themes that were identified in data analysis |
| Elkhalid, Morrow, & Leong 2023 | United States | 15 individuals who were a current partner in a citizen/non-citizen relationship | Immigrant status | Yes | Hatzenbuehler & Link 2014 | | Other multiple frameworks (Relational turbulence theory and stigma management theory) | To examine citizen/non-citizen partners’ social experiences relating to stigma and citizenship status | Positioned anti-immigration rhetoric and the uncertainty surrounding policy changes with the then-upcoming Trump administration as structural stigma |
| Gunasekaran et al. 2022 | Singapore | 17 healthcare professionals who work in mental health settings | Mental health | No | N/A | | N/A | To explore how stigma affects recovery from the perspectives of healthcare professionals that work closely with people with mental illness | Used as an inductively identified theme in thematic analysis |
| Hubach et al. 2019 | United States | 40 sexual minority men | Sexual minority status | Yes | Hatzenbuehler & Link 2014 | | N/A | To explore how cultural context within rural communities enhances or protects against stigma and provide a more nuanced understanding of the mechanisms by which stigma, as an etiologic factor, influences health care access and utilization in these settings | Used as an inductively identified theme during grounded theory approach |
| Meadows et al. 2021 | Multi-continent | 3 anti-discrimination developments | Fatness | No | N/A | | Intersectionality | To highlight how the creation, implementation, and enforcement of legal and policy mechanisms that prohibit weight discrimination ironically suffer under the very burden of deeply rooted structural stigmas against fatness and fat bodies that such efforts seek to counter | Used as rationale for legal analysis; looked for in interpretation of legal documentation |
| *Engaged with laws and government-level policies; sociocultural attitudes and norms; and additional structural factor(s)* (*n* = 0) | | | | | | | | | |
| Engaged with laws and government-level policies as well as institutional policies, practices, and procedures (*n* = 7) | | | | | | | | | |
| Abi Hana et al. 2022 | Lebanon | 45 total people, including policy makers (n = 3), primary health care center management (n = 4), primary health care center (n = 24), and people who use primary health care centers (n = 14) | Mental health | No | Pescosolido & Martin 2015 | | N/A | To explore stigma associated with mental illness at primary health care centers and inform a deeper understanding regarding the integration of mental health into primary health care | Used as one of three thematic domains of codes and one of three themes used to present results; codebook applied was previously developed for a similar study in Tunisia |
| AboJabel et al. 2021 | Israel | 22 people who are spouses to people with Parkinson's disease | Parkinson's disease | No | N/A | | N/A | To explore the perceptions and stigmatizing experiences of family caregivers of people with Parkinson’s disease | Used as an inductively-derived theme discussed as structural stigma in discussion; however, reported as "structural discrimination" in results |
| Dobransky 2020 | Multi-continent | 28 mental health care providers | Mental health | Yes | Corrigan, Markowitz, & Watson 2004  Hannem & Bruckert 2012  Hatzenbuehler & Link 2014  Pescosolido & Martin 2015  Other (Tyler & Slater 2018) | | Other (Risk containment) | To explore how the stigma of mental health treatment competes with other stigmatized identities | Used as part of the overarching conceptual model/rationale for analysis; data analyzed using grounded theory approach but then stigma was explored abductively |
| Earnshaw et al. 2023 | Malaysia | 34 individuals including 12 men who have sex with men, 8 people living with HIV, 7 transgender women, 7 female sex workers, and 6 people who inject drugs (categories not mutually exclusive) | HIV | Yes | Hatzenbuehler & Link 2014  Hatzenbuehler 2017 (Handbook) | | N/A | To explore Malaysian key populations’ and people living with HIV's experiences with and hopes for doctor interaction | Used as the context and overarching concept for investigation |
| Fabbre & Gaveras 2020 | United States | 88 transgender and gender nonconforming adults aged 50 and older | Gender minority status | Yes | White Hughto, Resiner, & Pachankis 2015  Hatzenbuehler, Keyes, & Hasin 2009  Other (Hatzenbuehler & Keyes 2013) | | White Hughto et al.’s (2015) multilevel model of stigma toward trans people | To explore the manifestation of multi-level stigma in the lives of transgender and gender nonconforming older adults | Used as part of a multilevel conceptual framework to guide interpretative content analysis in the deductive analysis phase |
| Werner, Goldstein, & Buchbinder 2010 | Israel | 10 family caregivers of community-dwelling people with Alzheimer's disease | Dementia | Yes | Corrigan & Kleinlein 2005  Schulze & Angermeyer 2003 | | N/A | To explore the subjective experience of family stigma as reported by children of persons with Alzheimer’s disease | Seen as a dimension of stigma that emerged in open coding |
| Whittle et al. 2017 | United States | 64 people who are low-income and living with HIV and/or type 2 diabetes mellitus | Welfare receipt | Yes | Hatzenbuehler & Link 2014 | | Intersectionality | To investigate how the system of disability benefits, as well as the related stigmatizing discourses, shapes the lived experience of disabling physical illness in today's United States | Used as part of the theoretical frameworks employed; used to frame results but not the label of a theme or domain in results |
| *Engaged with laws and government-level policies; institutional policies, practices, and procedures; and additional structural factor(s)* (*n* = 0) | | | | | | | | | |
| Engaged with laws and government-level policies; sociocultural attitudes and norms; and institutional policies, practices, and procedures (*n* = 8) | | | | | | | | | |
| Benintendi et al. 2021 | United States | 41 people with chronic, non-cancer pain who had undergone a reduction in opioid daily dosage of ≥50 % in the past two years | Chronic pain | Yes | Hatzenbuehler 2016 | | N/A | To describe the ways in which well-intentioned taper initiatives impacted people living with chronic pain | Used as an inductively identified theme that rose to prominence during conducting the research |
| Broussard 2020 | Multi-country (Europe) | 68 cisgender women who had sought abortion services | Abortion | Yes | Hatzenbuehler & Link 2014 | | Other ([De]medicalization) | To examine the ways structural stigma influences women’s embodied experiences of abortion | Used a part of a deductively applied theoretical framework for investigation and analysis |
| Coulter et al. 2022 | United States | 60 sexual and gender minority youth and 29 school staff members | Sexual minority status  Gender minority status | No | N/A | | Minority stress | To investigate 1) ways in which sexual and gender minority youth have been and would like to be supported, protected, and affirmed by high school staff members and 2) ways in which high school staff members have and would like to support, protect, and affirm sexual and gender minority youth; and to explore if these differ by level of structural stigma, race/ethnicity, and gender | Used as a sampling frame and axis of difference for analysis |
| Füllgrabe & Smith 2023 | Multi-continent | 11 people in consensual non-monogamous relationships | Polyamory | No | N/A | | Minority stress | To explore minority stress and resilience among individuals in consensual non-monogamous relationships | Used as a concept that was considered in the sampling, as well as a concept that has informed the primary researcher’s orientation; appeared as subtheme in results |
| Judd et al. 2023 | United States | 30 people with lived experience of opioid use disorder (i.e., in recovery from opioid use disorder or a family member of someone with opioid use disorder) | Substance use (opioid use) | Yes | Corrigan et al. 2005  Other (Leis and Rosenbloom 2009; Woo et al. 2017) | | N/A | To broaden the understanding of opioid use disorder by investigating how people directly affected by the opioid overdose epidemic describe their experiences of stigma | Emerged as a theme during inductive analysis |
| Kim et al. 2021 | United States | 50 administrators in charge of setting clinical protocols regarding options counseling | Abortion | Yes | Corrigan, Markowitz, & Watson 2004 | | Other multiple frameworks (Abortion aversion and stigma complex) | To examine how organizational policy utilization correlated with organization-level protocols for discussing abortion in options counseling interactions while the domestic gag rule policy was under legal contest | Used to explain and contextualize findings in results of a study that used an adapted grounded theory approach |
| Liegghio 2017 | Canada | 15 caregivers with children involved with mental health service | Mental health | Yes | Corrigan, Kerr, & Knudsen 2005  Other (Fox 2012; Hinshaw & Cicchetti 2000) | | N/A | To explore structural stigma in child and youth mental health, particularly caregivers' experiences with various services for supporting their children | Used as a thematic domain for themes that arose inductively during thematic analysis |
| Philbin et al. 2016 | United States | 31 Black men who have sex with men and 17 community stakeholders | Sexual minority status | No | N/A | | Social ecological model | To examine how all levels of the ecological framework must be addressed for PrEP to be successfully implemented as an effective HIV prevention approach | Used to frame the study; used as a theme in constant comparative analysis |
| *Engaged with laws and government-level policies; sociocultural attitudes and norms; institutional policies, practices, and procedures; and additional structural factor(s)* (*n* = 3) | | | | | | | | | |
| Davis et al. 2022 | Canada | 26 people who may use a drug checking service | Substance use (general drug use) | Only examples | N/A | | Other multiple frameworks | To explore how best to implement drug checking services to the wider population including those at risk of overdose | Part of multilevel stigma framework applied in this analysis |
| Lane 2023 | Canada | 30 people who self-identified as 2SLGBTQ and/or being an individual involved in the delivery of primary care service | Sexual minority status  Gender minority status | No | Hatzenbuehler & Link 2014  Link & Phelan 2014  Other (Bourdieu 1977) | | Intersectionality  Other (Critical theory) | To explore domains of power operating across multiple forms of oppression, think through subjective realities, and generate a nuanced rendering of power relations influencing health service delivery to diverse 2SLGBTQ populations | Used as a concept in the co-construction of a theory (“Working Through Stigma”) |
| O'Hara et al. 2023 | Singapore | 13 representatives from LGBTQI-affirming non-governmental organizations | Sexual minority status  Gender minority status | Only examples | Hatzenbuehler & Link 2014  Major, Dovidio, & Link 2018 | | Intersectionality  Other (Stangl et al.’s [2019] Health Stigma and Discrimination Framework) | To investigate stigma in healthcare for LGBTQI+ patients, as well as possible upstream factors within medical education | Used as both an overarching concept and theme in results |
| Engaged with only sociocultural attitudes and norms (*n* = 6) | | | | | |  | | | |
| Carlon 2022 | Australia | 1 cisgender woman (the author of the article) | Mental health | Yes | Hatzenbuehler & Link 2014 | | Other (Taboo) | To explore the experience of societal meanings of suicide from the perspective of people bereaved by suicide | Used as part of predefined conceptual framework (along with taboo) for autoethnography |
| Childs et al. 2021 | United States | 22 professionals working with people who use drugs | Substance use (general drug use) | No | N/A | | Other (Harm reduction) | To understand challenges and strategies for engaging communities in accepting harm reduction perspectives and services | Inductively coded as a relevant theme |
| Clark et al. 2022 | United States | 22 sexual and gender minority adults | Sexual minority status  Gender minority status | Only examples | Hatzenbuehler 2016 | | Minority stress  Other (Suicidality theories) | To identify specific determinants of how sexual and gender minority individuals acquire the capability to kill themselves | Used as an inductively derived thematic domain in analysis; also used to frame results in the discussion |
| Grace et al. 2018 | Canada | 16 gay men | HIV  PrEP use | No | N/A | | N/A | To learn about everyday social and sexual realities of PrEP use, including PrEP-related barriers and psychosocial challenges | Used as a thematic domain that emerged inductively during a grounded theory analysis |
| Villalona 2021 | United States | 10 people (patient shadowing) | Non-dominant language use | Yes | Hatzenbuehler & Link 2014 | | Other (Health-related deservingness) | To understand the challenges and experiences of Spanish-speaking patients with limited English proficiency in seeking emergency department care that is considered non-urgent | Used as a complementary framework in analyzing the emergent themes in the data collected, introduced in discussion to interpret results |
| Young & Valiotis 2020 | United Kingdom | 32 practitioners working with gay and bisexual men and African communities, including 19 community practitioners and 13 clinical practitioners | HIV  Sexual minority status | No | Other (Parker & Aggleton 2003) | | Other (Developing HIV Literacy framework) | To examine HIV literacy challenges in the context of PrEP provision within and across diverse communities | Used as a relevant theme that emerged during analysis |
| *Engaged with sociocultural attitudes and norms and additional structural factor(s)* (*n* = 0) | | | | | | | | | |
| Engaged with sociocultural attitudes and norms as well as institutional policies, practices, and procedures (*n* = 7) | | | | | | | | | |
| Bikos 2021 | Canada | 727 police officers who responded to a survey and 116 police officers who participated in semi-structured interviews | Mental health | Yes | Bos et al. 2013 | | N/A | To provide a preliminary, general overview of police officers’ perception of stigma toward mental illness in their workplace culture and its impacts | Inductively coded as a theme and framed as the intersection of culture and organizational structure |
| Chanda et al. 2017 | Zambia | 40 female sex worker peer educators | HIV  Sex work | No | N/A | | Social ecological model | To gain a more in-depth understanding of perceived barriers and facilitators of HIV testing among female sex workers | Coded either inductively or deductively as a structual-level barrier |
| Iott et al. 2022 | United States | 64 adult gay, bisexual, queer and other men who have sex with men | Sexual minority status | Yes | Hatzenbuehler & Link 2014 | | Intersectionality  Intersectional stigma | To identify gay, bisexual, queer, and other men who have sex with men's perspectives regarding how stigma influences their HIV testing decisions, including micro-decisions regarding whether, where, and how often to get tested | Used as a deductively applied code as part of an intersectional multilevel framing for thematic analysis |
| Krüsi et al. 2016 | Canada | 31 women street-based sex workers, including 26 cisgender women and 5 transgender women | Sex work | No | N/A | | Other multiple frameworks (Parker & Aggleton’s [2003] conceptualization of stigma, structural vulnerability/violence, and everyday and symbolic violence | To explore the complex ways in which coexisting stigmatizing assumptions of sex workers as 'risky' and 'at risk' intersect with evolving sex work policing strategies to shape street-based sex workers' civic rights, experiences of violence, and the negotiation of sexual risk reduction | Used as a concept for interpretation of emergent themes from a thematic analysis |
| Lanthier et al. 2023 | N/A | A sample of 186 Reddit posts | Sexual violence (assault) | Yes | Hatzenbuehler & Link 2014 | | Intersectionality  Intersectional stigma | To understand (non)disclosure to formal support providers among diverse sexual assault survivors | Used as part of the multilevel stigma framework used for the framing of the analysis and interpretation of results in discussion |
| Stevens et al. 2021 | Zambia | 18 women living with HIV | HIV | Yes | Corrigan, Watson, Heyrman et al. 2005  Other (Stevens et al. 2019) | | Other multiple frameworks (International Classification of Functioning, Disability and Health and Stevens et al.’s [2019] structural stigma model) | To explore how the International Classification of Functioning, Disability and Health, a rehabilitation framework, can provide a holistic understanding of stigma experiences of women living with HIV | Used as part of multilevel stigma framework used for interpretation and framing of qualitative case studies |
| Sukhera et al. 2022 | Multi-continent | 12 medical students, residents, and medical school faculty; Twitter data related to a selected index tweet | Mental health | Yes | Other (Corrigan 2004; Livingston 2013) | | Foucauldian thought | To explore medical learners' encounters with stigma and their understandings of how it is embedded in medical education and the clinical environment | Used as part of a conceptual framework for Foucauldian Critical Discourse Analysis |
| *Engaged with sociocultural attitudes and norms; institutional policies, practices, and procedures; and additional structural factor(s)* (*n* = 1) | | | | | | | | | |
| Schmitz et al. 2020 | United States | 41 LGBTQ+ Latino/a young adults | Race/ethnicity  Sexual minority status  Gender minority status | Yes | Hatzenbuehler & Link 2014 | | Intersectionality  Minority stress | To examine how structural stigmas related to LGBTQ+ young people’s multiply marginalized statuses may result in psychological stress and shape their perceptions of mental health | Used as part of an intersectional minority stress framework for analysis; shows up as structural racism structural gender norms, and structural stigma (anti-LGBTQ+) as thematic domains in results |
| Engaged with only institutional policies, practice, and procedures (*n* = 19) | | | | | | | | | |
| Antoniou et al. 2023 | Canada | 23 adults who completed direct-acting antiviral treatment or were about to begin such treatment | Hepatitis C  Substance use (drug use including injection drug use) | No | N/A | | Other multiple frameworks (Goffman, Link & Phelan [2001], and structural vulnerability) | To understand challenges to treatment uptake with direct-acting antivirals among people living with hepatitis C and compare treatment experiences between people who do and do not inject prescription and/or unregulated drugs | Used as a concept for investigation and interpretation of results |
| Blakey & Gunn 2018 | United States | 14 cisgender women who took part in the Supporting the End of Prostitution Permanently postadjudication prostitution court program and 8 professionals working for the program | Sex work | Yes | Link & Phelan 2001 | | N/A | To examine the ways that stigma complicated the exiting process for women with extensive histories of sex work | Used as an inductively derived thematic domain in analysis |
| Gagnon 2015 | Canada | 21 people living with HIV | HIV | Yes | Hannem & Bruckert 2012  Other (Foucault) | | Foucauldian thought | To 1) describe stigmatizing and discriminatory practices in health care settings, and 2) explore both symbolic and structural stigma from the perspectives of people living with HIV | Used as a deductively applied thematic domain |
| Hofer & Savell 2021 | United States | 48 police officers | Mental health | Yes | Hatzenbuehler & Link 2014 | | N/A | To understand officer-perceived barriers and facilitators to mental health service utilization to generate strategies for increasing the accessibility of mental health resources | Used as an identified thematic domain in inductive thematic analysis |
| Meyerson et al. 2014 | United States | 81 transcribed reports by ethnographer-informants | HIV | No | Corrigan, Markowitz, & Watson 2004  Hatzenbuehler, Link, & Phelan 2013  Pincus 1996 | | N/A | To examine evidence of institutional and structural stigma in the HIV testing process | Used as the central conceptual framing for analysis (contrasted with institutional stigma) that was then inductively coded in data |
| Mujugira et al. 2021 | Uganda | 50 trans men | Gender minority status | Yes | White Hughto, Resiner, & Pachankis 2015 | | N/A | To characterize HIV and STI risk, prevention needs, and sexual decision making among trans men | Used in an inductive content analysis of qualitative data that was then organized into a stigma framework |
| Mukamana et al. 2022 | Rwanda | 33 women living with HIV | HIV  Gender (motherhood) | Yes | Hatzenbuehler & Link 2014 | | Intersectionality  Intersectional stigma | To: 1) understand the sources of HIV-related stigma among women living with HIV, and 2) to understand the cultural, linguistic, and contextual context of HIV-related stigma and the intersection of HIV-related stigma to the HIV care continuum among women living with HIV | Used as one of the types of stigma being investigated |
| Ong et al. 2020 | Singapore | 42 people with mental illness | Mental health | Yes | Corrigan, Markowitz, & Watson 2004  Other (Rüsch et al. 2005) | | N/A | To identify the common encounters of mental illness stigma experienced by people with mental illness in Singapore and uncover their individual strategies and efforts to reduce mental illness stigma | Used as part of conceptual framework for inductive analysis; emerged as a thematic domain |
| Paterson, Hirsch, & Andres 2013 | Canada | 50 service providers in hospital emergency departments or community organizations that serve people who use drugs | Hepatitis C  Substance use (general drug use) | Yes | Yang et al. 2007  Other (Castro & Farmer 2005; Link & Phelan 2006) | | Other multiple frameworks (Bourdieusian thought and Parker & Aggleton’s [2003] conceptualization of stigma) | To identify structural factors that contribute to the structural stigmatization of people within hospital emergency departments who are current users of illicit drugs and are HCV positive | Used as a concept of interest; themes are factors that shape structural stigma |
| Ramos-Pibernus 2020 | United States | 29 trans men | Gender minority status | Yes | White Hughto, Resiner, & Pachankis 2015  Hatzenbuehler, McLaughlin, Keyes, & Hasin 2010 | | N/A | To document the stigmatization experiences faced by trans men in Puerto Rico and its impact on their overall health | Used as part of the multilevel framing and conceptual framework for analysis; thematic domain in inductive analysis |
| Ricciardelli et al. 2020 | Canada | 828 public safety personnel | Mental health | Yes | Hatzenbuehler & Link 2014 | | N/A | To better understand barriers to mental health care-seeking among public safety personnel | Used as part of conceptual framing for analysis; operationalized as thematic domain in a "semi-grounded approach" |
| Ritterbusch, Correa Salazar, & Correa 2018 | Colombia | 28 transgender women | Gender minority status | No | White Hughto, Resiner, & Pachankis 2015 | | Intersectionality | To present and discuss the stigma-related barriers to healthcare experienced by trans women and their experiences of multi-level violence within the healthcare system | Used as part of the conceptual framework for analysis; shows up as theme in inductive analysis |
| Shattuck et al. 2022 | United States | 309 people, including 91 implementation resource team leaders, 77 school administrators, and 132 implementation resource team members | Sexual minority status  Gender minority status | Yes | Hatzenbuehler 2014  Hatzenbuehler 2016  Link & Phelan 2001 | | Minority stress | To examine how power operates to hinder or promote the ability of school staff to change school environments, disrupt structural stigma, and increase safety and support for LGBTQ+ youth | Used as a highly related concepts; school policies (subject of the analysis) were seen as disrupting and also perpetuating structural stigma |
| Sukhera, Kulkarni, & Taylor 2021 | Canada | 22 medical professionals, including 17 resident physicians and 5 faculty members | Gender | Yes | Hatzenbuehler & Link 2014 | | Other (Moral and structural distress) | To explore how resident physicians perceive moral distress in relation to structural stigma | Used as part of dual conceptual framework for investigation and analysis of perceptions of structural stigma and its influence on moral distress (grounded theory; constant comparative analysis) |
| Syversten et al. 2021 | United States | 28 women with histories of opioid use who were pregnant or recently gave birth and 18 healthcare providers | Substance use (general drug use) | Yes | Other (Tsai et al. 2019) | | Other (Cascade of care) | To examine how drug-related stigma manifests across women’s pregnancy journeys to shape access and quality of care | Used as part of a multidimensional conceptual stigma framework for analysis; thematic domain in results; inductively emerged |
| Ugwu & Dumbili 2022 | Nigeria | 18 people who use cannabis; participant observation | Substance use (cannabis use) | Yes | Hatzenbuehler & Link 2014 | | Other (Extra-legal policing) | To explore cannabis use and community forming and how people who use cannabis manage police’s extrajudicial practices and stigma from the broader society | Used as part of conceptual framework for analysis; mentioned as a theme in results |
| Werner & Doron 2017 | Israel | 26 people, including 14 social workers and 8 lawyers | Dementia | Yes | Other (Corrigan et al. 2005) | | N/A | To explore the meaning and consequences of labeling on structural stigma in the context of Alzheimer’s disease in the legal system | Used as the type of stigma being investigated in thematic analysis |
| White Hughto et al. 2018 | United States | 20 transgender women who had been incarcerated within the past five years | Gender minority status | No | White Hughto, Resiner, & Pachankis 2015 | | White Hughto et al.’s (2015) multilevel model of stigma toward trans people | To 1) assess transgender women's experiences receiving physical-, mental-, and transition-related healthcare in carceral settings; and 2) document potential structural, interpersonal, and individual barriers to healthcare that can be targeted in future, multi-level intervention efforts to ensure access to quality, gender-affirmative care | Used as part of a multilevel stigma framework used as the theoretical framework for analysis |
| Woodgate et al. 2020 | Canada | 58 youth living with anxiety (ages 10-22) | Mental health | Yes | Hatzenbuehler & Link 2014 | | N/A | To explore youth's lived experiences of anxiety and the related multi-level stigma | Used as part of a multilevel stigma framework for analysis; thematic domain in results |
| *Engaged institutional policies, practice, and procedures and additional structural factor(s) (n = 1)* | | | | | | | | | |
| Cheng et al. 2015 | United States | 50 people with psychosis who are Chinese and have immigrated to the United States | Immigrant status  Mental health | Yes | Yang et al. 2007  Other (Yang et al. 2014) | | Other multiple frameworks (Structural vulnerability and What Matters Most) | To explore social and structural forms of stigma experienced by Chinese people with psychosis who have immigrated to the United States | Used as a domain in a scale administered during structured interviews, discussion of responses was qualitatively analyzed |
| Only engaged with additional structural factor(s) (*n* = 5) | | | | | | | | | |
| Corrigan, Watson, Gracia et al. 2005 | United States | 3353 newspaper stories | Mental health | Yes | Other (Hill 1988; Merton 1948; Pincus 1999; Wilson 1990) | | N/A | To examine trends in news media reporting on topics related to mental illness | Used as the overarching category of themes developed for content analysis; some themes came from prior research and some came from a focus group discussion conducted prior to content analysis |
| Lampropoulos, Wolman, & Apostolidis 2017 | France | 627 newspaper articles | Mental health | No | Corrigan, Watson, Gracia et al. 2005 | | Foucauldian thought | To explore how French newspapers contribute to the stigma of people with schizophrenia | Used as the type of stigma being investigated in content analysis |
| Schlehofer, Wagner, & Bramande 2023 | United States | 19 LGBTQ+ people | Sexual minority status  Gender minority status | Yes | Hatzenbuehler 2014  Other (Meyer 2003) | | Minority stress | To document responses to the 2020 United States presidential election among LGBTQ + people | Used as what is being investigated in analysis, particularly anticipated changes / anticipated structural stigma related to potential results of the 2020 US presidential election cycle |
| Soffer 2022 | Multi-continent | 225 mass media articles, including 117 from the United States and 108 from Israel | Cancer | Yes | Corrigan, Watson, Gracia et al. 2005 | | Other (Illness metaphors) | To explore the social construction of cancer-related stigma in mass media during the time of COVID-19 | Used as a rationale and interpretative framework for analysis on structural stigma (through media) |
| Villanueva Baselga 2020 | N/A | 2 interactive documentaries | HIV | Yes | Hatzenbuehler 2016 | | N/A | To examine interactive documentaries as a tool for raising awareness of the impact of HIV-related stigma and cultural trauma | Used as a framework for understanding how culture and media relate to health identities; brought up in analysis |

**Additional Table 3.** Details of articles that mention, but do not operationalize, structural stigma (*N* = 131), subdivided by method(s) used

| **Author Date** | **Setting** | **Sample** | **Stigmatized status(es) of interest** | **Definition of structural stigma given** | **Source(s) of structural stigma concept** | **Additional theories/ conceptual frameworks used** | **Objective of study** |
| --- | --- | --- | --- | --- | --- | --- | --- |
| Quantitative research (*n* = 89) | | | | | | | |
| Affuso et al. 2023 | Multi-country (Europe) | 490 adults ages 18-35 who identified as LGBTQ+ (307 from Italy and 183 from Spain) | Sexual minority status | No | Other (Frost 2011) | Minority stress  Social ecological model | To investigate, in a comparative perspective, the associations between minority stressors and mental health in Italian and Spanish gay and lesbian youth |
| Agénor et al. 2022 | United States | 30 sexual orientation- and gender identity-related state laws in 9 legal domains | Sexual minority status  Gender minority status | Yes | Hatzenbuehler 2014  Other (National Academies of Science Engineering Medicine 2020 | N/A | To: 1) develop a multiyear database of sexual orientation- and gender identity-related state laws to advance sexual and gender minority health research and practice, and 2) assess variability in state laws from 1996 through 2016 |
| Angermeyer et al. 2014 | Germany | Two population surveys of 5025 and 1232 people | Mental health | No | N/A | N/A | To examine whether attitudes referring to individual and structural discrimination develop in parallel or differently over time |
| Arnhart et al. 2022 | N/A | 700 peer-reviewed publications | Autism | No | N/A | N/A | To investigate adherence to person-centered guidelines among peer-reviewed research publications focused on autism and the co-occurrence of stigmatizing language in articles using person-first language and identity-first language styles |
| Asadi et al. 2023 | United States | Two samples: A clinical sample of 1,174 adults, 254 of whom were sexual and gender minority adults; and a non-clinical (i.e., university) sample of 1,456 adults, 151 of whom were sexual and gender minority adults | Sexual minority status  Gender minority status | No | Hatzenbuehler 2009  Pachankis et al. 2021  Other (Pachankis et al. 2014) | Minority stress | To formally test the measurement invariance properties of the Personality Inventory for DSM-5 Brief Form based on sexual and gender minority status |
| Boertien & Vignoli 2019 | United Kingdom | 476,411 people, including 4,112 individuals in coresidential same-sex relationships | Sexual minority status | No | Hatzenbuehler, Keyes, & Hasin 2009 | Minority stress | To investigate whether the subjective well-being of individuals in same-sex unions improved following the legalization of same-sex marriage in England and Wales in March 2014 |
| Böge et al. 2018 | India | 924 people from five metropolitan cities | Mental health | No | N/A | Other (Gender disparities) | To investigate perceived stigma toward mental illness in five metropolitan cities in India and explore the roles of relevant sociodemographic factors |
| Bränström & Pachankis 2018 | Sweden | 79,568 individuals, including 1,673 lesbian, gay, or bisexual individuals | Sexual minority status | No | N/A | Minority stress  Other (Syndemic theory) | To: 1) explore sexual orientation-based differences in substance use, psychological distress, and their co-occurrence in a representative sample, and 2) examine if greater exposure to stressors (e.g., discrimination, victimization/ threats, and social isolation) could explain these potential disparities and their co-occurrence |
| Brener et al. 2022 | Australia | 1,116 gay and bisexual men | Sexual minority status | No | N/A | Other (Stigma sensitivity) | To assess the association between sources of stigma and health care avoidance among gay and bisexual men, as well as the role of heightened stigma sensitivity |
| Burns et al. 2023 | United States | 322 audience members | HIV | No | N/A | N/A | To explore the impact of As Much As I Can, an immersive theatre production, on HIV-related stigma behaviors |
| Caceres et al. 2021 | United States | 619 sexual minority women | Sexual minority status | Yes | Hatzenbuehler & Link 2014 | Minority stress | To gain greater understanding of the associations of past-year discrimination and the main attributions or reasons for discrimination with smoking outcomes in sexual minority women |
| Cain et al. 2017 | United States | 1,071 gay and bisexual men | Sexual minority status | No | N/A | Minority stress | To examine associations between population density and depressive symptoms and the role of internalized homonegativity and social support as potential mediators |
| Chong & Chan 2023 | Hong Kong | 505 lesbian, gay, and bisexual adults | Sexual minority status | Only examples | N/A | Minority stress | To examine how two self-compassion components were linked to life satisfaction via proximal stressors and whether the self-compassion dimensions moderated the links between proximal stressors and life satisfaction |
| Coelho et al. 2016 | Brazil | 2,224 people living with HIV | Sexual minority status | No | Hatzenbuehler et al. 2014 (retracted)  Pachankis et al. 2015 | N/A | To determine differences in mortality among women, heterosexual men, and men who have sex with men living with HIV in Rio de Janeiro, Brazil |
| Crockett, Rice & Turan 2018 | United States | 202 people living with HIV | HIV  Race/ethnicity  Sexual minority status | No | N/A | Intersectionality  Other (Avoidance coping) | To explore the psychosocial context of tobacco use in people living with HIV, examining avoidance coping as a mediator in the relationship between multiple forms of discrimination and tobacco use |
| Currin & Hubach 2017 | United States | 148 men who have sex with men | Sexual minority status | No | Hatzenbuehler et al. 2014 (retracted)  Hatzenbuehler 2009 | Intersectionality  Minority stress | To determine what predicts a man who identifies as a man who has sex with men living in a conservative state to inform his provider of his sexual behaviors and/or his sexual orientation |
| de Filippis et al. 2022 | Italy | 120 people with bipolar disorder | Mental health | Yes | Hatzenbuehler & Link 2014  Other (Mitchell et al. 2021) | Other (Mitchell et al.'s [2021] "long arm of oppression") | To assess the correlation between self-stigma, dissociative symptoms, and quality of life in a clinical sample of patients with bipolar disorder, and to test if the dissociative symptomatology may be related to a specific subthreshold of internalized stigma and/or to quality of life |
| Drabble et al. 2021 | United States | 446 sexual minority cisgender women | Sexual minority status | Yes | Hatzenbuehler & Link 2014 | Minority stress | To assess the psychometric properties of measures developed to assess the psychosocial impacts of legalized same-sex marriage on the lives of sexual minority cisgender women |
| Drabble et al. 2021 | United States | 446 sexual minority women | Sexual minority status | Yes | Hatzenbuehler & Link 2014 | Minority stress | To examine how perceptions of the impact of legalized same-sex marriage among sexual minority women may 1) differ by demographic characteristics and 2) predict alcohol use disorder, depression, and self-perceived health |
| Eisenberg et al. 2020 | United States | 2454 cisgender sexual minority adolescent students in 81 communities | Sexual minority status | No | Hatzenbuehler 2017 (JCCAP) | Minority stress  Social ecological model | To examine three hypotheses: among LGBQ adolescents, a) living in a more supportive community, b) attending a school with more resources and supports, and c) attending a school with a greater proportion of LGBQ peers will each be inversely associated with substance use behaviors |
| Everett et al. 2022 | United States | Sample sizes for birth weight, low birth weight, and preterm birth were 11,058, 11,320, and 11,414 births, respectively; all births were from individuals who reported "female" sex at beginning of study | Gender  Sexual minority status | No | N/A | Other (Structural sexism) | To create a state-level index of heteropatriarchy and examine its relationship to birth outcomes |
| Figueroa & Zoccola 2016 | United States | 277 sexual minority adults | Sexual minority status | Yes | Hatzenbuehler et al. 2014 (retracted) | Minority stress | To examine the effects of discrimination on the health of sexual minority people and how these may differ based on source of discrimination and the mediating role of perceived stress reactivity |
| Fish et al. 2019 | United States | 404,583 ninth- to 12th-grade students | Sexual minority status | No | Hatzenbuehler, Jun, Corliss & Austin 2014 | N/A | To examine rates of cigarette use and trends in cigarette use disparities between heterosexual youth and three subgroups of sexual minority youth |
| Friedman et al. 2017 | United States | 751 people, including 300 people who inject drugs, 260 high-risk heterosexual people who do not inject drugs, and 191 men who have sex with men who do not inject drugs | HIV  Sexual minority status  Substance use (injection drug use) | No | Link & Phelan 2001  Other (Entire special issue of *Social Science & Medicine* on structural stigma) | Other (Dignity [attacks on dignity]) | To use measures of dignity attacks and describe and measure their frequencies among three key populations (i.e., people who inject drugs, high-risk heterosexual people, and men who have sex with men) |
| Gilbert et al. 2022 | United States | 567 sexual and gender minority adults | Sexual minority status  Gender minority status | Yes | Hatzenbuehler & Link 2014 | N/A | To develop a detailed epidemiological profile of sexual and gender minority people in Iowa |
| Gower et al. 2019 | Multi-country (North America) | Communities around 634 schools | Sexual minority status  Gender minority status | No | N/A | Social ecological model | To describe the development of a new, theoretically and empirically grounded instrument to measure the supportiveness of the environments in which sexual minority youth live |
| Griffin & Fingerman 2018 | United States | 3,343 dating profiles of adults aged 60 and over | Age  Sexual minority status | Yes | Hatzenbuehler & Link 2014 | N/A | To examine older adults’ online dating profiles in a comparison of older adults seeking same- and cross-sex relationships as well as explore profile themes from individuals in geographic regions with different levels of structural support for sexual minority people |
| Hank, Neyer, & Thönnissen 2023 | Germany | 7,628 and 10,422 individuals ages 24 to 38 from two different surveys, including 96 and 325 individuals who reported to have had one or more same-sex relationships, respectively | Sexual minority status | Yes | Hatzenbuehler 2016 | Minority stress | To assess disparities in subjective well-being by sexual orientation before and after legalization of same-sex marriage in Germany |
| Hatzenbuehler et al. 2017 | United States | 243,996 people from 31 states in total sample, 19,961 of whom are Latinx people | Race/ethnicity | No | N/A | N/A | To examine associations between the state-level policy climate related to immigration and mental health outcomes among Latinx people |
| Hirsch et al. 2019 | United States | 913 people who lived in collegiate housing | Mental health | No | Hatzenbuehler 2016 | N/A | To examine the interrelationship between established risk factors for suicidal behavior, specifically testing depressive symptoms as a mediator of the relation between stress and suicidal behavior, along with the moderating effect of mental health stigma |
| Horne et al. 2022 | United States | 523 transgender, nonbinary, and gender-diverse people as well as cisgender lesbian, gay, bisexual, and queer people | Sexual minority status  Gender minority status | Yes | Hatzenbuehler & Link 2014 | Intersectionality  Minority stress | To explore structural stigma as a contextual factor in the mental health experiences of transgender, nonbinary, and gender-diverse people as well as cisgender lesbian, gay, bisexual, and queer people in the face of a referendum intended to remove rights to public bathrooms and other accommodations based on gender identity |
| Hossain et al. 2021 | Bangladesh | 1056 adults | COVID-19 | No | N/A | N/A | To determine the level of stigmatized attitudes related to COVID-19 and identify the correlates of the stigmatized attitudes among the adult population in Bangladesh |
| Huang & Liang 2022 | Taiwan | 863 cisgender men, including 731 gay men and 132 bisexual men | Sexual minority status | Yes | Hatzenbuehler & Link 2014 | N/A | To examine the short-term effects associated with the legalization of same-sex marriage for gay and bisexual men in Taiwan |
| Huang, Chan, & Cui 2020 | Taiwan | 1,381 Taiwanese gay and bisexual men | Sexual minority status | No | Hatzenbuehler 2016 | Minority stress  Other (Filial piety)  Other (Relational cultural theory) | To test a mediation model in which internalized homonegativity served as a mediator between filial piety and depressive symptoms among Taiwanese gay and bisexual men |
| Hutzler et al. 2016 | United States | Two studies; one with a sample of 100 adults and the other with a sample of 196 adults | Polyamory | Yes | Corrigan, Watson, Heyrman et al. 2005  Link & Phelan 2001 | Other multiple frameworks (Contact hypothesis and value self-confrontation theory) | To examine public awareness of polyamory and to identify traits that predict an individual's attitudes toward polyamory |
| Költő et al. 2023 | Ireland | 6,242 adolescents aged 12 to 19, including 545 sexual minority individuals, 1,355 individuals with disability/chronic condition, 890 individuals who were first-generation immigrants, and 127 Traveller individuals | Race/ethnicity  Sexual minority status  Disability | Yes | Other (Almeida et al. 2009; Heary, Hennessy, & Swords 2014) | N/A | To present the rates of perceived discrimination on various grounds among minority and non-minority adolescents |
| Koziara et al. 2022 | Poland | 518 people, including 245 sexually diverse cisgender men, 175 sexually diverse cisgender women, and 98 transgender and gender diverse persons | Sexual minority status, gender minority status | No | Hatzenbuehler 2016 | Minority stress | To explore the associations between age and selected health-related constructs including resilience and mental health indicators such as self-esteem and depression, as well as minority stress exposure, in Polish gender and sexually diverse persons |
| Kuerbis et al. 2017 | United States | 198 men who have sex with men not living with HIV who have or at risk of alcohol use disorder | Sexual minority status | No | Hatzenbuehler et al. 2014 (retracted) | Minority stress | To test whether drinking to cope with stress, loneliness, and gay community participation mediated the relationship between internalized heterosexism and behavioral health outcomes |
| Lamontagne et al. 2018 | Multi-continent | The index developed in the article was estimated for 158 countries | Sexual minority status | No | Hatzenbuehler & Link 2014  Hatzenbuehler 2017 (Handbook) | Other (Human rights) | To propose and validate a robust index to measure homophobia at the country level |
| Lapham & Martinson 2022 | United States | 2064 mothers who reported receipt of any public assistance benefit | Welfare receipt | No | Link & Phelan 2001 | Fundamental cause theory | To examine the association between two dimensions of welfare stigma and self-reported health among female public assistance participants with children and consider the moderating role of uneven state policies, income inequality, and negative public welfare attitudes |
| Law et al. 2021 | Canada | 137 adults who are general psychiatry patients | Mental health | Yes | N/A | Other (Modified labeling theory) | To examine the relationships between leverage strategies employed in psychiatric services and the experiences and impact of stigma |
| Lee et al. 2006 | Hong Kong | 480 people, including 320 people with schizophrenia and 160 people with diabetes mellitus | Mental health | No | Link & Phelan 2001  Pincus 1996 | N/A | To examine the personal experience and structural context of treatment-related stigma among Chinese patients with schizophrenia in Hong Kong |
| Lee et al. 2016 | United States | 577 adult men and women who are lesbian, gay, or bisexual | Sexual minority status | No | Hatzenbuehler & Link 2014 | Minority stress | To examine whether associations between discrimination, mental health, and substance use disorders among sexual minority adults patterned differentially by gender |
| Lee et al. 2020 | United States | 1,010 men who have sex with men | Immigrant status  Sexual minority status | No | Pachankis et al. 2015 | N/A | To examine how immigrant status is associated with sexual orientation disclosure among men who have sex with men, and to assess the impact of disclosure on HIV and sexually transmitted infection prevention outcomes stratified by nativity |
| Leluţiu-Weinberger et al. 2019 | Multi-country (Europe) | 2,087 sexual minority men not living with HIV | Sexual minority status | Only examples | Bos et al. 2013 | Minority stress | To assess whether stigma in the social contexts surrounding sexual minority men may place them at a higher risk for HIV contraction across six countries |
| Leluţiu-Weinberger, Clark, & Pachankis 2022 | Romania | 113 mental health providers | Sexual minority status  Gender minority status | Yes | Hatzenbuehler 2016 | Minority stress | To test the relative efficacy of a sexual and gender minority affirmative mental health training program delivered in-person and online in reducing provider bias toward sexual and gender minority people and improving competence related to sexual and gender minority people |
| Lewis et al. 2016 | United States | 867 lesbian women between the ages of 18 and 35 who were non-Hispanic White (n = 758) or Black (n = 109) | Race/ethnicity  Sexual minority status  Socioeconomic status | Yes | Hatzenbuehler & Link 2014 | Intersectionality  Minority stress  Other (Psychological mediation framework  Other (Reserve capacity model) | To test a conceptual model of hazardous drinking among Black and non-Hispanic White lesbian women underpinned by minority stress theory, the reserve capacity model, and intersectionality |
| Lewis et al. 2017 | United States | 1,048 lesbian women | Sexual minority status | Yes | Hatzenbuehler & Link 2014 | Minority stress  Other (Motivational model of alcohol use) | To test a conceptual model linking stigma-related stress and lesbian women’s alcohol use and related problems via social factors (i.e., social constraints and social isolation), depressive symptoms, and negative reinforcement drinking motives (i.e., coping and conformity) |
| Li et al. 2020 | United States | 70 Black and Latino men who have sex with men | Sexual minority status | Only examples | Hatzenbuehler 2014  Other (Herek 2016) | Minority stress  Other (Social genomic perspective) | To examine whether homophobic victimization is associated with expression of conserved transcriptional response to adversity profiles in Black and Latino men who have sex with men |
| Lin et al. 2022 | Taiwan | 736 gay and bisexual men aged 20 and older | Sexual minority status | No | Hatzenbuehler et al. 2014 (retracted)  Pachankis et al. 2021 | Minority stress | To examine whether the factor structure of a translated and culturally-adapted scale found among gay and bisexual men living in Taiwan is the same as that found among gay and bisexual men living in the United States |
| Lipperman-Kreda, Antin, & Hunt 2019 | United States | 227 sexual and gender minority adults who smoke or used to smoke | Race/ethnicity  Sexual minority status  Smoking  Socioeconomic status | No | Hatzenbuehler, Jun, Corliss, & Austin 2014 | Intersectionality | To investigate how intersections of being a racial minority (i.e., being African American) and economically disadvantaged (i.e., experiencing housing insecurity) may influence experiences with discrimination and perceptions of smoking-related stigma among sexual and gender minority adults who smoke or used to smoke |
| Luchachko, Hatzenbuehler, & Keyes 2014 | United States | 32,752 people ages 18 and older, including 8,245 non-Hispanic Black people and 24,507 non-Hispanic White people) | Race/ethnicity | No | Link & Phelan 2001  Link 2014 | Other multiple frameworks (Ecosocial model, structural racism, stigma power, and Bourdieu's misrecognition) | To use novel measures of structural racism to test the hypothesis that structural racism is a risk factor for myocardial infarction among Black people in the United States |
| Matson et al. 2022 | United States | 7562 patients with a positive alcohol screen, including 1337 transgender patients | Gender minority status | No | N/A | Minority stress | To evaluate the effect of the Veteran Health Administration Transgender Healthcare Directive on receipt of evidence-based alcohol-related care for transgender patients with unhealthy alcohol use |
| Mauro et al. 2021 | United States | 126,463 adults | Gender  Sexual minority status | Yes | Hatzenbuehler & Link 2014 | Intersectionality  Minority stress | To examine medical/non-medical prescription opioid use by intersecting sexual identity and gender and explored associations with medical cannabis laws |
| McKetta et al. 2023 | United States | 359 cisgender women who identified either as bisexual or lesbian | Sexual minority status | Only examples | Hatzenbuehler, 2014  Hatzenbuehler 2016  Hatzenbuehler et al., 2009  Other (Lattanner et al., 2021) | Minority stress | To examine the association between neighborhood deprivation and mental health and the potential moderating effects of minority stressors in a community-based sample of sexual minority women |
| Mereish & Miranda 2019 | United States | 20 sexual minority people ages 18 to 27 | Sexual minority status | No | N/A | Minority stress | To examine the effects of three novel mood inductions (i.e., stigma, general unpleasant, and neutral) on affect and alcohol craving among heavy drinking sexual minority young adults |
| Miller et al. 2014 | United States | 2,647 people, including 203 people living with HIV and 2,444 randomly selected residents of the same communities | HIV | Yes | Corrigan, Markowitz, & Watson 2004  Other (Phelan, Link, & Dovidio 2008) | Other (Social norms) | To examine the associations between community perceptions of norms for behaviors related to HIV transmission with the normative perceptions of people with HIV and with perceptions of stigmatization by people with HIV living in those same communities |
| Miller et al. 2016 | United States | 553 people, including 206 people living with HIV and 347 people who live in the same communities | HIV | Yes | Hatzenbuehler et al. 2014 (retracted) | Other (Social capital) | To examine 1) how community levels of implicit HIV prejudice are associated with the psychological and physical well-being of people with HIV living in those same communities, and 2) whether community motivation to control prejudice and/or explicit HIV prejudice moderates the relationship of implicit prejudice and well-being |
| Miller-Jacobs, Operario, & Hughto 2023 | United States | 107,558 adolescents residing in 14 states that used the 2019 Youth Risk Behavior Survey’s optional gender identity question in their surveys, including 1,790 transgender adolescents | Gender minority status | Yes | Other (Klein et al. 2021) | Minority stress  Other (Gender Minority Stress and Resilience Measure) | To explore associations between four state-level policies and six health outcomes in a sample of transgender adolescents |
| Moody et al. 2018 | United States | 1,071 gay and bisexual men not living with HIV | Sexual minority status | No | Hatzenbuehler, Jun, Corliss, & Austin 2014 | Minority stress | To examine the association between internalized homophobia and the two drug use outcomes of recent drug use and drug-related problems |
| Murray et al. 2023 | Multi-continent | 8,669 cisgender men who have sex with men | Sexual minority status | Only examples | Hatzenbuehler, 2014  Other (Altman et al. 2012) | N/A | To assess the factor structure and invariance in scales measuring sexual behavior stigma across two different domains (family and friends; other social/community contexts) among cisgender men who have sex with men living in nine countries located across three regions |
| Nardelli et al. 2020 | Italy | 120 White adults who identified as gay men | Sexual minority status | No | Hatzenbuehler 2014  Hatzenbuehler & Link 2014  Hatzenbuehler, McLaughlin, Keyes, & Hasin, 2010  Pachankis, Hatzenbuehler, & Starks, 2014 | Minority stress  Other (Herek's sexual stigma framework)  Other (Dissociation) | To empirically investigate the relationship between internalized sexual stigma and dissociation |
| Nelson et al. 2022 | Multi-continent | 10 HIV related interventions | HIV  Sexual minority status | No | N/A | N/A | To describe the experiences of implementing HIV prevention and care interventions with adolescent sexual minority men in a global perspective |
| Nocka et al. 2021 | United States | 221,468 individuals between the ages of 10 and 25, including 560 transgender/gender diverse individuals | Gender minority status | Only examples | Other (McDowell et al. 2020) | N/A | To describe preventive care use and access among transgender and gender diverse adolescents |
| Pachankis et al. 2015 | United States | 63 young gay and bisexual men | Sexual minority status | Yes | Hatzenbuehler 2014 | Minority stress | To test the preliminary efficacy of a transdiagnostic cognitive behavioral treatment adapted to improve depression, anxiety, and co-occurring health risks (i.e., alcohol use, sexual compulsivity, condomless sex) among young adult gay and bisexual men |
| Pachankis et al. 2020 | United States | 60 young adult sexual minority women | Sexual minority status  Gender minority status | No | N/A | Minority stress | To test the efficacy of a minority-stress-focused cognitive-behavioral treatment intended to improve sexual minority women's mental and behavioral health |
| Parmenter & Winter 2023 | United States | 618 people who self-identified as LGBTQ+ and were 18 years or older | Sexual minority status  Gender minority status | Only examples | Hatzenbuehler et al. 2009  Pachankis & Bränström 2018  Pachankis et al. 2021  Other (Price et al., 2023) | Minority stress | To examine if LGBTQ+ community inequity functioned as a distal stressor similar to cisheterosexist discrimination in directly influencing depression and anxiety, as well as indirectly through proximal stress (i.e., internalized minority stress) |
| Pellicane & Ciesla 2022 | United States | Over 1.8 million university students | Sexual minority status | Yes | Hatzenbuehler & Link 2014 | Minority stress | To determine whether disparities in depression, anxiety, and suicidality between cisgender sexual minority and heterosexual college students has decreased in the last two decades |
| Perales 2016 | Australia | 14,950 adults, including 216 gay/lesbian adults and 205 bisexual adults | Sexual minority status | No | N/A | Minority stress | To provide empirical evidence on how sexual identity influences a range of subjective well-being outcomes (e.g., mental health, life satisfaction, psychological distress and feelings of safety) and how its effects evolve over the lifecourse |
| Philbin et al. 2020 | United States | 126,463 adults | Sexual minority status | Only examples | Other (Lea, de Wit, & Reynolds 2014) | Minority stress | To describe: 1) the prevalence of medical prescription stimulant use, nonmedical prescription stimulant use, and illegal stimulant use by sexual identity and gender in a national sample of United States adults, and 2) differences in overlapping stimulant use and polysubstance use by sexual identity |
| Pitoňák, Kožený, & Čihák 2023 | Czechia | A general population sample of 1,841 people ages 15 to 92 and a sexual minority community sample of 1,788 people ages 15 to 71 | Sexual minority status | No | Other (Hatzenbuehler, Slopen, & McLaughlin 2014; Pachankis et al. 2017) | Minority stress | To compare the psychological distress between a general population sample and sexual minority community sample |
| Purtle et al. 2018 | United States | 475 state legislators | Mental health  Substance use (general drug use) | No | Corrigan, Watson, Heyrman et al. 2005  Hatzenbuehler 2016 | N/A | To 1) identify behavioral health (i.e., mental health and substance use) audience segments among state legislators, 2) identify legislator characteristics that are predictive of segment membership, and 3) determine whether segment membership is predictive of support for state behavioral health parity laws |
| Raifman et al. 2020 | United States | 917,633 people with data in Rhode Island's all payer claims database | Race/ethnicity  Sexual minority status | No | Hatzenbuehler & Link 2014  Hatzenbuehler 2014 | N/A | To develop and use an algorithm to identify and evaluate uptake and use of pre-exposure prophylaxis for HIV in a state all payer claims database |
| Robles et al. 2023 | United States | 612 Latino sexual minority cisgender men at least 18 years old who are partnered with a cisgender man | Race/ethnicity  Sexual minority status | Yes | Link & Phelan 2001  Pachankis, Hatzenbuehler, & Starks 2014 | N/A | To examine how state-level characteristics relate to social support and mental health outcomes among Latino sexual minority men |
| Saewyc et al. 2020 | Canada | 2,678 sexual minority students | Sexual minority status | No | Hatzenbuehler 2017 (Handbook) | Minority stress | To characterize LGBTQ-supportive environments and political climates, and examine their links to suicidal behavior among sexual minority adolescents |
| Samari et al. 2020 | United States | 18,945,795 singleton live births, including 191,121 born to women from banned countries | Immigrant status | No | N/A | N/A | To examine associations between the enactment of the 2017 ban on individuals from Muslim majority countries and preterm birth among women from those countries residing in the United States |
| Sarno, Smith, & Newcomb 2023 | United States | 932 cisgender men in relationships with cisgender men (466 dyads) | Sexual minority status | Only examples | N/A | Minority stress | To examine associations of minority stress with intimate partner aggression, as well as the moderating role of positive and negative communication, among male same-sex couples |
| Schuler et al. 2018 | United States | 67,354 adults ages 18–49 | Sexual minority status | Yes | Hatzenbuehler & Link 2014  Hatzenbuehler 2014 | Minority stress | To examine how substance use behavior and disorder disparities among sexual minority adults differ by age and gender |
| Seager van Dyk, Aldao, & Pachankis 2022 | United States | 168 lesbian, gay, and bisexual adults | Sexual minority status | Yes | Hatzenbuehler 2014 | Minority stress | To investigate whether minority stress is causally linked to reduced disclosure in sexual minorities, and whether emotion regulation can intervene to promote disclosure even following exposure to minority stress |
| Starks et al. 2023 | United States | 7,501 cisgender men aged 18 or older in relationships with cisgender men aged 18 or older | Sexual minority status | Only examples | Hatzenbuehler 2014 | Minority stress  Other (Rusbult’s [1980] investment model) | To conduct a comprehensive evaluation of the associations between mental health and relationship status (single vs. partnered) as well as relationship quality in the context of state-level LGBTQ+ supportive public policy |
| Taylor 2014 | United States | 70 university students | Gender | No | N/A | N/A | To assesses whether men who lose social influence exhibit more of a stress response than men who gain social influence |
| Trani et al. 2020 | South Africa | 1449 people who participated in a community-based rehabilitation program | Disability | Yes | Other (Pryor & Reeder 2015) | Other (Social model of disability) | To test the link between public and self-stigma associated with disability in the Afghan social context, the association between social exclusion and mental distress, and the mediating role of social exclusion on the association between being born with a disability and mental distress |
| Trani, Ballard, & Peña 2016 | Afghanistan | 387 adults, including 191 living with a disability and 196 without a disability | Disability | Yes | Other (Pryor & Reeder 2011) | N/A | To measure a mediating model between disability, stigma, depression and self-esteem using validated and reliable scales as well as examine whether discrimination and stigma accounts for the hypothesized relationship between disability, depression and anxiety |
| Wardecker et al. 2022 | United States | 3,421 adults over 25 years of age, including 3,323 heterosexual adults and 98 sexual minority adults, contributing 24,773 daily diary interviews) | Sexual minority status | Only examples | Hatzenbuehler 2016 | Minority stress | To understand whether people who are sexual minorities differ from people who are heterosexual in their exposure and reactivity to general, non-sexual minority-specific stressors (e.g., arguments/disagreements, job concerns) |
| Warren 2023 | United States | 30 people, including 15 people with mild cognitive impairment and 15 care partners | Dementia | No | Hatzenbuehler & Link 2014 | Other (Chronic stress response) | To examine the relationship between perceived stigma and perceived stress in persons with mild cognitive impairment and their care partners |
| Weeks, Renshaw, & Vinal 2021 | United States | 152 non-heterosexual adolescents | Sexual minority status | No | N/A | Intersectionality  Minority stress | To test the relative predictive value of minority stress measured at each of the three levels specified by the minority stress model: the overall (or global) level, the proximal vs. distal (or domain) level, and the specific stressor (or subdomain) level |
| Whiteman et al. 2021 | United States | 10,029 men who have sex with men | Sexual minority status | No | N/A | N/A | To determine the prevalence of four forms of sexual identity discrimination among men who have sex with men in 23 United States metropolitan statistical areas, and examine racial/ethnic and socioeconomic disparities in each form of discrimination |
| Woodford et al. 2018 | United States | 268 sexual minority college students | Sexual minority status | Yes | Hatzenbuehler & Link 2014 | Minority stress | To investigate the association between campus-based structural factors and the experiences and psychological well-being of cisgender sexual minority college students |
| Ylioja et al. 2018 | United States | 566 sexual minority university students | Sexual minority status | Only examples | Hatzenbuehler, Jun, Corliss, & Austin 2014  Pachankis, Hatzenbuehler & Starks 2014 | Minority stress  Other (Microaggressions) | To examine the relationship between experiencing frequent (chronic) interpersonal microaggressions related to sexual minority status on campus and smoking among sexual minority college students |
| Qualitative research (*n* = 33) | | | | | | | |
| Abboud et al. 2023 | Lebanon | 25 men who have sex with men who live in Lebanon; 12 who were displaced from Syria and 13 who are Lebanese | Sexual minority status  Displacement | Only examples | Hatzenbuehler 2016 | Intersectionality  Minority stress | To investigate the intersection of minority stress and forced migration/immigration stress and improve understanding of sexual orientation-related and displacement-related determinants of the mental health of men who have sex with men, including displaced Syrian men who have sex with men, in Lebanon |
| Altshuler et al. 2021 | United States | 20 cisgender women | Abortion | No | N/A | N/A | To understand the meaning people who have given birth and have had an abortion ascribe to being accompanied by partners, family members and friends during these reproductive experiences |
| Biradavolu et al., 2012 | India | Three rounds of qualitative interviews with sex workers (n = 22, n = 17, n = 16) | HIV  Sex work | No | Other (Scambler 2006) | N/A | To describe the strategies employed to address stigma within the larger context of an intervention’s mobilization efforts and inform ongoing implementation and future directions |
| Brown et al. 2022 | Australia | 90 peer staff living with HIV from 10 community and peer organizations | HIV | No | N/A | N/A | To understand how to tackle structural stigma via the Meaningful Involvement of People with HIV/AIDS, while highlighting the challenges in demonstrating peer leadership from people living with HIV |
| Campbell 2021 | United States | 30 Black cisgender gay and bisexual men living with HIV | HIV  Race/ethnicity  Sexual minority status | No | N/A | Intersectionality,  Other (Interaction theory of emotion work  Other (Healthism) | To explore the emotional aspects of HIV disclosure for Black gay and bisexual men living with HIV |
| D'Alonzo et al. 2023 | United States | 11 students | Immigrant status | Only examples | Hatzenbuehler 2016 | N/A | To: 1) design a program to train community researchers to address obesity among Mexican immigrant families, and 2) identify the key components of a successful program |
| Dimova et al. 2022 | United Kingdom | 14 LGBTQ+ people who had sought help from an alcohol service or alcohol peer support group | Sexual minority status | No | Pachankis & Bränström 2018  Pachankis et al., 2021 | Minority stress | To explore the experiences of LGBTQ+ people who had used alcohol services and peer support groups |
| Fauk et al. 2021 | Indonesia | 92 people living with HIV | HIV | No | Hatzenbuehler & Link 2014  Hatzenbuehler 2016 | Other (Earnshaw & Chaudoir’s HIV stigma framework) | To describe perceptions of drivers of HIV stigma and discrimination within families, communities, and healthcare settings |
| Felner et al. 2020 | United States | 59 sexual and gender minority young adults who met criteria for a probable substance use disorder | Sexual minority status  Gender minority status | No | N/A | Minority stress | To qualitatively examine how lesbian, gay, bisexual, transgender, and queer young adults with probable substance use disorders conceptualized their substance use vis-à-vis their sexual and gender minority identities |
| Franks et al. 2022 | Australia | 7 gender-affirming general practitioners | Gender minority status | No | N/A | Minority stress | To explore the barriers and enablers encountered by gender-affirming general practitioners when treating trans and gender diverse patients |
| Gaspar et al. 2021 | Canada | 24 sexual minority men | Sexual minority status | Yes | Hatzenbuehler & Link 2014 | Minority stress | To understand experiences of sexual minority men in Toronto when navigating mental health services in and beyond HIV programs, with a critical focus on the structural factors shaping these men's encounters with mental healthcare |
| Gonzalez, Pulice-Farrow, and Abreu 2022 | United States | 335 sexual and gender minority individuals | Sexual minority status  Gender minority status | Yes | Hatzenbuehler & Link 2014 | Minority stress | To explore strategies that LGBTQ people use to cope during the U.S. presidential administration of Donald Trump |
| Graham et al. 2016 | United States | 60 African American and Latina/o, structurally disadvantaged youth of diverse gender and sexual identities | Space | No | Corrigan, Watson, Gracia et al. 2005  Hatzenbuehler et al. 2014 (retracted) | Territorial stigmatization | To examine how young Detroit residents interpret and experience the symbolic qualities of their city, with particular focus on spatial stigma |
| Greaves et al. 2022 | Barbados | 15 medical educators | Gender minority status | No | N/A | N/A | To explore medical educators’ understanding of the term transgender and their attitudes and perspectives regarding both health system responsiveness to transgender needs and transgender curriculum in medical education |
| Grzanka et al. 2020 | United States | 20 sexual and gender minority people | Sexual minority status  Gender minority status | No | Hatzenbuehler 2016 | Minority stress | To understand sexual and gender minority people's experiences with mental health care and perceptions of a law allowing counselors and therapists to deny services to any client based on "sincerely held principles" |
| Hansen, Bourgois, & Drucker 2014 | United States | 127 patients, providers, and health system administrators as well as 30 members of social networks of low-income Puerto Rican families residing in East Harlem housing projects | Welfare receipt | No | N/A | Other multiple frameworks (“A variety of thinkers who have contributed to theoretical approaches of political economy and moral economy”) | To describe the subjective experience of structural stigma imposed by the increasing medicalization of public support for the poor through a diagnosis of permanent mental disability |
| Harvey, Keene, & Pachankis 2021 | United States | 20 gay or bisexual men who were formerly incarcerated | Sexual minority status | Only examples | Hatzenbuehler 2016 | Minority stress | To examine how gay and bisexual men navigate minority stress and how this navigation influences their psychosocial health before, during, and after incarceration |
| Horgan 2018 | Canada | Case study of one neighborhood that draws on archival research, participant observation and interviews with residents | Space | No | Other (Hansen et al. 2014) | Territorial stigmatization | To understand and extend upon territorial stigmatization and how it operates in a particular neighborhood |
| Ingraham & Hann 2022 | United States | 25 family planning clinic staff members | Gender minority status  Abortion | No | N/A | Other multiple frameworks (Stigma management and "dangertalk” conceptual model) | To examine if or how stigma of transgender care may impact providers’ experience of their work in family planning clinics |
| Kalfa, Branicki, & Brammer 2021 | Australia | 39 human resource managers | Mental health | Yes | Other (Sheehan, Nieweglowski, & Corrigan 2017) | N/A | To explore how organizations vary in relation to their accommodation of mental health conditions, and how different approaches to accommodating employees' mental health conditions influence stigmatization of mental health conditions |
| Keene et al. 2017 | United States | 29 sexual minority men | Sexual minority status | No | N/A | Minority stress | To examine how stigma contributes to patterns of mobility within and beyond small city spaces, and the potential health implications associated with these forms of mobility |
| Lucey & Grimm 2021 | Senegal | 25 adults | Use of local forest foods and medicines | Yes | Hatzenbuehler & Link 2014  Raghavan et al. 2008 | Other (Political ecology of health and disease) | To examine how diets and health in rural Kédougou, Senegal are influenced by increased access to globalized foodstuffs and stigmatization of local foods and medicines |
| McMillian-Bohler et al. 2023 | United States | 22 women living with HIV aged 36 to 62 years | HIV | No | Hatzenbuehler 2016 | Other (Adaptive Leadership Framework for Chronic Illness) | To explore stigma and disclosure among women living and aging with HIV |
| Philbin et al. 2023 | United States | 68 people, including 30 sexual and gender minority youth (i.e., ages 18-29) and 38 community stakeholders | Sexual minority status  Gender minority status | Yes | Hatzenbuehler & Pachankis 2016  Other (Philbin et al. 2021) | Other multiple frameworks (Bourdieusian thought and ecosocial theory) | To identify the pathways through which social policies affect SGMY health and well-being |
| Phillips et al. 2020 | Australia | 24 Asian-born sexual minority men | HIV  Race/ethnicity  Sexual minority status | Only examples | Pachankis & Bränström 2018 | Intersectionality  Minority stress | To explore HIV knowledge and prevention strategies used by Asian-born gay, bisexual, and other men who have sex with men who are newly arrived in Australia |
| Qasim et al. 2020 | United Kingdom | Around 130 people in the audience of a dramatic comedy performance as well as the performing artist | Mental health  Race/ethnicity | Yes | Hatzenbuehler 2016  Other (Huggett et al. 2018; Mora-Ríos & Bautista 2014) | Other multiple frameworks (Post-structural philosophy and epistemic power) | To increase understanding of the use of creative arts (i.e., a dramatic comedy performance) in raising awareness of stigma in the context of mental distress |
| Reber et al. 2022 | United States | 72 adults with physical disabilities | Age  Disability | Yes | Hatzenbuehler & Link 2014 | Other (Structural ableism) | To identify where adults with physical disabilities experience the attitudinal environment, the continuum of those attitudes, and how they impact emotional and psychological health and well-being |
| Reimer-Kirkham et al. 2022 | Tanzania | 62 people, including 26 mothers either living with albinism or having children living with albinism and 36 key informants | Albinism | No | N/A | Other multiple frameworks (Stangl et al.’s [2019] Health Stigma and Discrimination Framework and human rights) | To explore the resilience of mothers impacted by albinism, at the intersection of gender and religion, through a human rights lens |
| Riggle et al. 2018 | United States | 20 sexual minority women | Sexual minority status | No | N/A | N/A | To examine whether same-sex marriage legalization would impact the relationship of sexual minority women with their families of origin regarding their same-sex relationships |
| Rosenberg 2018 | Australia | 8 queer people | Sexual minority status  Gender minority status | No | N/A | Minority stress | To explore the nonlinearity of sexual self-discovery journeys of young queer people |
| Subramaniam et al. 2022 | Singapore | 30 young people (20-34 years old) | Mental health | Yes | Hatzenbuehler & Link 2014 | N/A | To understand and describe the meaning of work as well as the barriers and facilitators perceived by young people with mental health conditions for gaining and maintaining employment |
| Tyler 2015 | United States | 22 individuals, 11 parent-adult child dyads | Sexual minority status  Gender minority status | No | N/A | Other (Symbolic interactionism) | To explore the parent-child relational process before and after the child discloses their sexual orientation and/or gender identity to their parent |
| Winskell & Sabben 2016 | Multi-country (Africa) | 56 young people (ages 13-24) who submitted scripts | Sexual minority status | No | Hatzenbuehler & Link 2014 | Other multiple frameworks (Herek’s sexual stigma framework, symbolic violence, and social representations theory) | To describe perceptions of drivers of HIV stigma and discrimination within families, communities, and healthcare settings |
| Mixed or multiple methods (*n* = 9) | | | | | | | |
| Beach et al. 2019 | United States | 2,999 adults, including 33 men and 61 women who self-identified as bisexual | Sexual minority status | No | Hatzenbuehler et al. 2014 | N/A | To investigate bisexual people's perceptions of others' attitudes toward bisexual people and describe psychometric properties of a scale measuring attitudes toward bisexual people |
| Chiyomi et al. 2021 | South Africa | 219 incarcerated people living with HIV were surveyed; 30 people living with HIV were interviewed | HIV  Incarceration | No | Other (Turan et al. 2017) | Intersectionality | To investigate the prevalence and experience of stigma among incarcerated people living with HIV during roll-out of universal test and treat |
| Drabble et al. 2020 | United States | 418 sexual minority women and gender diverse individuals | Sexual minority status  Gender minority status | Yes | Hatzenbuehler & Link 2014 | Minority stress  Sexual stigma conceptual framework | To explore perceptions of the impact of marriage legalization in all U.S. states among sexual minority women and gender diverse individuals |
| Drabble et al. 2019 | United States | 969 sexual minority women, transgender, and gender non-conforming individuals; 605 of whom also provided data for the qualitative analysis | Sexual minority status  Gender minority status | Yes | Hatzenbuehler & Link 2014 | N/A | To explore how sexual minority women, transgender, and gender non-conforming individuals in different geographic regions experienced the outcome of the 2016 federal election in the United States |
| Hancock & Karaffa 2021 | United States | 573 veterinary students | Mental health | Yes | Hatzenbuehler & Link 2014 | Other multiple frameworks (Identity and agency in sociocultural groups and identity production in figured worlds) | To investigate veterinary students’ perceptions of barriers to seeking mental health services and potential mechanisms to promote their use |
| Livingston, Rossiter, & Verdun-Jones 2011 | Canada | 91 people who have received compulsory community-based treatment for mental illness | Mental health | No | Hatzenbuehler & Link 2014 | N/A | To examine and compare the level of self-stigma that was experienced by people who receive compulsory community-based services in the forensic and civil mental health systems |
| Metzl & Petty 2017 | United States | 85 university students | General structural stigma | No | Link, Castille, & Stuber 2008 | Structural competency | To evaluate the incorporation of structural competency concepts and skills into undergraduate courses and present on the use of a novel evaluation instrument |
| Rao et al. 2020 | India | 170 sexual and gender minority people | Sexual minority status  Gender minority status | Yes | N/A | N/A | To explore perceived consequences of Section 377 (i.e., a now-struck-down law that penalized same-sex sexual activity in India) among Indian sexual and gender minorities |
| Veldhuis et al. 2018 | United States | 741 sexual minority women and gender minority people | Sexual minority status  Gender minority status | Yes | Hatzenbuehler, Phelan, & Link 2013  Major, Dovidio, & Link 2018 | Minority stress | To examine how sexual minority women and gender minorities perceived the outcome of the election in relation to stigma-related concerns, perceptions, and expectations |

**References for the studies in these three tables are as follows:**

Abboud, S., Seal, D. W., Pachankis, J. E., Khoshnood, K., Khouri, D., Fouad, F. M., & Heimer, R. (2023). Experiences of stigma, mental health, and coping strategies in Lebanon among Lebanese and displaced Syrian men who have sex with men: A qualitative study. *Social Science & Medicine*, *335*, 116248. <https://doi.org/10.1016/j.socscimed.2023.116248>

Abi Hana, R., Arnous, M., Heim, E., Aeschlimann, A., Koschorke, M., Hamadeh, R. S., Thornicroft, G., Kohrt, B. A., Sijbrandij, M., Cuijpers, P., & El-Chammay, R. (2022). Mental health stigma at primary health care centres in Lebanon: Qualitative study. *International Journal of Mental Health Systems*, *16*(1), 23. <https://doi.org/10.1186/s13033-022-00533-y>

AboJabel, H., Argavan, E., Hassin-Baer, S., Inzelberg, R., & Werner, P. (2021). Exploring the perceptions and stigmatizing experiences of Israeli family caregivers of people with Parkinson’s disease. *Journal of Aging Studies*, *56*, 100910. <https://doi.org/10.1016/j.jaging.2020.100910>

Affuso, G., Picone, N., Costa, P. A., Bacchini, D., De Angelis, G., Esposito, C., & Aparicio-García, M. E. (2023). Minority stress and mental health in gay and lesbian youth: A comparative study of Italy and Spain. *American Journal of Orthopsychiatry*. <https://doi.org/10.1037/ort0000709>

Agénor, M., Pérez, A. E., Solazzo, A. L., Beccia, A. L., Samnaliev, M., Wu, J., Charlton, B. M., & Austin, S. B. (2022). Assessing Variations in Sexual Orientation- and Gender Identity-Related U.S. State Laws for Sexual and Gender Minority Health Research and Action, 1996–2016. *LGBT Health*, *9*(3), 207–216. <https://doi.org/10.1089/lgbt.2021.0157>

Almazan, A. N., King, D., Grasso, C., Cahill, S., Lattanner, M., Hatzenbuehler, M. L., & Keuroghlian, A. S. (2021). Sexual Orientation and Gender Identity Data Collection at US Health Centers: Impact of City-Level Structural Stigma in 2018. *American Journal of Public Health*, *111*(11), 2059–2063. <https://doi.org/10.2105/AJPH.2021.306414>

Altshuler, A. L., Ojanen-Goldsmith, A., Blumenthal, P. D., & Freedman, L. R. (2021). “Going through it together”: Being accompanied by loved ones during birth and abortion. *Social Science & Medicine*, *284*, 114234. <https://doi.org/10.1016/j.socscimed.2021.114234>

Angermeyer, M. C., Matschinger, H., Link, B. G., & Schomerus, G. (2014). Public attitudes regarding individual and structural discrimination: Two sides of the same coin? *Social Science & Medicine*, *103*, 60–66. <https://doi.org/10.1016/j.socscimed.2013.11.014>

Antoniou, T., Pritlove, C., Shearer, D., Tadrous, M., Shah, H., & Gomes, T. (2023). Accessing hepatitis C direct acting antivirals among people living with hepatitis C: A qualitative study. *International Journal for Equity in Health*, *22*(1), 112. <https://doi.org/10.1186/s12939-023-01924-4>

Arnhart, C., Neale, M., Collins, C., Chesher, T., Coffey, S., Rogers, T. C., Ottwell, R., & Hartwell, M. (2022). The Use of Person-Centered Language in Scientific Research Articles Focused on Autism. *Journal of Developmental & Behavioral Pediatrics*, *43*(2), 63–70. <https://doi.org/10.1097/DBP.0000000000001038>

Asadi, S., Cunningham, T. J., Morgan, T. A., Zimmerman, M., & Rodriguez-Seijas, C. (2023). Examining Measurement Invariance in the Personality Inventory for DSM-5 Brief Form Across Sexual and Gender Minority Status. *Assessment*, 107319112311764. <https://doi.org/10.1177/10731911231176449>

Askew, A. J., Randall, A., Alvarez, J. C., Elbe, C. I., Caravelli, N., Gonzales, M., Van Der Star, A., & Blashill, A. J. (2024). Examining the association of structural stigmas with body image-related outcomes among sexual minority individuals. *Body Image*, *48*, 101652. <https://doi.org/10.1016/j.bodyim.2023.101652>

Azagba, S., Ebling, T., & Hall, M. (2023). Health claims denial for alcohol intoxication: State laws and structural stigma. *Alcohol: Clinical and Experimental Research*, *47*(9), 1748–1755. <https://doi.org/10.1111/acer.15153>

Azagba, S., Shan, L., Hall, M., Wolfson, M., & Chaloupka, F. (2022). Repeal of state laws permitting denial of health claims resulting from alcohol impairment: Impact on treatment utilization. *International Journal of Drug Policy*, *100*, 103530. <https://doi.org/10.1016/j.drugpo.2021.103530>

Babbs, G., Wolfe, H. L., Ulrich, M. R., Raifman, J., & Lipson, S. K. (2023). Sexual and gender minority university students report distress due to discriminatory health care policies. *Stigma and Health*. <https://doi.org/10.1037/sah0000459>

Beach, L., Bartelt, E., Dodge, B., Bostwick, W., Schick, V., Fu, T.-C., Friedman, M. R., & Herbenick, D. (2019). Meta-Perceptions of Others’ Attitudes Toward Bisexual Men and Women Among a Nationally Representative Probability Sample. *Archives of Sexual Behavior*, *48*(1), 191–197. <https://doi.org/10.1007/s10508-018-1347-8>

Benintendi, A., Kosakowski, S., Lagisetty, P., Larochelle, M., Bohnert, A. S. B., & Bazzi, A. R. (2021). “I felt like I had a scarlet letter”: Recurring experiences of structural stigma surrounding opioid tapers among patients with chronic, non-cancer pain. *Drug and Alcohol Dependence*, *222*, 108664. <https://doi.org/10.1016/j.drugalcdep.2021.108664>

Bikos, L. J. (2021). “It’s all window dressing:” Canadian police officers’ perceptions of mental health stigma in their workplace. *Policing: An International Journal*, *44*(1), 63–76. <https://doi.org/10.1108/PIJPSM-07-2020-0126>

Biradavolu, M. R., Blankenship, K. M., Jena, A., & Dhungana, N. (2012). Structural stigma, sex work and HIV: Contradictions and lessons learnt from a community-led structural intervention in southern India. *Journal of Epidemiology and Community Health*, *66*(Suppl 2), ii95–ii99. <https://doi.org/10.1136/jech-2011-200508>

Blakey, J. M., & Gunn, A. (2018). The “ickiness factor”: Stigma as a barrier to exiting prostitution. *Journal of Offender Rehabilitation*, *57*(8), 538–561. <https://doi.org/10.1080/10509674.2018.1549177>

Blosnich, J. R., Marsiglio, M. C., Gao, S., Gordon, A. J., Shipherd, J. C., Kauth, M., Brown, G. R., & Fine, M. J. (2016). Mental Health of Transgender Veterans in US States With and Without Discrimination and Hate Crime Legal Protection. *American Journal of Public Health*, *106*(3), 534–540. <https://doi.org/10.2105/AJPH.2015.302981>

Boertien, D., & Vignoli, D. (2019). Legalizing Same-Sex Marriage Matters for the Subjective Well-being of Individuals in Same-Sex Unions. *Demography*, *56*(6), 2109–2121. <https://doi.org/10.1007/s13524-019-00822-1>

Böge, K., Zieger, A., Mungee, A., Tandon, A., Fuchs, L., Schomerus, G., Tam Ta, T., Dettling, M., Bajbouj, M., Angermeyer, M., & Hahn, E. (2018). Perceived stigmatization and discrimination of people with mental illness: A survey-based study of the general population in five metropolitan cities in India. *Indian Journal of Psychiatry*, *60*(1), 24. <https://doi.org/10.4103/psychiatry.IndianJPsychiatry_406_17>

Bränström, R., Fellman, D., & Pachankis, J. (2023). Structural Stigma and Sexual Minority Victimization Across 28 Countries: The Moderating Role of Gender, Gender Nonconformity, and Socioeconomic Status. *Journal of Interpersonal Violence*, *38*(3–4), 3563–3585. <https://doi.org/10.1177/08862605221108087>

Bränström, R., & Pachankis, J. E. (2018). Sexual orientation disparities in the co-occurrence of substance use and psychological distress: A national population-based study (2008–2015). *Social Psychiatry and Psychiatric Epidemiology*, *53*(4), 403–412. <https://doi.org/10.1007/s00127-018-1491-4>

Bränström, R., & Pachankis, J. E. (2021). Country-level structural stigma, identity concealment, and day-to-day discrimination as determinants of transgender people’s life satisfaction. *Social Psychiatry and Psychiatric Epidemiology*, *56*(9), 1537–1545. <https://doi.org/10.1007/s00127-021-02036-6>

Bränström, R., & Pachankis, J. E. (2023). Structural Stigma and 7-Year Improvement in Life Satisfaction among Diverse Groups of Sexual Minority Individuals: A Repeated Cross-Sectional Study across 28 Countries. *Social Problems*, spad029. <https://doi.org/10.1093/socpro/spad029>

Brener, L., Broady, T., Cama, E., & Treloar, C. (2022). The impact of sources of stigma on health care avoidance among gay and bisexual men in Australia. *AIDS Care*, 1–9. <https://doi.org/10.1080/09540121.2022.2057410>

Brooks, B. D., Kaniuka, A., Job, S. A., Hodgkinson, N., Kelliher Rabon, J., Williams, S. L., & Hirsch, J. K. (2022). Anticipated Sexual Minority Stress and Mental Health after the 2016 Presidential Election: Examining a Psychological Mediation Framework. *Journal of Homosexuality*, 1–24. <https://doi.org/10.1080/00918369.2022.2087481>

Broussard, K. (2020). The changing landscape of abortion care: Embodied experiences of structural stigma in the Republic of Ireland and Northern Ireland. *Social Science & Medicine*, *245*, 112686. <https://doi.org/10.1016/j.socscimed.2019.112686>

Brown, G., Reeders, D., Cogle, A., Allan, B., Howard, C., Rule, J., Chong, S., & Gleeson, D. (2022). Tackling structural stigma: A systems perspective. *Journal of the International AIDS Society*, *25*(S1). <https://doi.org/10.1002/jia2.25924>

Burns, P. A., Klukas, E., Sims-Gomillia, C., Omondi, A., Bender, M., & Poteat, T. (2024). As Much As I Can – Utilizing Immersive Theatre to Reduce HIV-Related Stigma and Discrimination Toward Black Sexual Minority Men. *Community Health Equity Research & Policy*, *44*(2), 151–163. <https://doi.org/10.1177/0272684X221115920>

Caceres, B. A., Hughes, T. L., Veldhuis, C. B., & Matthews, A. K. (2021). Past-year discrimination and cigarette smoking among sexual minority women: Investigating racial/ethnic and sexual identity differences. *Journal of Behavioral Medicine*, *44*(5), 726–739. <https://doi.org/10.1007/s10865-021-00217-x>

Cain, D. N., Mirzayi, C., Rendina, H. J., Ventuneac, A., Grov, C., & Parsons, J. T. (2017). Mediating Effects of Social Support and Internalized Homonegativity on the Association Between Population Density and Mental Health Among Gay and Bisexual Men. *LGBT Health*, *4*(5), 352–359. <https://doi.org/10.1089/lgbt.2017.0002>

Campbell, C. K. (2021). Emotions and emotion work before, during and after HIV disclosure among Black gay and bisexual men living with HIV. *Sociology of Health & Illness*, *43*(8), 1739–1753. <https://doi.org/10.1111/1467-9566.13372>

Campbell, C., & Mena, J. A. (2021). LGBTQ+ Structural Stigma and College Counseling Center Website Friendliness. *Journal of College Counseling*, *24*(3), 241–255. <https://doi.org/10.1002/jocc.12194>

Carlon, C. (2022). Making meaning of being bereaved by suicide: An everyday experience. *Mortality*, *27*(3), 255–271. <https://doi.org/10.1080/13576275.2020.1823353>

Cascalheira, C. J., Helminen, E. C., Shaw, T. J., & Scheer, J. R. (2022). Structural determinants of tailored behavioral health services for sexual and gender minorities in the United States, 2010 to 2020: A panel analysis. *BMC Public Health*, *22*(1), 1908. <https://doi.org/10.1186/s12889-022-14315-1>

Chanda, M. M., Perez-Brumer, A. G., Ortblad, K. F., Mwale, M., Chongo, S., Kamungoma, N., Kanchele, C., Fullem, A., Barresi, L., Bärnighausen, T., & Oldenburg, C. E. (2017). Barriers and Facilitators to HIV Testing Among Zambian Female Sex Workers in Three Transit Hubs. *AIDS Patient Care and STDs*, *31*(7), 290–296. <https://doi.org/10.1089/apc.2017.0016>

Chang, E.-S., Monin, J. K., Zelterman, D., & Levy, B. R. (2021). Impact of structural ageism on greater violence against older persons: A cross-national study of 56 countries. *BMJ Open*, *11*(5), e042580. <https://doi.org/10.1136/bmjopen-2020-042580>

Charlton, B. M., Hatzenbuehler, M. L., Jun, H., Sarda, V., Gordon, A. R., Raifman, J. R. G., & Austin, S. B. (2019). Structural stigma and sexual orientation‐related reproductive health disparities in a longitudinal cohort study of female adolescents. *Journal of Adolescence*, *74*(1), 183–187. <https://doi.org/10.1016/j.adolescence.2019.06.008>

Cheng, Z. H., Tu, M.-C., Li, V. A., Chang, R. W., & Yang, L. H. (2015). Experiences of Social and Structural Forms of Stigma Among Chinese Immigrant Consumers with Psychosis. *Journal of Immigrant and Minority Health*, *17*(6), 1723–1731. <https://doi.org/10.1007/s10903-015-0167-3>

Childs, E., Biello, K. B., Valente, P. K., Salhaney, P., Biancarelli, D. L., Olson, J., Earlywine, J. J., Marshall, B. D. L., & Bazzi, A. R. (2021). Implementing harm reduction in non-urban communities affected by opioids and polysubstance use: A qualitative study exploring challenges and mitigating strategies. *International Journal of Drug Policy*, *90*, 103080. <https://doi.org/10.1016/j.drugpo.2020.103080>

Chimoyi, L., Hoffmann, C. J., Hausler, H., Ndini, P., Rabothata, I., Daniels-Felix, D., Olivier, A. J., Fielding, K., Charalambous, S., & Chetty-Makkan, C. M. (2021). HIV-related stigma and uptake of antiretroviral treatment among incarcerated individuals living with HIV/AIDS in South African correctional settings: A mixed methods analysis. *PLOS ONE*, *16*(7), e0254975. <https://doi.org/10.1371/journal.pone.0254975>

Chong, E. S. K., & Chan, R. C. H. (2023). The Role of Self-Compassion in Minority Stress Processes and Life Satisfaction among Sexual Minorities in Hong Kong. *Mindfulness*, *14*(4), 784–796. <https://doi.org/10.1007/s12671-023-02106-7>

Clark, K. A., Salway, T., McConocha, E. M., & Pachankis, J. E. (2022). How do sexual and gender minority people acquire the capability for suicide? Voices from survivors of near-fatal suicide attempts. *SSM - Qualitative Research in Health*, *2*, 100044. <https://doi.org/10.1016/j.ssmqr.2022.100044>

Clough, B. A., Hill, M., Delaney, M., & Casey, L. M. (2020). Development of a measure of stigma towards occupational stress for mental health professionals. *Social Psychiatry and Psychiatric Epidemiology*, *55*(7), 941–951. <https://doi.org/10.1007/s00127-019-01820-9>

Clough, B. A., Ireland, M. J., & March, S. (2019). Development of the SOSS-D: A scale to measure stigma of occupational stress and burnout in medical doctors. *Journal of Mental Health*, *28*(1), 26–33. <https://doi.org/10.1080/09638237.2017.1370642>

Coelho, L., Grinsztejn, B., Castilho, J. L., De Boni, R., Quintana, M. S. B., Campos, D. P., Ribeiro, S. R., Pacheco, A. G., Veloso, V. G., & Luz, P. M. (2016). Mortality in HIV-infected women, heterosexual men, and men who have sex with men in Rio de Janeiro, Brazil: An observational cohort study. *The Lancet HIV*, *3*(10), e490–e498. <https://doi.org/10.1016/S2352-3018(16)30052-2>

Conley, D. L. (2021). The impact of structural stigma and other factors on state mental health legislative outcomes during the Trump administration. *Stigma and Health*, *6*(4), 476–486. <https://doi.org/10.1037/sah0000331>

Conley, D. L., & Baum, M. J. (2023). Predictors of structural stigma in state mental health legislation during the Trump administration. *Social Work in Mental Health*, *21*(1), 1–27. <https://doi.org/10.1080/15332985.2022.2062272>

Conway, A., Treloar, C., Crawford, S., Degenhardt, L., Dore, G. J., Farrell, M., Hayllar, J., Grebely, J., & Marshall, A. D. (2023). “You’ll come in and dose even in a global pandemic”: A qualitative study of adaptive opioid agonist treatment provision during the COVID-19 pandemic. *International Journal of Drug Policy*, *114*, 103998. <https://doi.org/10.1016/j.drugpo.2023.103998>

Corrigan, P. W., Watson, A. C., Gracia, G., Slopen, N., Rasinski, K., & Hall, L. L. (2005). Newspaper Stories as Measures of Structural Stigma. *Psychiatric Services*, *56*(5), 551–556. <https://doi.org/10.1176/appi.ps.56.5.551>

Corrigan, P. W., Watson, A. C., Heyrman, M. L., Warpinski, A., Gracia, G., Slopen, N., & Hall, L. L. (2005). Structural Stigma in State Legislation. *Psychiatric Services*, *56*(5), 557–563. <https://doi.org/10.1176/appi.ps.56.5.557>

Coulter, R. W., Henderson, E. R., Corey, S. L., Gagnon, K., Chugani, C. D., Egan, J. E., Murphy, C. E., Plenn, E. R., Routh, N., Roig, A., & Miller, E. (2022). Exploring How U.S. High School Staff Support, Protect, and Affirm Sexual and Gender Minority Youth: Methods and Lessons Learned from a Qualitative Interview Study. *International Journal of Qualitative Methods*, *21*, 160940692210931. <https://doi.org/10.1177/16094069221093132>

Crockett, K. B., Rice, W. S., & Turan, B. (2018). Associations Between Multiple Forms of Discrimination and Tobacco Use Among People Living With HIV: The Mediating Role of Avoidance Coping. *JAIDS Journal of Acquired Immune Deficiency Syndromes*, *78*(1), 9–15. <https://doi.org/10.1097/QAI.0000000000001636>

Croff, J. M., Hubach, R. D., Currin, J. M., & Frederick, A. F. (2017). Hidden Rainbows: Gay Bars as Safe Havens in a Socially Conservative Area Since the Pulse Nightclub Massacre. *Sexuality Research and Social Policy*, *14*(2), 233–240. <https://doi.org/10.1007/s13178-017-0273-1>

Currin, J. M., & Hubach, R. D. (2017). Predicting disclosure of MSM status to providers in a primarily socially conservative state. *Journal of Gay & Lesbian Social Services*, *29*(4), 445–452. <https://doi.org/10.1080/10538720.2018.1378145>

D’Alonzo, K. T., Jimenez, M. E., Ahmed, S., Vivar, M., Vivar, I., & Vivar, L. (2023). Use of Adolescent “Community Researchers” to Address Obesity Among Mexican Immigrant Families. *Hispanic Health Care International*, *21*(4), 179–183. <https://doi.org/10.1177/15404153231181700>

Davis, S., Wallace, B., Van Roode, T., & Hore, D. (2022). Substance Use Stigma and Community Drug Checking: A Qualitative Study Examining Barriers and Possible Responses. *International Journal of Environmental Research and Public Health*, *19*(23), 15978. <https://doi.org/10.3390/ijerph192315978>

de Filippis, R., Menculini, G., D’Angelo, M., Carbone, E. A., Tortorella, A., De Fazio, P., & Steardo, L. (2022). Internalized-stigma and dissociative experiences in bipolar disorder. *Frontiers in Psychiatry*, *13*, 953621. <https://doi.org/10.3389/fpsyt.2022.953621>

DeLuca, J. S., Clement, T. W., & Yanos, P. T. (2017). Does Individual Stigma Predict Mental Health Funding Attitudes? Toward an Understanding of Resource Allocation and Social Climate. *The Israel Journal of Psychiatry and Related Sciences*, *54*(1), 6–16.

Dimova, E. D., O’Brien, R., Elliott, L., Frankis, J., & Emslie, C. (2022). Exploring the experiences of alcohol service use among LGBTQ+ people in Scotland: A qualitative study. *International Journal of Drug Policy*, *109*, 103859. <https://doi.org/10.1016/j.drugpo.2022.103859>

Dobransky, K. M. (2020). Reassessing mental illness stigma in mental health care: Competing stigmas and risk containment. *Social Science & Medicine*, *249*, 112861. <https://doi.org/10.1016/j.socscimed.2020.112861>

Doyle, D. M., & Molix, L. (2015). Perceived Discrimination and Social Relationship Functioning among Sexual Minorities: Structural Stigma as a Moderating Factor: Structural Stigma and Relationships. *Analyses of Social Issues and Public Policy*, *15*(1), 357–381. <https://doi.org/10.1111/asap.12098>

Drabble, L. A., Mericle, A. A., Gómez, W., Klinger, J. L., Trocki, K. F., & Karriker-Jaffe, K. J. (2021). Differential Effects of State Policy Environments on Substance Use by Sexual Identity: Findings From the 2000–2015 National Alcohol Surveys. *Annals of LGBTQ Public and Population Health*, *2*(1), 53–71. <https://doi.org/10.1891/LGBTQ-2020-0029>

Drabble, L. A., Mericle, A. A., Munroe, C., Wootton, A. R., Trocki, K. F., & Hughes, T. L. (2022). Examining Perceived Effects of Same-Sex Marriage Legalization Among Sexual Minority Women: Identifying Demographic Differences and Factors Related to Alcohol Use Disorder, Depression, and Self-Perceived Health. *Sexuality Research and Social Policy*, *19*(3), 1285–1299. <https://doi.org/10.1007/s13178-021-00639-x>

Drabble, L. A., Mericle, A. A., Wootton, A. R., Munroe, C., Li, L., Trocki, K. F., & Hughes, T. (2021). Measuring the Impact of Legal Recognition of Same-Sex Marriage among Sexual Minority Women. *Journal of GLBT Family Studies*, *17*(4), 371–392. <https://doi.org/10.1080/1550428X.2021.1935382>

Drabble, L. A., Veldhuis, C. B., Wootton, A., Riggle, E. D. B., & Hughes, T. L. (2019). Mapping the Landscape of Support and Safety Among Sexual Minority Women and Gender Non-conforming Individuals: Perceptions After the 2016 US Presidential Election. *Sexuality Research and Social Policy*, *16*(4), 488–500. <https://doi.org/10.1007/s13178-018-0349-6>

Drabble, L. A., Wootton, A. R., Veldhuis, C. B., Perry, E., Riggle, E. D. B., Trocki, K. F., & Hughes, T. L. (2020). It’s complicated: The impact of marriage legalization among sexual minority women and gender diverse individuals in the United States. *Psychology of Sexual Orientation and Gender Diversity*, *7*(4), 396–406. <https://doi.org/10.1037/sgd0000375>

Earnshaw, V. A., Cox, J., Wong, P. L., Saifi, R., Walters, S., Azwa, I., Omar, S. F. S., Collier, Z. K., Hassan, A. A., Lim, S. H., Wickersham, J., Haddad, M. S., & Kamarulzaman, A. (2023). “I want the doctors to know that I am as bright as a candle”: Experiences with and Hopes for Doctor Interactions Among Malaysian Key Populations and People Living with HIV. *AIDS and Behavior*, *27*(7), 2103–2112. <https://doi.org/10.1007/s10461-022-03942-9>

Eisenberg, M. E., Erickson, D. J., Gower, A. L., Kne, L., Watson, R. J., Corliss, H. L., & Saewyc, E. M. (2020). Supportive Community Resources Are Associated with Lower Risk of Substance Use among Lesbian, Gay, Bisexual, and Questioning Adolescents in Minnesota. *Journal of Youth and Adolescence*, *49*(4), 836–848. <https://doi.org/10.1007/s10964-019-01100-4>

Elkhalid, A., Morrow, E., & Leong, T. (2023). “Do you need a green card or something?” Romantic relationships, citizenship, and stigmatizing communication. *Communication Monographs*, *90*(4), 477–498. <https://doi.org/10.1080/03637751.2023.2228381>

Everett, B. G., Limburg, A., Homan, P., & Philbin, M. M. (2022). Structural Heteropatriarchy and Birth Outcomes in the United States. *Demography*, *59*(1), 89–110. <https://doi.org/10.1215/00703370-9606030>

Everett, B. G., Limburg, A., McKetta, S., & Hatzenbuehler, M. L. (2022). State-Level Regulations Regarding the Protection of Sexual Minorities and Birth Outcomes: Results From a Population-Based Cohort Study. *Psychosomatic Medicine*, *84*(6), 658–668. <https://doi.org/10.1097/PSY.0000000000001092>

Fabbre, V. D., & Gaveras, E. (2020). The manifestation of multilevel stigma in the lived experiences of transgender and gender nonconforming older adults. *American Journal of Orthopsychiatry*, *90*(3), 350–360. <https://doi.org/10.1037/ort0000440>

Falck, F., & Bränström, R. (2023). The significance of structural stigma towards transgender people in health care encounters across Europe: Health care access, gender identity disclosure, and discrimination in health care as a function of national legislation and public attitudes. *BMC Public Health*, *23*(1), 1031. <https://doi.org/10.1186/s12889-023-15856-9>

Fauk, N. K., Hawke, K., Mwanri, L., & Ward, P. R. (2021). Stigma and Discrimination towards People Living with HIV in the Context of Families, Communities, and Healthcare Settings: A Qualitative Study in Indonesia. *International Journal of Environmental Research and Public Health*, *18*(10), 5424. <https://doi.org/10.3390/ijerph18105424>

Favre, S., Bajwa, N. M., Dominicé Dao, M., Audétat Voirol, M.-C., Nendaz, M., Junod Perron, N., Perneger, T., & Richard-Lepouriel, H. (2023). Association between burnout and stigma in physicians. *PLOS ONE*, *18*(4), e0283556. <https://doi.org/10.1371/journal.pone.0283556>

Felner, J. K., Wisdom, J. P., Williams, T., Katuska, L., Haley, S. J., Jun, H.-J., & Corliss, H. L. (2020). Stress, Coping, and Context: Examining Substance Use Among LGBTQ Young Adults With Probable Substance Use Disorders. *Psychiatric Services*, *71*(2), 112–120. <https://doi.org/10.1176/appi.ps.201900029>

Figueroa, W. S., & Zoccola, P. M. (2016). Sources of Discrimination and Their Associations With Health in Sexual Minority Adults. *Journal of Homosexuality*, *63*(6), 743–763. <https://doi.org/10.1080/00918369.2015.1112193>

Fish, J. N., Turner, B., Phillips, G., & Russell, S. T. (2019). Cigarette Smoking Disparities Between Sexual Minority and Heterosexual Youth. *Pediatrics*, *143*(4), e20181671. <https://doi.org/10.1542/peds.2018-1671>

Flentje, A., Clark, K. D., Cicero, E., Capriotti, M. R., Lubensky, M. E., Sauceda, J., Neilands, T. B., Lunn, M. R., & Obedin-Maliver, J. (2022). Minority Stress, Structural Stigma, and Physical Health Among Sexual and Gender Minority Individuals: Examining the Relative Strength of the Relationships. *Annals of Behavioral Medicine*, *56*(6), 573–591. <https://doi.org/10.1093/abm/kaab051>

Franks, N., Mullens, A. B., Aitken, S., & Brömdal, A. (2022). Fostering Gender-IQ: Barriers and Enablers to Gender-affirming Behavior Amongst an Australian General Practitioner Cohort. *Journal of Homosexuality*, 1–24. <https://doi.org/10.1080/00918369.2022.2092804>

Fredrick, E. G., Mann, A. K., Brooks, B. D., & Hirsch, J. K. (2022). Anticipated to Enacted: Structural Stigma Against Sexual and Gender Minorities Following the 2016 Presidential Election. *Sexuality Research and Social Policy*, *19*(1), 345–354. <https://doi.org/10.1007/s13178-021-00547-0>

Frey, J. J., Hall, W. J., Goldbach, J. T., & Lanier, P. (2021). “Here in the Bible Belt, It’s Predominantly Negative”: Sexual Identity Stigma in the American South, 50 Years After Stonewall. *Frontiers in Psychology*, *12*, 804064. <https://doi.org/10.3389/fpsyg.2021.804064>

Friedman, S. R., Pouget, E. R., Sandoval, M., Rossi, D., Mateu-Gelabert, P., Nikolopoulos, G. K., Schneider, J. A., Smyrnov, P., & Stall, R. D. (2017). Interpersonal Attacks on the Dignity of Members of HIV Key Populations: A Descriptive and Exploratory Study. *AIDS and Behavior*, *21*(9), 2561–2578. <https://doi.org/10.1007/s10461-016-1578-4>

Frost, D. M. (2020). Hostile and harmful: Structural stigma and minority stress explain increased anxiety among migrants living in the United Kingdom after the Brexit referendum. *Journal of Consulting and Clinical Psychology*, *88*(1), 75–81. <https://doi.org/10.1037/ccp0000458>

Füllgrabe, D., & Smith, D. S. (2023). “Monogamy? In this Economy?”: Stigma and Resilience in Consensual Non-Monogamous Relationships. *Sexuality & Culture*, *27*(5), 1955–1976. <https://doi.org/10.1007/s12119-023-10099-7>

Gagnon, M. (2015). Re-thinking HIV-Related Stigma in Health Care Settings: A Qualitative Study. *Journal of the Association of Nurses in AIDS Care*, *26*(6), 703–719. <https://doi.org/10.1016/j.jana.2015.07.005>

Gaspar, M., Marshall, Z., Rodrigues, R., Adam, B. D., Brennan, D. J., Hart, T. A., & Grace, D. (2021). Mental health and structural harm: A qualitative study of sexual minority men’s experiences of mental healthcare in Toronto, Canada. *Culture, Health & Sexuality*, *23*(1), 98–114. <https://doi.org/10.1080/13691058.2019.1692074>

Gilbert, P. A., Lee, A. A., Pass, L., Lappin, L., Thompson, L., Sittig, K. W., Baker, E., & Hoffman-Zinnel, D. (2020). Queer in the Heartland: Cancer Risks, Screenings, and Diagnoses among Sexual and Gender Minorities in Iowa. *Journal of Homosexuality*, 1–17. <https://doi.org/10.1080/00918369.2020.1826832>

Gonzalez, K. A., Pulice-Farrow, L., & Abreu, R. L. (2022). “In the Voices of People Like Me”: LGBTQ Coping During Trump’s Administration. *The Counseling Psychologist*, *50*(2), 212–240. <https://doi.org/10.1177/00110000211057199>

Gordon, J. H., Tran, K. T., Visoki, E., Argabright, S. T., DiDomenico, G. E., Saiegh, E., Hoffman, K. W., Erez, G., & Barzilay, R. (2023). The Role of Individual Discrimination and Structural Stigma in the Mental Health of Sexual Minority Youth. *Journal of the American Academy of Child & Adolescent Psychiatry*, S0890856723003751. <https://doi.org/10.1016/j.jaac.2023.05.033>

Górska, P., Bilewicz, M., & Winiewski, M. (2017). Invisible to the state. Institutional sexual stigma and collective action of LGB individuals in five East European countries. *Group Processes & Intergroup Relations*, *20*(3), 367–381. <https://doi.org/10.1177/1368430216684646>

Górska, P., van Zomeren, M., & Bilewicz, M. (2017). Intergroup Contact as the Missing Link Between LGB Rights and Sexual Prejudice. *Social Psychology*, *48*(6), 321–334. <https://doi.org/10.1027/1864-9335/a000313>

Gower, A. L., Saewyc, E. M., Corliss, H. L., Kne, L., Erickson, D. J., & Eisenberg, M. E. (2019). The LGBTQ Supportive Environments Inventory: Methods for quantifying supportive environments for LGBTQ youths. *Journal of Gay & Lesbian Social Services*, *31*(3), 314–331. <https://doi.org/10.1080/10538720.2019.1616023>

Grace, D., Jollimore, J., MacPherson, P., Strang, M. J. P., & Tan, D. H. S. (2018). The Pre-Exposure Prophylaxis-Stigma Paradox: Learning from Canada’s First Wave of PrEP Users. *AIDS Patient Care and STDs*, *32*(1), 24–30. <https://doi.org/10.1089/apc.2017.0153>

Graham, L. F., Padilla, M. B., Lopez, W. D., Stern, A. M., Peterson, J., & Keene, D. E. (2016). Spatial Stigma and Health in Postindustrial Detroit. *International Quarterly of Community Health Education*, *36*(2), 105–113. <https://doi.org/10.1177/0272684X15627800>

Gray, S., Bartels, S. A., Lee, S., & Stuart, H. (2021). A cross-sectional study of community perceptions of stigmatization amongst women affected by UN-peacekeeper perpetrated sexual exploitation and abuse. *BMC Public Health*, *21*(1), 2295. <https://doi.org/10.1186/s12889-021-12221-6>

Greaves, N., Emmanuel, M. K., Harvey, A., Harewood, H., Gromer, J., Majumder, Md. A. A., & Campbell, M. H. (2022). Medical Educators’ Perceptions and Experiences of Transgender Curriculum in Barbados. *Transgender Health*, trgh.2022.0027. <https://doi.org/10.1089/trgh.2022.0027>

Greene, N., Johnson, R. M., Rosen, J., German, D., & Cohen, J. E. (2021). Exploring the relationship between the alcohol policy environment and nondiscrimination laws: Implications for binge drinking disparities among LGB adults in the United States. *Drug and Alcohol Dependence*, *225*, 108749. <https://doi.org/10.1016/j.drugalcdep.2021.108749>

Griffin, E. M., & Fingerman, K. L. (2018). Online Dating Profile Content of Older Adults Seeking Same- and Cross-Sex Relationships. *Journal of GLBT Family Studies*, *14*(5), 446–466. <https://doi.org/10.1080/1550428X.2017.1393362>

Grzanka, P. R., DeVore, E. N., Frantell, K. A., Miles, J. R., & Spengler, E. S. (2020). Conscience clauses and sexual and gender minority mental health care: A case study. *Journal of Counseling Psychology*, *67*(5), 551–567. <https://doi.org/10.1037/cou0000396>

Gunasekaran, S., Tan, G. T. H., Shahwan, S., Goh, C. M. J., Ong, W. J., & Subramaniam, M. (2022). The perspectives of healthcare professionals in mental health settings on stigma and recovery—A qualitative inquiry. *BMC Health Services Research*, *22*(1), 888. <https://doi.org/10.1186/s12913-022-08248-z>

Hancock, T. S., & Karaffa, K. M. (2021). “Obligated to Keep Things Under Control”: Sociocultural Barriers to Seeking Mental Health Services Among Veterinary Medical Students. *Journal of Veterinary Medical Education*, e20210069. <https://doi.org/10.3138/jvme-2021-0069>

Hank, K., Neyer, F. J., & Thönnissen, C. (2023). Disparities in Subjective Well-being by Sexual Orientation: Comparing Cohorts from pairfam’s (2008-09) and FReDA’s (2021) Baseline Waves. *Comparative Population Studies*, *48*. <https://doi.org/10.12765/CPoS-2023-09>

Hansen, H., Bourgois, P., & Drucker, E. (2014). Pathologizing poverty: New forms of diagnosis, disability, and structural stigma under welfare reform. *Social Science & Medicine*, *103*, 76–83. <https://doi.org/10.1016/j.socscimed.2013.06.033>

Harvey, T. D., Keene, D. E., & Pachankis, J. E. (2021). Minority stress, psychosocial health, and survival among gay and bisexual men before, during, and after incarceration. *Social Science & Medicine*, *272*, 113735. <https://doi.org/10.1016/j.socscimed.2021.113735>

Hatzenbuehler, M. L., Bellatorre, A., Lee, Y., Finch, B. K., Muennig, P., & Fiscella, K. (2014). RETRACTED: Structural stigma and all-cause mortality in sexual minority populations. *Social Science & Medicine*, *103*, 33–41. <https://doi.org/10.1016/j.socscimed.2013.06.005>

Hatzenbuehler, M. L., Bränström, R., & Pachankis, J. E. (2018). Societal-level explanations for reductions in sexual orientation mental health disparities: Results from a ten-year, population-based study in Sweden. *Stigma and Health*, *3*(1), 16–26. <https://doi.org/10.1037/sah0000066>

Hatzenbuehler, M. L., Flores, A. R., & Gates, G. J. (2017). Social Attitudes Regarding Same-Sex Marriage and LGBT Health Disparities: Results from a National Probability Sample: Same-Sex Marriage and LGBT Health. *Journal of Social Issues*, *73*(3), 508–528. <https://doi.org/10.1111/josi.12229>

Hatzenbuehler, M. L., Jun, H.-J., Corliss, H. L., & Austin, S. B. (2014). Structural Stigma and Cigarette Smoking in a Prospective Cohort Study of Sexual Minority and Heterosexual Youth. *Annals of Behavioral Medicine*, *47*(1), 48–56. <https://doi.org/10.1007/s12160-013-9548-9>

Hatzenbuehler, M. L., Jun, H.-J., Corliss, H. L., & Bryn Austin, S. (2015). Structural stigma and sexual orientation disparities in adolescent drug use. *Addictive Behaviors*, *46*, 14–18. <https://doi.org/10.1016/j.addbeh.2015.02.017>

Hatzenbuehler, M. L., & McLaughlin, K. A. (2014). Structural Stigma and Hypothalamic–Pituitary–Adrenocortical Axis Reactivity in Lesbian, Gay, and Bisexual Young Adults. *Annals of Behavioral Medicine*, *47*(1), 39–47. <https://doi.org/10.1007/s12160-013-9556-9>

Hatzenbuehler, M. L., Prins, S. J., Flake, M., Philbin, M., Frazer, M. S., Hagen, D., & Hirsch, J. (2017). Immigration policies and mental health morbidity among Latinos: A state-level analysis. *Social Science & Medicine*, *174*, 169–178. <https://doi.org/10.1016/j.socscimed.2016.11.040>

Hatzenbuehler, M. L., Rutherford, C., McKetta, S., Prins, S. J., & Keyes, K. M. (2020). Structural stigma and all-cause mortality among sexual minorities: Differences by sexual behavior? *Social Science & Medicine*, *244*, 112463. <https://doi.org/10.1016/j.socscimed.2019.112463>

Hatzenbuehler, M. L., Weissman, D. G., McKetta, S., Lattanner, M. R., Ford, J. V., Barch, D. M., & McLaughlin, K. A. (2022). Smaller Hippocampal Volume Among Black and Latinx Youth Living in High-Stigma Contexts. *Journal of the American Academy of Child & Adolescent Psychiatry*, *61*(6), 809–819. <https://doi.org/10.1016/j.jaac.2021.08.017>

Hirsch, J. K., Rabon, J. K., Reynolds, E. E., Barton, A. L., & Chang, E. C. (2019). Perceived stress and suicidal behaviors in college students: Conditional indirect effects of depressive symptoms and mental health stigma. *Stigma and Health*, *4*(1), 98–106. <https://doi.org/10.1037/sah0000125>

Hofer, M. S., & Savell, S. M. (2021). “There Was No Plan in Place to Get Us Help”: Strategies for Improving Mental Health Service Utilization Among Law Enforcement. *Journal of Police and Criminal Psychology*, *36*(3), 543–557. <https://doi.org/10.1007/s11896-021-09451-0>

Hollinsaid, N. L., Pachankis, J. E., Mair, P., & Hatzenbuehler, M. L. (2023). Incorporating macro-social contexts into emotion research: Longitudinal associations between structural stigma and emotion processes among gay and bisexual men. *Emotion*, *23*(6), 1796–1801. <https://doi.org/10.1037/emo0001198>

Hollinsaid, N. L., Price, M. A., & Hatzenbuehler, M. L. (2022). Transgender-Specific Adolescent Mental Health Provider Availability is Substantially Lower in States with More Restrictive Policies. *Journal of Clinical Child & Adolescent Psychology*, 1–12. <https://doi.org/10.1080/15374416.2022.2140433>

Horgan, M. (2018). Territorial Stigmatization and Territorial Destigmatization: A Cultural Sociology of Symbolic Strategy in the Gentrification of Parkdale (Toronto). *International Journal of Urban and Regional Research*, *42*(3), 500–516. <https://doi.org/10.1111/1468-2427.12645>

Horne, S. G., McGinley, M., Yel, N., & Maroney, M. R. (2022). The stench of bathroom bills and anti-transgender legislation: Anxiety and depression among transgender, nonbinary, and cisgender LGBQ people during a state referendum. *Journal of Counseling Psychology*, *69*(1), 1–13. <https://doi.org/10.1037/cou0000558>

Hossain, M. B., Alam, Md. Z., Islam, Md. S., Sultan, S., Faysal, Md. M., Rima, S., Hossain, Md. A., Mahmood, M. M., Kashfi, S. S., Mamun, A. A., Monia, H. T., & Shoma, S. S. (2021). COVID-19 public stigma in the context of government-based structural stigma: A cross-sectional online survey of adults in Bangladesh. *Stigma and Health*, *6*(2), 123–133. <https://doi.org/10.1037/sah0000305>

Huang, Y.-T., Chan, R. C. H., & Cui, L. (2020). Filial piety, internalized homonegativity, and depressive symptoms among Taiwanese gay and bisexual men: A mediation analysis. *American Journal of Orthopsychiatry*, *90*(3), 340–349. <https://doi.org/10.1037/ort0000439>

Huang, Y.-T., & Liang, Z. (2022). Effects of Same-Sex Marriage Legalization for Sexual Minority Men in Taiwan: Findings From a Prospective Study. *International Journal of Public Health*, *67*, 1604489. <https://doi.org/10.3389/ijph.2022.1604489>

Huang, Y.-T., Luo, H., Ko, N.-Y., & Yen, C.-F. (2020). Perceived Attitudes Toward Lesbian, Gay, and Bisexual (LGB) Issues and Mental Health Among Taiwanese LGB Adults: The Mediating Role of Self-Acceptance. *Archives of Sexual Behavior*, *49*(5), 1671–1682. <https://doi.org/10.1007/s10508-020-01686-y>

Hubach, R. D., Currin, J. M., Giano, Z., Meyers, H. J., DeBoy, K. R., Wheeler, D. L., & Croff, J. M. (2019). Experiences of Stigma by Gay and Bisexual Men in Rural Oklahoma. *Health Equity*, *3*(1), 231–237. <https://doi.org/10.1089/heq.2018.0095>

Huebner, B. M., Kras, K. R., & Pleggenkuhle, B. (2019). Structural discrimination and social stigma among individuals incarcerated for sexual offenses: Reentry across the rural–urban continuum. *Criminology*, *57*(4), 715–738. <https://doi.org/10.1111/1745-9125.12226>

Hughes, L. D., Gamarel, K. E., King, W. M., Goldenberg, T., Jaccard, J., & Geronimus, A. T. (2022). State-Level Policy Stigma and Non-Prescribed Hormones Use among Trans Populations in the United States: A Mediational Analysis of Insurance and Anticipated Stigma. *Annals of Behavioral Medicine*, *56*(6), 592–604. <https://doi.org/10.1093/abm/kaab063>

Hutzler, K. T., Giuliano, T. A., Herselman, J. R., & Johnson, S. M. (2016). Three’s a crowd: Public awareness and (mis)perceptions of polyamory. *Psychology & Sexuality*, *7*(2), 69–87. <https://doi.org/10.1080/19419899.2015.1004102>

Ingraham, N., & Hann, L. (2022). ‘Stigma R us’: Stigma management at the intersection of abortion care and transgender care in family planning clinics. *SSM - Qualitative Research in Health*, *2*, 100043. <https://doi.org/10.1016/j.ssmqr.2022.100043>

Iott, B. E., Loveluck, J., Benton, A., Golson, L., Kahle, E., Lam, J., Bauermeister, J. A., & Veinot, T. C. (2022). The impact of stigma on HIV testing decisions for gay, bisexual, queer and other men who have sex with men: A qualitative study. *BMC Public Health*, *22*(1), 471. <https://doi.org/10.1186/s12889-022-12761-5>

Jackson, S. D., Harvey, T. D., Watson, R. J., Pereira, K., & Clark, K. A. (2023). Multilevel stigma and depression among a national sample of Black and Latinx LGBTQ+ adolescents in the United States. *Journal of Psychopathology and Clinical Science*, *132*(5), 577–589. <https://doi.org/10.1037/abn0000841>

Judd, H., Yaugher, A. C., O’Shay, S., & Meier, C. L. (2023). Understanding stigma through the lived experiences of people with opioid use disorder. *Drug and Alcohol Dependence*, *249*, 110873. <https://doi.org/10.1016/j.drugalcdep.2023.110873>

Kalfa, S., Branicki, L., & Brammer, S. (2021). Organizational accommodation of employee mental health conditions and unintended stigma. *The International Journal of Human Resource Management*, *32*(15), 3190–3217. <https://doi.org/10.1080/09585192.2021.1910536>

Kcomt, L., Evans-Polce, R. J., Engstrom, C. W., Takahashi, J., Matthews, P. A., Veliz, P. T., West, B. T., & McCabe, S. E. (2024). Social Ecological Influences on Nicotine/Tobacco Use Among Gender-Varying and Gender-Stable Adolescents and Adults in the USA. *Annals of Behavioral Medicine*, *58*(1), 1–11. <https://doi.org/10.1093/abm/kaad066>

Keene, D. E., Eldahan, A. I., White Hughto, J. M., & Pachankis, J. E. (2017). ‘The big ole gay express’: Sexual minority stigma, mobility and health in the small city. *Culture, Health & Sexuality*, *19*(3), 381–394. <https://doi.org/10.1080/13691058.2016.1226386>

Kellogg, A. J., Hancock, D. W., Cho, G. Y., & Reid, A. E. (2023). Community-level age bias and older adult mortality. *Social Science & Medicine*, *317*, 115449. <https://doi.org/10.1016/j.socscimed.2022.115449>

Kim, E., Singh, S., Bommaraju, A., Norris, A. H., & Bessett, D. (2021). “We have to respect that option”: The abortion aversion complex in safety-net healthcare organizations. *Social Science & Medicine*, *291*, 114468. <https://doi.org/10.1016/j.socscimed.2021.114468>

Kim, Y. J., & Dawson, M. C. (2023). Sports Reforms and Coaches’ Spoiled Identities: An Analysis of Structural Stigma. *Sociology of Sport Journal*, 1–9. <https://doi.org/10.1123/ssj.2022-0216>

Költő, A., Gavin, A., Vaughan, E., Molcho, M., Kelly, C., & Nic Gabhainn, S. (2023). Perceived Discrimination Among Adolescents in Ireland. *Health Education & Behavior*, *50*(2), 193–198. <https://doi.org/10.1177/10901981221133301>

Koziara, K., Mijas, M. E., Galbarczyk, A., Wycisk, J., Pliczko, M. P., Krzych-Miłkowska, K., & Grabski, B. (2022). It gets better with age: Resilience, stigma, and mental health among lesbian, gay, bisexual, transgender and queer persons from Poland. *Frontiers in Psychology*, *13*, 958601. <https://doi.org/10.3389/fpsyg.2022.958601>

Krüsi, A., Kerr, T., Taylor, C., Rhodes, T., & Shannon, K. (2016). ‘They won’t change it back in their heads that we’re trash’: The intersection of sex work-related stigma and evolving policing strategies. *Sociology of Health & Illness*, *38*(7), 1137–1150. <https://doi.org/10.1111/1467-9566.12436>

Kuerbis, A., Mereish, E. H., Hayes, M., Davis, C. M., Shao, S., & Morgenstern, J. (2017). Testing Cross-Sectional and Prospective Mediators of Internalized Heterosexism on Heavy Drinking, Alcohol Problems, and Psychological Distress Among Heavy Drinking Men Who Have Sex With Men. *Journal of Studies on Alcohol and Drugs*, *78*(1), 113–123. <https://doi.org/10.15288/jsad.2017.78.113>

Lamontagne, E., d’Elbée, M., Ross, M. W., Carroll, A., Plessis, A. du, & Loures, L. (2018). A socioecological measurement of homophobia for all countries and its public health impact. *European Journal of Public Health*, *28*(5), 967–972. <https://doi.org/10.1093/eurpub/cky023>

Lampropoulos, D., Wolman, A., & Apostolidis, T. (2017). Analyzing the presentation and the stigma of schizophrenia in French newspapers. *Social Psychiatry and Psychiatric Epidemiology*, *52*(12), 1541–1547. <https://doi.org/10.1007/s00127-017-1455-0>

Lane, J. (2023). Working Through Stigma: A Constructivist Grounded Theory of Delivering Health Services to Diverse 2SLGBTQ Populations. *Qualitative Health Research*, *33*(7), 624–637. <https://doi.org/10.1177/10497323231167828>

Lanthier, S., Mason, R., Logie, C. H., Myers, T., & Du Mont, J. (2023). “Coming out of the closet about sexual assault”: Intersectional sexual assault stigma and (non) disclosure to formal support providers among survivors using Reddit. *Social Science & Medicine*, *328*, 115978. <https://doi.org/10.1016/j.socscimed.2023.115978>

Lapham, J., & Martinson, M. L. (2022). The intersection of welfare stigma, state contexts and health among mothers receiving public assistance benefits. *SSM - Population Health*, *18*, 101117. <https://doi.org/10.1016/j.ssmph.2022.101117>

Lattanner, M. R., Ford, J., Bo, N., Tu, W., Pachankis, J. E., Dodge, B., & Hatzenbuehler, M. L. (2021). A Contextual Approach to the Psychological Study of Identity Concealment: Examining Direct, Interactive, and Indirect Effects of Structural Stigma on Concealment Motivation Across Proximal and Distal Geographic Levels. *Psychological Science*, *32*(10), 1684–1696. <https://doi.org/10.1177/09567976211018624>

Lattanner, M. R., & Hatzenbuehler, M. L. (2023). Thwarted belonging needs: A mechanism prospectively linking multiple levels of stigma and interpersonal outcomes among sexual minorities. *Journal of Social Issues*, *79*(1), 410–445. <https://doi.org/10.1111/josi.12564>

Law, S. F., Sirotich, F., Sunderji, N., Simpson, A., & Nakhost, A. (2021). The relationship between clinician leverage, patient experiences, and and the impact of stigma: A study in academic and community outpatient psychiatry settings. *General Hospital Psychiatry*, *72*, 15–22. <https://doi.org/10.1016/j.genhosppsych.2021.06.008>

Layland, E. K., Bränström, R., Murchison, G. R., & Pachankis, J. E. (2023). Kept in the Closet: Structural Stigma and the Timing of Sexual Minority Developmental Milestones Across 28 European Countries. *Journal of Youth and Adolescence*, *52*(10), 2012–2030. <https://doi.org/10.1007/s10964-023-01818-2>

Lee, F., Butts, C. T., & Schneider, J. A. (2023). Measuring structural HIV stigma. *Social Networks*, *74*, 275–284. <https://doi.org/10.1016/j.socnet.2023.04.001>

Lee, J. H., Gamarel, K. E., Bryant, K. J., Zaller, N. D., & Operario, D. (2016). Discrimination, Mental Health, and Substance Use Disorders Among Sexual Minority Populations. *LGBT Health*, *3*(4), 258–265. <https://doi.org/10.1089/lgbt.2015.0135>

Lee, J. J., Katz, D. A., Glick, S. N., Moreno, C., & Kerani, R. P. (2020). Immigrant Status and Sexual Orientation Disclosure: Implications for HIV/STD Prevention Among Men Who Have Sex with Men in Seattle, Washington. *AIDS and Behavior*, *24*(10), 2819–2828. <https://doi.org/10.1007/s10461-020-02831-3>

Lee, S., Chiu, M. Y. L., Tsang, A., Chui, H., & Kleinman, A. (2006). Stigmatizing experience and structural discrimination associated with the treatment of schizophrenia in Hong Kong. *Social Science & Medicine*, *62*(7), 1685–1696. <https://doi.org/10.1016/j.socscimed.2005.08.016>

Lee, Y. J., Rauben, K., Liu, C., Kim, R., Van Der Velde, N., Taylor, C., Walsh, A., Asasira, M., Katongole, I., Hatfield-King, J., Blackwell, S., Iheanacho, T., Christ, R., & Ssekalo, I. (2022). Evaluation of a pilot, community-led mental illness de-stigmatization theater intervention in rural Uganda. *BMC Psychiatry*, *22*(1), 794. <https://doi.org/10.1186/s12888-022-04441-w>

Lelutiu-Weinberger, C., Clark, K. A., & Pachankis, J. E. (2022). Mental health provider training to improve LGBTQ competence and reduce implicit and explicit bias: A randomized controlled trial of online and in-person delivery. *Psychology of Sexual Orientation and Gender Diversity*. <https://doi.org/10.1037/sgd0000560>

Leluţiu-Weinberger, C., Rendina, H. J., Mirandola, M., Gios, L., Folch, C., Rafila, A., & Pachankis, J. E. (2019). The Role of Gay-Related Stigma in HIV-Risk Behavior Among Sexual Minority Men in Europe. *AIDS and Behavior*, *23*(3), 684–694. <https://doi.org/10.1007/s10461-018-2306-z>

Lewis, R. J., Mason, T. B., Winstead, B. A., Gaskins, M., & Irons, L. B. (2016). Pathways to Hazardous Drinking Among Racially and Socioeconomically Diverse Lesbian Women: Sexual Minority Stress, Rumination, Social Isolation, and Drinking to Cope. *Psychology of Women Quarterly*, *40*(4), 564–581. <https://doi.org/10.1177/0361684316662603>

Lewis, R. J., Winstead, B. A., Lau-Barraco, C., & Mason, T. B. (2017). Social Factors Linking Stigma-Related Stress with Alcohol Use among Lesbians: Stigma-related stress, Social Factors, and Alcohol Use. *Journal of Social Issues*, *73*(3), 545–562. <https://doi.org/10.1111/josi.12231>

Li, M. J., Takada, S., Okafor, C. N., Gorbach, P. M., Shoptaw, S. J., & Cole, S. W. (2020). Experienced homophobia and gene expression alterations in Black and Latino men who have sex with men in Los Angeles County. *Brain, Behavior, and Immunity*, *83*, 120–125. <https://doi.org/10.1016/j.bbi.2019.09.021>

Liegghio, M. (2017). Our Biggest Hurdle Yet: Caregivers’ Encounters with Structural Stigma in Child and Youth Mental Health. *Families in Society: The Journal of Contemporary Social Services*, *98*(4), 300–309. <https://doi.org/10.1606/1044-3894.2017.98.35>

Lin, C.-Y., Huang, Y.-T., Lee, C.-H., Fan, C.-W., & Yen, C.-F. (2022). Gay Community Stress Scale with Its Cultural Translation and Adaptions in Taiwan. *International Journal of Environmental Research and Public Health*, *19*(18), 11649. <https://doi.org/10.3390/ijerph191811649>

Lipperman-Kreda, S., Antin, T. M. J., & Hunt, G. P. (2019). The role of multiple social identities in discrimination and perceived smoking-related stigma among sexual and gender minority current or former smokers. *Drugs: Education, Prevention and Policy*, *26*(6), 475–483. <https://doi.org/10.1080/09687637.2018.1490391>

Livingston, J. D., Rossiter, K. R., & Verdun-Jones, S. N. (2011). ‘Forensic’ labelling: An empirical assessment of its effects on self-stigma for people with severe mental illness. *Psychiatry Research*, *188*(1), 115–122. <https://doi.org/10.1016/j.psychres.2011.01.018>

Lucey, T. K., & Grimm, K. (2021). Connecting political ecology of health and disease with ‘structural stigmatization’: Declining use of forest foods and medicines in Kédougou, Senegal. *Journal of Political Ecology*, *28*(1). <https://doi.org/10.2458/jpe.2996>

Lukachko, A., Hatzenbuehler, M. L., & Keyes, K. M. (2014). Structural racism and myocardial infarction in the United States. *Social Science & Medicine*, *103*, 42–50. <https://doi.org/10.1016/j.socscimed.2013.07.021>

Mann, S., O’Leary, N., & Blackaby, D. (2022). Sexual orientation, political trust, and same-sex relationship recognition policies: Evidence from Europe. *Public Choice*, *192*(3–4), 331–355. <https://doi.org/10.1007/s11127-022-00988-0>

Martin, S., Schofield, T., & Butterworth, P. (2022). News media representations of people receiving income support and the production of stigma power: An empirical analysis of reporting on two Australian welfare payments. *Critical Social Policy*, *42*(4), 648–670. <https://doi.org/10.1177/02610183211073945>

Martino, R. M., Weissman, D. G., McLaughlin, K. A., & Hatzenbuehler, M. L. (2023). Associations Between Structural Stigma and Psychopathology Among Early Adolescents. *Journal of Clinical Child & Adolescent Psychology*, 1–11. <https://doi.org/10.1080/15374416.2023.2272936>

Matson, T. E., Harris, A. H. S., Chen, J. A., Edmonds, A. T., Frost, M. C., Rubinsky, A. D., Blosnich, J. R., & Williams, E. C. (2022). Influence of a national transgender health care directive on receipt of alcohol-related care among transgender Veteran Health Administration patients with unhealthy alcohol use. *Journal of Substance Abuse Treatment*, *143*, 108808. <https://doi.org/10.1016/j.jsat.2022.108808>

Mauro, P. M., Philbin, M. M., Greene, E. R., Askari, M. S., & Martins, S. S. (2021). Medical and Non-Medical Opioid Use at the Intersection of Gender and Sexual Identity: Associations with State Medical Cannabis Law Status in a U.S. National Sample of Adults. *Archives of Sexual Behavior*, *50*(8), 3551–3561. <https://doi.org/10.1007/s10508-021-02128-z>

McKetta, S., Hughes, T. L., Zollweg, S. S., Matthews, A. K., Martin, K. R., & Veldhuis, C. B. (2023). The Modifying Effect of Minority Stressors on the Associations Between Neighborhood Deprivation and Mental Health Among US Sexual Minority Women. *Sexuality Research and Social Policy*. <https://doi.org/10.1007/s13178-023-00818-y>

McMillian-Bohler, J. M., Holt, L., Adimora, A. A., Bailey, D. “Chip” E., Johnson, R., Koch, A., McGee, K., Ramirez, C., Randolph, S. D., Ritchwood, T. D., & Relf, M. V. (2023). Examining Stigma and Disclosure Among Women With HIV in the Southern United States: Qualitative Study Guided by the Adaptive Leadership Framework for Chronic Illness. *Journal of the Association of Nurses in AIDS Care*, *34*(1), 113–124. <https://doi.org/10.1097/JNC.0000000000000354>

Meadows, A., Daníelsdóttir, S., Goldberg, D., & Mercedes, M. (2021). Fighting for a (wide enough) seat at the table: Weight stigma in law and policy. *Fat Studies*, *10*(2), 101–124. <https://doi.org/10.1080/21604851.2020.1835295>

Mellen, E. J., & Hatzenbuehler, M. L. (2023). Sexual Violence-Related Stigma, Mental Health, and Treatment-Seeking: A Multimodal Assessment in a Population-Based Study of Young Adults. *Journal of Interpersonal Violence*, *38*(19–20), 11243–11271. <https://doi.org/10.1177/08862605231179715>

Mereish, E. H., & Miranda, R. (2019). Exposure to Stigma Elicits Negative Affect and Alcohol Craving Among Young Adult Sexual Minority Heavy Drinkers. *Alcoholism: Clinical and Experimental Research*, acer.14055. <https://doi.org/10.1111/acer.14055>

Metzl, J. M., & Petty, J. (2017). Integrating and Assessing Structural Competency in an Innovative Prehealth Curriculum at Vanderbilt University: *Academic Medicine*, *92*(3), 354–359. <https://doi.org/10.1097/ACM.0000000000001477>

Meyerson, B., Barnes, P., Emetu, R., Bailey, M., Ohmit, A., & Gillespie, A. (2014). Institutional and Structural Barriers to HIV Testing: Elements for a Theoretical Framework. *AIDS Patient Care and STDs*, *28*(1), 22–27. <https://doi.org/10.1089/apc.2013.0238>

Miedema, S. S., Haardörfer, R., Keyes, C. L. M., & Yount, K. M. (2019). Does Socio-structural Context Matter? A Multilevel Test of Sexual Minority Stigma and Depressive Symptoms in Four Asia-Pacific Countries. *Journal of Health and Social Behavior*, *60*(4), 416–433. <https://doi.org/10.1177/0022146519877003>

Miller, C. T., Bunn, J. Y., Grover, K. W., & Solomon, S. E. (2014). Perceptions of behavioral norms related to HIV transmission by people with HIV and by residents of their communities. *Social Influence*, *9*(1), 1–19. <https://doi.org/10.1080/15534510.2012.712548>

Miller, C. T., Varni, S. E., Solomon, S. E., DeSarno, M. J., & Bunn, J. Y. (2016). Macro-level implicit HIV prejudice and the health of community residents with HIV. *Health Psychology*, *35*(8), 807–815. <https://doi.org/10.1037/hea0000314>

Miller, R. L., Strzyzykowski, T., Lee, K.-S., Chiaramonte, D., Acevedo-Polakovich, I., Spring, H., Santiago-Rivera, O., Boyer, C. B., & Ellen, J. M. (2018). Structural Effects on HIV Risk Among Youth: A Multi-level Analysis. *AIDS and Behavior*, *22*(11), 3451–3467. <https://doi.org/10.1007/s10461-018-2031-7>

Miller-Jacobs, C., Operario, D., & Hughto, J. M. W. (2023). State-Level Policies and Health Outcomes in U.S. Transgender Adolescents: Findings from the 2019 Youth Risk Behavior Survey. *LGBT Health*, *10*(6), 447–455. <https://doi.org/10.1089/lgbt.2022.0247>

Moody, R. L., Starks, T. J., Grov, C., & Parsons, J. T. (2018). Internalized Homophobia and Drug Use in a National Cohort of Gay and Bisexual Men: Examining Depression, Sexual Anxiety, and Gay Community Attachment as Mediating Factors. *Archives of Sexual Behavior*, *47*(4), 1133–1144. <https://doi.org/10.1007/s10508-017-1009-2>

Morey, B. N., Gee, G. C., Muennig, P., & Hatzenbuehler, M. L. (2018). Community-level prejudice and mortality among immigrant groups. *Social Science & Medicine*, *199*, 56–66. <https://doi.org/10.1016/j.socscimed.2017.04.020>

Mujugira, A., Kasiita, V., Bagaya, M., Nakyanzi, A., Bambia, F., Nampewo, O., Kamusiime, B., Mugisha, J., Nalumansi, A., Twesigye, C. C., Muwonge, T. R., Baeten, J. M., Wyatt, M. A., Tsai, A. C., Ware, N. C., & Haberer, J. E. (2021). “You are not a man”: A multi‐method study of trans stigma and risk of HIV and sexually transmitted infections among trans men in Uganda. *Journal of the International AIDS Society*, *24*(12). <https://doi.org/10.1002/jia2.25860>

Mukamana, D., Gishoma, D., Holt, L., Kayiranga, D., Na, J. J., White, R., Nyblade, L., Knettel, B. A., Agasaro, C., & Relf, M. V. (2022). Dehumanizing language, motherhood in the context of HIV, and overcoming HIV stigma - the voices of Rwandan women with HIV: A focus group study. *International Journal of Nursing Studies*, *135*, 104339. <https://doi.org/10.1016/j.ijnurstu.2022.104339>

Murray, S. M., Wiginton, J. M., Xue, Q. L., Dibble, K., Sanchez, T., Kane, J. C., Augustinavicius, J., Nowak, R. G., Crowell, T. A., Njindam, I. M., Tamoufe, U., Charurat, M., Turpin, G., Sithole, B., Mothopeng, T., Nemande, S., Simplice, A., Kouanda, S., Diouf, D., … Baral, S. (2023). Measuring sexual behavior stigma among cisgender men who have sex with men: An assessment of cross-country measurement invariance. *Stigma and Health*. <https://doi.org/10.1037/sah0000443>

Nardelli, N., Baiocco, R., Tanzilli, A., & Lingiardi, V. (2020). Not in the Same Mental Drawer: Internalized Sexual Stigma, Dissociation, and the Role of Religion in a Sample of Italian Gay Men. *Journal of Homosexuality*, *67*(10), 1386–1400. <https://doi.org/10.1080/00918369.2019.1591786>

Nelson, C. L., Wardecker, B. M., & Andel, R. (2023). Sexual Orientation and Gender Identity-Related State-Level Policies and Perceived Health Among Lesbian, Gay, Bisexual, and Transgender (LGBT) Older Adults in the United States. *Journal of Aging and Health*, *35*(3–4), 155–167. <https://doi.org/10.1177/08982643221116762>

Nelson, L. E., Ogunbajo, A., Abu-Ba’are, G. R., Conserve, D. F., Wilton, L., Ndenkeh, J. J., Braitstein, P., Dow, D., Arrington-Sanders, R., Appiah, P., Tucker, J., Nam, S., & Garofalo, R. (2022). Using the Implementation Research Logic Model as a Lens to View Experiences of Implementing HIV Prevention and Care Interventions with Adolescent Sexual Minority Men—A Global Perspective. *AIDS and Behavior*. <https://doi.org/10.1007/s10461-022-03776-5>

Nocka, K., Montgomery, M. C., Progovac, A., Guss, C. E., Chan, P. A., & Raifman, J. (2021). Primary Care for Transgender Adolescents and Young Adults in Rhode Island: An Analysis of the all Payers Claims Database. *Journal of Adolescent Health*, *68*(3), 472–479. <https://doi.org/10.1016/j.jadohealth.2020.11.014>

Norcini Pala, A., Dell’Amore, F., Steca, P., Clinton, L., Sandfort, T., & Rael, C. (2017). Validation of the Minority Stress Scale among Italian gay and bisexual men. *Psychology of Sexual Orientation and Gender Diversity*, *4*(4), 451–459. <https://doi.org/10.1037/sgd0000243>

O’Hara, C. A., Foon, X. L., Ng, J. C., Wong, C. S., Wang, F. Y., Tan, C. Y., Cheah, Y. T., Griva, K., Yoong, J. S., & Tan, R. K. (2023). Lesbian, gay, bisexual, transgender, queer and intersex (LGBTQI+) healthcare in Singapore: Perspectives of non-governmental organisations and clinical year medical students. *Medical Education Online*, *28*(1), 2172744. <https://doi.org/10.1080/10872981.2023.2172744>

Oldenburg, C. E., Perez-Brumer, A. G., Hatzenbuehler, M. L., Krakower, D., Novak, D. S., Mimiaga, M. J., & Mayer, K. H. (2015). State-level structural sexual stigma and HIV prevention in a national online sample of HIV-uninfected MSM in the United States. *AIDS*, *29*(7), 837–845. <https://doi.org/10.1097/QAD.0000000000000622>

Ong, W. J., Shahwan, S., Goh, C. M. J., Tan, G. T. H., Chong, S. A., & Subramaniam, M. (2020). Daily Encounters of Mental Illness Stigma and Individual Strategies to Reduce Stigma – Perspectives of People With Mental Illness. *Frontiers in Psychology*, *11*, 590844. <https://doi.org/10.3389/fpsyg.2020.590844>

Pachankis, J. E., & Bränström, R. (2018). Hidden from happiness: Structural stigma, sexual orientation concealment, and life satisfaction across 28 countries. *Journal of Consulting and Clinical Psychology*, *86*(5), 403–415. <https://doi.org/10.1037/ccp0000299>

Pachankis, J. E., & Bränström, R. (2019). How many sexual minorities are hidden? Projecting the size of the global closet with implications for policy and public health. *PLOS ONE*, *14*(6), e0218084. <https://doi.org/10.1371/journal.pone.0218084>

Pachankis, J. E., Eldahan, A. I., & Golub, S. A. (2016). New to New York: Ecological and Psychological Predictors of Health Among Recently Arrived Young Adult Gay and Bisexual Urban Migrants. *Annals of Behavioral Medicine*, *50*(5), 692–703. <https://doi.org/10.1007/s12160-016-9794-8>

Pachankis, J. E., Hatzenbuehler, M. L., Berg, R. C., Fernández-Dávila, P., Mirandola, M., Marcus, U., Weatherburn, P., & Schmidt, A. J. (2017). Anti-LGBT and Anti-immigrant Structural Stigma: An Intersectional Analysis of Sexual Minority Men’s HIV Risk When Migrating to or Within Europe. *JAIDS Journal of Acquired Immune Deficiency Syndromes*, *76*(4), 356–366. <https://doi.org/10.1097/QAI.0000000000001519>

Pachankis, J. E., Hatzenbuehler, M. L., Bränström, R., Schmidt, A. J., Berg, R. C., Jonas, K., Pitoňák, M., Baros, S., & Weatherburn, P. (2021). Structural stigma and sexual minority men’s depression and suicidality: A multilevel examination of mechanisms and mobility across 48 countries. *Journal of Abnormal Psychology*, *130*(7), 713–726. <https://doi.org/10.1037/abn0000693>

Pachankis, J. E., Hatzenbuehler, M. L., Hickson, F., Weatherburn, P., Berg, R. C., Marcus, U., & Schmidt, A. J. (2015). Hidden from health: Structural stigma, sexual orientation concealment, and HIV across 38 countries in the European MSM Internet Survey. *AIDS*, *29*(10), 1239–1246. <https://doi.org/10.1097/QAD.0000000000000724>

Pachankis, J. E., Hatzenbuehler, M. L., Mirandola, M., Weatherburn, P., Berg, R. C., Marcus, U., & Schmidt, A. J. (2017). The Geography of Sexual Orientation: Structural Stigma and Sexual Attraction, Behavior, and Identity Among Men Who Have Sex with Men Across 38 European Countries. *Archives of Sexual Behavior*, *46*(5), 1491–1502. <https://doi.org/10.1007/s10508-016-0819-y>

Pachankis, J. E., Hatzenbuehler, M. L., Rendina, H. J., Safren, S. A., & Parsons, J. T. (2015). LGB-affirmative cognitive-behavioral therapy for young adult gay and bisexual men: A randomized controlled trial of a transdiagnostic minority stress approach. *Journal of Consulting and Clinical Psychology*, *83*(5), 875–889. <https://doi.org/10.1037/ccp0000037>

Pachankis, J. E., Hatzenbuehler, M. L., & Starks, T. J. (2014). The influence of structural stigma and rejection sensitivity on young sexual minority men’s daily tobacco and alcohol use. *Social Science & Medicine*, *103*, 67–75. <https://doi.org/10.1016/j.socscimed.2013.10.005>

Pachankis, J. E., McConocha, E. M., Clark, K. A., Wang, K., Behari, K., Fetzner, B. K., Brisbin, C. D., Scheer, J. R., & Lehavot, K. (2020). A transdiagnostic minority stress intervention for gender diverse sexual minority women’s depression, anxiety, and unhealthy alcohol use: A randomized controlled trial. *Journal of Consulting and Clinical Psychology*, *88*(7), 613–630. <https://doi.org/10.1037/ccp0000508>

Pachankis, J. E., Soulliard, Z. A., Layland, E. K., Behari, K., Seager Van Dyk, I., Eisenstadt, B. E., Chiaramonte, D., Ljótsson, B., Särnholm, J., & Bjureberg, J. (2023). Guided LGBTQ-affirmative internet cognitive-behavioral therapy for sexual minority youth’s mental health: A randomized controlled trial of a minority stress treatment approach. *Behaviour Research and Therapy*, *169*, 104403. <https://doi.org/10.1016/j.brat.2023.104403>

Parmenter, J. G., & Winter, S. D. (2023). Inequity within the lesbian, gay, bisexual, transgender, and queer (LGBTQ+) community as a distal stressor: An extension of minority stress theory. *Psychology of Sexual Orientation and Gender Diversity*. <https://doi.org/10.1037/sgd0000674>

Passell, E., Rutter, L. A., Turban, J. L., Scheuer, L., Wright, N., & Germine, L. (2022). Generalized Anxiety Disorder Symptoms are Higher Among Same- and Both-Sex Attracted Individuals in a Large, International Sample. *Sexuality Research and Social Policy*, *19*(4), 1440–1451. <https://doi.org/10.1007/s13178-021-00637-z>

Paterson, B., Hirsch, G., & Andres, K. (2013). Structural factors that promote stigmatization of drug users with hepatitis C in hospital emergency departments. *International Journal of Drug Policy*, *24*(5), 471–478. <https://doi.org/10.1016/j.drugpo.2013.01.008>

Pellicane, M. J., & Ciesla, J. A. (2022). Temporal trends in rates of depression, anxiety, and suicidality among cisgender sexual minority and heterosexual college students. *Psychology of Sexual Orientation and Gender Diversity*. <https://doi.org/10.1037/sgd0000563>

Perales, F. (2016). The Costs of Being “Different”: Sexual Identity and Subjective Wellbeing over the Life Course. *Social Indicators Research*, *127*(2), 827–849. <https://doi.org/10.1007/s11205-015-0974-x>

Perales, F., & Todd, A. (2018). Structural stigma and the health and wellbeing of Australian LGB populations: Exploiting geographic variation in the results of the 2017 same-sex marriage plebiscite. *Social Science & Medicine*, *208*, 190–199. <https://doi.org/10.1016/j.socscimed.2018.05.015>

Perez-Brumer, A., Hatzenbuehler, M. L., Oldenburg, C. E., & Bockting, W. (2015). Individual- and Structural-Level Risk Factors for Suicide Attempts Among Transgender Adults. *Behavioral Medicine*, *41*(3), 164–171. <https://doi.org/10.1080/08964289.2015.1028322>

Perrin, E. C., Hurley, S. M., Mattern, K., Flavin, L., & Pinderhughes, E. E. (2019). Barriers and Stigma Experienced by Gay Fathers and Their Children. *Pediatrics*, *143*(2), e20180683. <https://doi.org/10.1542/peds.2018-0683>

Pharr, J. R., Chien, L.-C., Gakh, M., Flatt, J. D., Kittle, K., & Terry, E. (2022a). Moderating Effect of Community and Individual Resilience on Structural Stigma and Suicidal Ideation among Sexual and Gender Minority Adults in the United States. *International Journal of Environmental Research and Public Health*, *19*(21), 14526. <https://doi.org/10.3390/ijerph192114526>

Pharr, J. R., Chien, L.-C., Gakh, M., Flatt, J., Kittle, K., & Terry, E. (2022b). Serial Mediation Analysis of the Association of Familiarity with Transgender Sports Bans and Suicidality among Sexual and Gender Minority Adults in the United States. *International Journal of Environmental Research and Public Health*, *19*(17), 10641. <https://doi.org/10.3390/ijerph191710641>

Philbin, M. M., Greene, E. R., Martins, S. S., LaBossier, N. J., & Mauro, P. M. (2020). Medical, Nonmedical, and Illegal Stimulant Use by Sexual Identity and Gender. *American Journal of Preventive Medicine*, *59*(5), 686–696. <https://doi.org/10.1016/j.amepre.2020.05.025>

Philbin, M. M., Parker, C. M., Parker, R. G., Wilson, P. A., Garcia, J., & Hirsch, J. S. (2016). The Promise of Pre-Exposure Prophylaxis for Black Men Who Have Sex with Men: An Ecological Approach to Attitudes, Beliefs, and Barriers. *AIDS Patient Care and STDs*, *30*(6), 282–290. <https://doi.org/10.1089/apc.2016.0037>

Philbin, M. M., Wurtz, H. M., McCrimmon, T., Kelly, E., Homan, P., & Guta, A. (2023). How social policies shape the health and well-being of sexual- and gender-minority youth: Pathways of influence, social side effects and implications for life course trajectories. *Social Science & Medicine*, *317*, 115624. <https://doi.org/10.1016/j.socscimed.2022.115624>

Phillips, T. R., Medland, N., Chow, E. P. F., Maddaford, K., Wigan, R., Fairley, C. K., Ong, J. J., & Bilardi, J. E. (2020). “Moving from one environment to another, it doesn’t automatically change everything”. Exploring the transnational experience of Asian-born gay and bisexual men who have sex with men newly arrived in Australia. *PLOS ONE*, *15*(11), e0242788. <https://doi.org/10.1371/journal.pone.0242788>

Pianchob, S., Muangpaisan, W., & Siritipakorn, P. (2017). Factor Analysis and Reliability of the Family Stigma in Alzheimer’s Disease Scale-Thai Version. *Journal of the Medical Association of Thailand*, *100*(12), 1318–1324.

Pitoňák, M., Kožený, J., & Čihák, M. (2023). Disparities in Psychological Distress between Czech General Population and LGB + Community Sample. *Journal of Bisexuality*, *23*(2), 151–169. <https://doi.org/10.1080/15299716.2023.2191590>

Pu, Y., & Xu, W. (2023). Parenting Desire Among Sexual Minority Women in China: From the Stigma Perspective. *Archives of Sexual Behavior*. <https://doi.org/10.1007/s10508-023-02682-8>

Purtle, J., Lê-Scherban, F., Wang, X., Shattuck, P. T., Proctor, E. K., & Brownson, R. C. (2018). Audience segmentation to disseminate behavioral health evidence to legislators: An empirical clustering analysis. *Implementation Science*, *13*(1), 121. <https://doi.org/10.1186/s13012-018-0816-8>

Qasim, S., McKeown, M., Kunda, C., Wainwright, J. P., & Khan, R. (2020). Plant Fetish: A Creative Challenge to Mental Health Stigma. *Genealogy*, *4*(2), 40. <https://doi.org/10.3390/genealogy4020040>

Raifman, J., Cheng, D. M., Skinner, A., Hatzenbuehler, M. L., Mayer, K. H., & Stein, M. D. (2023). State same‐sex marriage policies and pre‐exposure prophylaxis implementation among men who have sex with men in the United States. *Journal of the International AIDS Society*, *26*(11), e26180. <https://doi.org/10.1002/jia2.26180>

Raifman, J., Nocka, K., Galárraga, O., Wilson, I. B., Crowley, C., Tao, J., Napoleon, S., Marak, T., & Chan, P. A. (2020). Evaluating statewide HIV preexposure prophylaxis implementation using All-Payer Claims Data. *Annals of Epidemiology*, *44*, 1-7.e2. <https://doi.org/10.1016/j.annepidem.2020.03.003>

Ramos-Pibernus, A. G., Rivera-Segarra, E. R., Rodríguez-Madera, S. L., Varas-Díaz, N., & Padilla, M. (2020). Stigmatizing Experiences of Trans Men in Puerto Rico: Implications for Health. *Transgender Health*, *5*(4), 234–240. <https://doi.org/10.1089/trgh.2020.0021>

Rao, S., Mason, C. D., Galvao, R. W., Clark, B. A., & Calabrese, S. K. (2020). “You are illegal in your own country”: The perceived impact of antisodomy legislation among Indian sexual and gender minorities. *Stigma and Health*, *5*(4), 451–462. <https://doi.org/10.1037/sah0000218>

Reber, L., Kreschmer, J. M., James, T. G., Junior, J. D., DeShong, G. L., Parker, S., & Meade, M. A. (2022). Ableism and Contours of the Attitudinal Environment as Identified by Adults with Long-Term Physical Disabilities: A Qualitative Study. *International Journal of Environmental Research and Public Health*, *19*(12), 7469. <https://doi.org/10.3390/ijerph19127469>

Regnerus, M. (2017). Is structural stigma’s effect on the mortality of sexual minorities robust? A failure to replicate the results of a published study. *Social Science & Medicine*, *188*, 157–165. <https://doi.org/10.1016/j.socscimed.2016.11.018>

Reid, A. E., Dovidio, J. F., Ballester, E., & Johnson, B. T. (2014). HIV prevention interventions to reduce sexual risk for African Americans: The influence of community-level stigma and psychological processes. *Social Science & Medicine*, *103*, 118–125. <https://doi.org/10.1016/j.socscimed.2013.06.028>

Reimer-Kirkham, S., Astle, B., Ero, I., Imafidon, E., & Strobell, E. (2022). Mothering, Albinism and Human Rights: The Disproportionate Impact of Health-Related Stigma in Tanzania. *Foundations of Science*, *27*(2), 719–740. <https://doi.org/10.1007/s10699-020-09701-0>

Ricciardelli, R., Carleton, R. N., Mooney, T., & Cramm, H. (2020). “Playing the system”: Structural factors potentiating mental health stigma, challenging awareness, and creating barriers to care for Canadian public safety personnel. *Health: An Interdisciplinary Journal for the Social Study of Health, Illness and Medicine*, *24*(3), 259–278. <https://doi.org/10.1177/1363459318800167>

Riggle, E. D. B., Drabble, L., Veldhuis, C. B., Wootton, A., & Hughes, T. L. (2018). The Impact of Marriage Equality on Sexual Minority Women’s Relationships With Their Families of Origin. *Journal of Homosexuality*, *65*(9), 1190–1206. <https://doi.org/10.1080/00918369.2017.1407611>

Ritterbusch, A. E., Correa Salazar, C., & Correa, A. (2018). Stigma-related access barriers and violence against trans women in the Colombian healthcare system. *Global Public Health*, *13*(12), 1831–1845. <https://doi.org/10.1080/17441692.2018.1455887>

Robles, G., Lee, J. J., Yu, M., & Starks, T. J. (2023). Multilevel Analysis of Sociopolitical Contexts, Social Support, Mental Health, and Alcohol Use Among Partnered Sexual Minority Latino Men in the U.S. *Journal of Racial and Ethnic Health Disparities*. <https://doi.org/10.1007/s40615-023-01637-y>

Rosenberg, S. (2018). Coming In: Queer Narratives of Sexual Self-Discovery. *Journal of Homosexuality*, *65*(13), 1788–1816. <https://doi.org/10.1080/00918369.2017.1390811>

Rucco, D., Anzani, A., Scandurra, C., Pennasilico, A., & Prunas, A. (2023). Structural Stigma and Bisexual + People: Effects of the Rejection of the Zan Bill in Italy on Minority Stress and Mental Health. *Journal of Bisexuality*, *23*(1), 27–49. <https://doi.org/10.1080/15299716.2022.2119629>

Saewyc, E. M., Li, G., Gower, A. L., Watson, R. J., Erickson, D., Corliss, H. L., & Eisenberg, M. E. (2020). The link between LGBTQ-supportive communities, progressive political climate, and suicidality among sexual minority adolescents in Canada. *Preventive Medicine*, *139*, 106191. <https://doi.org/10.1016/j.ypmed.2020.106191>

Samari, G., Catalano, R., Alcalá, H. E., & Gemmill, A. (2020). The Muslim Ban and preterm birth: Analysis of U.S. vital statistics data from 2009 to 2018. *Social Science & Medicine*, *265*, 113544. <https://doi.org/10.1016/j.socscimed.2020.113544>

Sarno, E. L., Smith, M. S., & Newcomb, M. E. (2023). Minority stress and intimate partner aggression among male same‐sex couples. *Personal Relationships*, *30*(3), 756–772. <https://doi.org/10.1111/pere.12482>

Saxby, K., Chan, C., & Bavinton, B. R. (2022). Structural Stigma and Sexual Health Disparities Among Gay, Bisexual, and Other Men Who Have Sex With Men in Australia. *JAIDS Journal of Acquired Immune Deficiency Syndromes*, *89*(3), 241–250. <https://doi.org/10.1097/QAI.0000000000002851>

Saxby, K., de New, S. C., & Petrie, D. (2020). Structural stigma and sexual orientation disparities in healthcare use: Evidence from Australian Census-linked-administrative data. *Social Science & Medicine*, *255*, 113027. <https://doi.org/10.1016/j.socscimed.2020.113027>

Scheer, J. R., Pachankis, J. E., & Bränström, R. (2022). Gender-based Structural Stigma and Intimate Partner Violence Across 28 Countries: A Population-based Study of Women Across Sexual Orientation, Immigration Status, and Socioeconomic Status. *Journal of Interpersonal Violence*, *37*(11–12), NP8941–NP8964. <https://doi.org/10.1177/0886260520976212>

Schlehofer, M. M., Wagner, K., & Bramande, E. (2023). “Things Will Get Worse Before They Get Better”: LGBTQ + People’s Reactions to the 2020 US Presidential Election. *Sexuality Research and Social Policy*, *20*(4), 1378–1392. <https://doi.org/10.1007/s13178-023-00802-6>

Schmitz, R. M., Robinson, B. A., Tabler, J., Welch, B., & Rafaqut, S. (2020). LGBTQ+ Latino/a Young People’s Interpretations of Stigma and Mental Health: An Intersectional Minority Stress Perspective. *Society and Mental Health*, *10*(2), 163–179. <https://doi.org/10.1177/2156869319847248>

Schuler, M. S., Rice, C. E., Evans-Polce, R. J., & Collins, R. L. (2018). Disparities in substance use behaviors and disorders among adult sexual minorities by age, gender, and sexual identity. *Drug and Alcohol Dependence*, *189*, 139–146. <https://doi.org/10.1016/j.drugalcdep.2018.05.008>

Seager Van Dyk, I., Aldao, A., & Pachankis, J. E. (2022). Coming out under fire: The role of minority stress and emotion regulation in sexual orientation disclosure. *PLOS ONE*, *17*(5), e0267810. <https://doi.org/10.1371/journal.pone.0267810>

Shattuck, D., Richard, B. O., Jaramillo, E. T., Byrd, E., & Willging, C. E. (2022). Power and resistance in schools: Implementing institutional change to promote health equity for sexual and gender minority youth. *Frontiers in Health Services*, *2*, 920790. <https://doi.org/10.3389/frhs.2022.920790>

Skinner, A., Stein, M. D., Dean, L. T., Oldenburg, C. E., Mimiaga, M. J., Chan, P. A., Mayer, K. H., & Raifman, J. (2023). Same-Sex Marriage Laws, Provider-Patient Communication, and PrEP Awareness and Use Among Gay, Bisexual, and Other Men Who have Sex with Men in the United States. *AIDS and Behavior*, *27*(6), 1897–1905. <https://doi.org/10.1007/s10461-022-03923-y>

Soffer, M. (2022). Cancer-related stigma in the USA and Israeli mass media: An exploratory study of structural stigma. *Journal of Cancer Survivorship*, *16*(1), 213–222. <https://doi.org/10.1007/s11764-021-01145-0>

Solazzo, A. L., Austin, S. B., Rosario, M., Corliss, H. L., & Charlton, B. M. (2020). Maternal Comfort with Lesbian, Gay, and Bisexual People and Their Children’s Drinking, Smoking, and Disordered Weight Control Behaviors as Adults. *LGBT Health*, *7*(7), 375–384. <https://doi.org/10.1089/lgbt.2019.0315>

Starks, T. J., Hillesheim, J. R., Stephenson, R., & Robles, G. (2023). Policy, relationships, and well-being: Associations between mental health and main partner relationships among cisgender sexual minority men in the context of U.S. state-level policies. *Psychology of Sexual Orientation and Gender Diversity*. <https://doi.org/10.1037/sgd0000648>

Stevens, M. E., Parsons, J. A., Read, S. E., Bond, V., Solomon, P., & Nixon, S. A. (2021). The relationship between stigma and a rehabilitation framework [international classification of functioning, disability and health (ICF)]: Three case studies of women living with HIV in Lusaka, Zambia. *Disability and Rehabilitation*, *43*(15), 2149–2156. <https://doi.org/10.1080/09638288.2019.1693640>

Stojanovski, K., King, E. J., Amico, K. R., Eisenberg, M. C., Geronimus, A. T., Baros, S., & Schmidt, A. J. (2022). Stigmatizing Policies Interact with Mental Health and Sexual Behaviours to Structurally Induce HIV Diagnoses Among European Men Who Have Sex with Men. *AIDS and Behavior*, *26*(10), 3400–3410. <https://doi.org/10.1007/s10461-022-03683-9>

Subramaniam, M., Zhang, Y., Shahwan, S., Vaingankar, J. A., Satghare, P., Teh, W. L., Roystonn, K., Goh, C. M. J., Maniam, Y., Tan, Z. L., Tay, B., Verma, S., & Chong, S. A. (2022). Employment of young people with mental health conditions: Making it work. *Disability and Rehabilitation*, *44*(10), 2033–2043. <https://doi.org/10.1080/09638288.2020.1822932>

Sukhera, J., Kulkarni, C., & Taylor, T. (2021). Structural distress: Experiences of moral distress related to structural stigma during the COVID-19 pandemic. *Perspectives on Medical Education*, *10*(4), 222–229. <https://doi.org/10.1007/S40037-021-00663-Y>

Sukhera, J., Poleksic, J., Zaheer, J., & Pack, R. (2022). Normalising disclosure or reinforcing heroism? An exploratory critical discourse analysis of mental health stigma in medical education. *Medical Education*, *56*(8), 823–833. <https://doi.org/10.1111/medu.14790>

Syvertsen, J. L., Toneff, H., Howard, H., Spadola, C., Madden, D., & Clapp, J. (2021). Conceptualizing stigma in contexts of pregnancy and opioid misuse: A qualitative study with women and healthcare providers in Ohio. *Drug and Alcohol Dependence*, *222*, 108677. <https://doi.org/10.1016/j.drugalcdep.2021.108677>

Taylor, C. J. (2014). Physiological stress response to loss of social influence and threats to masculinity. *Social Science & Medicine*, *103*, 51–59. <https://doi.org/10.1016/j.socscimed.2013.07.036>

Titus, A. R., Gamarel, K. E., Thrasher, J. F., Meza, R., & Fleischer, N. L. (2021). State-Level Structural Stigma and Smoking Among Sexual Minority Adults in the USA, 2012–2014. *Annals of Behavioral Medicine*, *55*(6), 557–570. <https://doi.org/10.1093/abm/kaaa086>

Tran, N. K., Baker, K. E., Lett, E., & Scheim, A. I. (2022). State-level heterogeneity in associations between structural stigma and individual healthcare access: A multilevel analysis of transgender adults in the United States. *Journal of Health Services Research & Policy*, 135581962211234. <https://doi.org/10.1177/13558196221123413>

Trani, J.-F., Ballard, E., & Peña, J. B. (2016). Stigma of persons with disabilities in Afghanistan: Examining the pathways from stereotyping to mental distress. *Social Science & Medicine*, *153*, 258–265. <https://doi.org/10.1016/j.socscimed.2016.02.024>

Trani, J.-F., Moodley, J., Anand, P., Graham, L., & Thu Maw, M. T. (2020). Stigma of persons with disabilities in South Africa: Uncovering pathways from discrimination to depression and low self-esteem. *Social Science & Medicine*, *265*, 113449. <https://doi.org/10.1016/j.socscimed.2020.113449>

Tudose, C., Sfetcu, R., Dobre, C., & Moglan, M. (2017). Structural Stigma, Affiliated Stigma and Consequences of Caregiving for the Family Members of Inpatients with Dementia: A Comparative Approach. *Revista de Cercetare Si Interventie Sociala*, *57*, 148–158.

Tyler, T. R. (2015). Our Story: The Parent and LGBTQ Child Relational Process. *Journal of Gay & Lesbian Social Services*, *27*(1), 17–45. <https://doi.org/10.1080/10538720.2015.988313>

Ugwu, U. T., & Dumbili, E. W. (2022). Inhaling thick smoke: Cannabis subculture, community forming and socio-structural challenges in Nigeria. *Drugs: Education, Prevention and Policy*, *29*(4), 345–354. <https://doi.org/10.1080/09687637.2021.2004998>

Ünsal, B. C., Demetrovics, Z., & Reinhardt, M. (2023). Stronger together: Community participation, structural stigma, and depression among sexual and gender minority adults in 28 European countries. *Social Psychiatry and Psychiatric Epidemiology*, *58*(4), 657–669. <https://doi.org/10.1007/s00127-022-02385-w>

van der Star, A., Bränström, R., & Pachankis, J. E. (2021). Lifecourse-varying structural stigma, minority stress reactions and mental health among sexual minority male migrants. *European Journal of Public Health*, *31*(4), 803–808. <https://doi.org/10.1093/eurpub/ckab032>

van der Star, A., Pachankis, J. E., & Bränström, R. (2021). Country-Level Structural Stigma, School-Based and Adulthood Victimization, and Life Satisfaction Among Sexual Minority Adults: A Life Course Approach. *Journal of Youth and Adolescence*, *50*(1), 189–201. <https://doi.org/10.1007/s10964-020-01340-9>

Veldhuis, C. B., Drabble, L., Riggle, E. D. B., Wootton, A. R., & Hughes, T. L. (2018). “We Won’t Go Back into the Closet Now Without One Hell of a Fight”: Effects of the 2016 Presidential Election on Sexual Minority Women’s and Gender Minorities’ Stigma-Related Concerns. *Sexuality Research and Social Policy*, *15*(1), 12–24. <https://doi.org/10.1007/s13178-017-0305-x>

Villalona, S. (2021). Insights from the shadows: Exploring deservingness of care in the emergency department and language as a social determinant of health. *Medical Humanities*, *47*(3), e5–e5. <https://doi.org/10.1136/medhum-2019-011669>

Villanueva Baselga, S. (2020). Interactive documentaries and health: Combating HIV-related stigma and cultural trauma. *Catalan Journal of Communication & Cultural Studies*, *12*(2), 273–285. <https://doi.org/10.1386/cjcs_00032_1>

Wainberg, M. L., Cournos, F., Wall, M. M., Norcini Pala, A., Mann, C. G., Pinto, D., Pinho, V., & McKinnon, K. (2016). Mental illness sexual stigma: Implications for health and recovery. *Psychiatric Rehabilitation Journal*, *39*(2), 90–96. <https://doi.org/10.1037/prj0000168>

Wardecker, B. M., Surachman, A., Matsick, J. L., & Almeida, D. M. (2022). Daily Stressor Exposure and Daily Well-Being Among Sexual Minority and Heterosexual Adults in the United States: Results from the National Study of Daily Experiences (NSDE). *Annals of Behavioral Medicine*, *56*(6), 536–550. <https://doi.org/10.1093/abm/kaab062>

Warren, A. (2023). The relationship between perceived stigma and perceived stress in cognitive decline: A survey of persons with mild cognitive impairment and their caregivers. *Frontiers in Psychology*, *14*, 1293284. <https://doi.org/10.3389/fpsyg.2023.1293284>

Weeks, S. N., Renshaw, T. L., & Vinal, S. A. (2023). Minority Stress as a Multidimensional Predictor of LGB+ Adolescents’ Mental Health Outcomes. *Journal of Homosexuality*, *70*(5), 938–962. <https://doi.org/10.1080/00918369.2021.2006000>

Werner, P., & Doron, I. (Issi). (2017). Alzheimer’s disease and the law: Positive and negative consequences of structural stigma and labeling in the legal system. *Aging & Mental Health*, *21*(11), 1206–1213. <https://doi.org/10.1080/13607863.2016.1211989>

Werner, P., Goldstein, D., & Buchbinder, E. (2010). Subjective Experience of Family Stigma as Reported by Children of Alzheimer’s Disease Patients. *Qualitative Health Research*, *20*(2), 159–169. <https://doi.org/10.1177/1049732309358330>

Werner, P., Goldstein, D., & Heinik, J. (2011). Development and Validity of the Family Stigma in Alzheimer’s Disease Scale (FS-ADS). *Alzheimer Disease & Associated Disorders*, *25*(1), 42–48. <https://doi.org/10.1097/WAD.0b013e3181f32594>

Werner, P., Mittelman, M. S., Goldstein, D., & Heinik, J. (2012). Family Stigma and Caregiver Burden in Alzheimer’s Disease. *The Gerontologist*, *52*(1), 89–97. <https://doi.org/10.1093/geront/gnr117>

White Hughto, J. M., Clark, K. A., Altice, F. L., Reisner, S. L., Kershaw, T. S., & Pachankis, J. E. (2018). Creating, reinforcing, and resisting the gender binary: A qualitative study of transgender women’s healthcare experiences in sex-segregated jails and prisons. *International Journal of Prisoner Health*, *14*(2), 69–88. <https://doi.org/10.1108/IJPH-02-2017-0011>

Whiteman, A., Baugher, A., & Sionean, C. (2021). Assessing self-reported discrimination among men who have sex with men (MSM). *AIDS*, *35*(1), 141–146. <https://doi.org/10.1097/QAD.0000000000002711>

Whittle, H. J., Palar, K., Ranadive, N. A., Turan, J. M., Kushel, M., & Weiser, S. D. (2017). “The land of the sick and the land of the healthy”: Disability, bureaucracy, and stigma among people living with poverty and chronic illness in the United States. *Social Science & Medicine*, *190*, 181–189. <https://doi.org/10.1016/j.socscimed.2017.08.031>

Winskell, K., & Sabben, G. (2016). Sexual stigma and symbolic violence experienced, enacted, and counteracted in young Africans’ writing about same-sex attraction. *Social Science & Medicine*, *161*, 143–150. <https://doi.org/10.1016/j.socscimed.2016.06.004>

Woodford, M. R., Kulick, A., Garvey, J. C., Sinco, B. R., & Hong, J. S. (2018). LGBTQ policies and resources on campus and the experiences and psychological well-being of sexual minority college students: Advancing research on structural inclusion. *Psychology of Sexual Orientation and Gender Diversity*, *5*(4), 445–456. <https://doi.org/10.1037/sgd0000289>

Woodgate, R. L., Comaskey, B., Tennent, P., Wener, P., & Altman, G. (2020). The Wicked Problem of Stigma for Youth Living With Anxiety. *Qualitative Health Research*, *30*(10), 1491–1502. <https://doi.org/10.1177/1049732320916460>

Xu, W., Huang, Y., Tang, W., & Kaufman, M. R. (2022). Heterosexual Marital Intention: The Influences of Confucianism and Stigma Among Chinese Sexual Minority Women and Men. *Archives of Sexual Behavior*, *51*(7), 3529–3540. <https://doi.org/10.1007/s10508-021-02229-9>

Ylioja, T., Cochran, G., Woodford, M. R., & Renn, K. A. (2016). Frequent Experience of LGBQ Microaggression on Campus Associated With Smoking Among Sexual Minority College Students. *Nicotine & Tobacco Research*, ntw305. <https://doi.org/10.1093/ntr/ntw305>

Young, I., & Valiotis, G. (2020). Strategies to support HIV literacy in the roll-out of pre-exposure prophylaxis in Scotland: Findings from qualitative research with clinical and community practitioners. *BMJ Open*, *10*(4), e033849. <https://doi.org/10.1136/bmjopen-2019-033849>

Zelin, N. S., Solotke, M. T., Scott, C. E., Atienza-Carbonell, B., Fogas, C., Skrzypczak, J., Starin, R., Tamburelli, F., Ucar, A., & Pelzer, B. W. (2020). An Analysis of the Presence and Composition of OutLists at United States, Canadian, and European Medical Institutions. *Journal of Homosexuality*, *67*(14), 1999–2013. <https://doi.org/10.1080/00918369.2019.1621554>
